# Lanthanide Hexacyanidoruthenate Frameworks for Multicolor to White-Light Emission Realized by the Combination of d-d, d-f, and f-f Electronic Transitions

**DOI:** 10.1021/acs.inorgchem.2c03885

**Published:** 2023-01-19

**Authors:** Tomasz Charytanowicz, Barbara Sieklucka, Szymon Chorazy

**Affiliations:** Faculty of Chemistry, Jagiellonian University, Gronostajowa 2, 30-387 Krakow, Poland

## Abstract

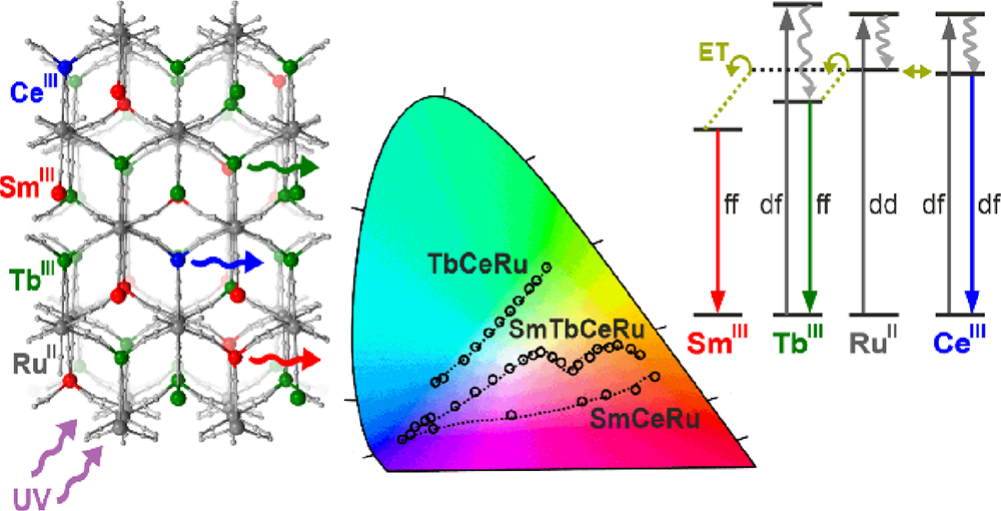

We report an effective strategy toward tunable room-temperature multicolor to white-light emission realized by mixing three different lanthanide ions (Sm^3+^, Tb^3+^, and Ce^3+^) in three-dimensional (3D) coordination frameworks based on hexacyanidoruthenate(II) metalloligands. Mono-lanthanide compounds, K{Ln^III^(H_2_O)_*n*_[Ru^II^(CN)_6_]}·*m*H_2_O (**1**, Ln = La, *n* = 3, *m* = 1.2; **2**, Ln = Ce, *n* = 3, *m* = 1.3; **3**, Ln = Sm, *n* = 2, *m* = 2.4; **4**, Ln = Tb, *n* = 2, *m* = 2.4) are 3D cyanido-bridged networks based on the Ln–NC–Ru linkages, with cavities occupied by K^+^ ions and water molecules. They crystallize differently for larger (**1**, **2**) and smaller (**3**, **4**) lanthanides, in the hexagonal *P*6_3_/m or the orthorhombic *C*mcm space groups, respectively. All exhibit luminescence under the UV excitation, including weak blue emission in **1** due to the d-d ^3^T_1g_ → ^1^A_1g_ electronic transition of Ru^II^, as well as much stronger blue emission in **2** related to the d-f ^2^D_3/2_ → ^2^F_5/2,7/2_ transitions of Ce^III^, red emission in **3** due to the f-f ^4^G_5/2_ → ^6^H_5/2,7/2,9/2,11/2_ transitions of Sm^III^, and green emission in **4** related to the f-f ^5^D_4_ → ^7^F_6,5,4,3_ transitions of Tb^III^. The lanthanide emissions, especially those of Sm^III^, take advantage of the Ru^II^-to-Ln^III^ energy transfer. The Ce^III^ and Tb^III^ emissions are also supported by the excitation of the d-f electronic states. Exploring emission features of the Ln^III^–Ru^II^ networks, two series of heterobi-lanthanide systems, K{Sm_*x*_Ce_1–*x*_(H_2_O)_*n*_[Ru(CN)_6_]}·*m*H_2_O (*x* = 0.47, 0.88, 0.88, 0.99, 0.998; **5**–**9**) and K{Tb_*x*_Ce_1–*x*_(H_2_O)_*n*_[Ru(CN)_6_]}·*m*H_2_O (*x* = 0.56, 0.65, 0.93, 0.99, 0.997; **10**–**14**) were prepared. They exhibit the composition- and excitation-dependent tuning of emission from blue to red and blue to green, respectively. Finally, the heterotri-lanthanide system of the K{Sm_0.4_Tb_0.599_Ce_0.001_(H_2_O)_2_[Ru(CN)_6_]}·2.5H_2_O (**15**) composition shows the rich emission spectrum consisting of the peaks related to Ce^III^, Tb^III^, and Sm^III^ centers, which gives the emission color tuning from blue to orange and white-light emission of the CIE 1931 *xy* parameters of 0.325, 0.333.

## Abstract 2

Red-emissive Sm^3+^, green-emissive Tb^3+^, and blue-emissive Ce^3+^ ions were combined with blue-emissive hexacyanidoruthenate(II) metalloligands into three-dimensional cyanido-bridged frameworks. They exhibit rich room-temperature multicolor-to-white-light emission tuned by the 4f-metal composition and excitation wavelength. The optical functionalities are generated by the optimized emission contributions from d-f electronic transitions of Ce^3+^ and f-f electronic transitions of Sm^3+^ and Tb^3+^, the latter governed by the Ru^II^-to-Ln^III^ energy transfer utilizing the d-d electronic transitions of the hexacyanidometallate complex.

## Introduction

Two luminescence functionalities, namely, tunable multicolored emission and white-light emission, attract great attention due to their wide applications in flat-panel displays and solid-state lighting, especially when the related light-emitting diodes (LEDs) are constructed,^1−4^ as well as in optical sensing, labeling, imaging, and anticounterfeiting technologies.^5−8^ There are multiple approaches toward the efficient tuning of multicolor emission, usually realized by playing with excitation-dependent photoluminescence.^9−18^ This includes the exploration of organic molecules and polymers,^9−11^ inorganic or hybrid semiconductors,^12−14^ metal–organic assemblies,^15−17^ and composite systems.^18^ On the other hand, white-light emission (WLE) was traditionally generated for the LED systems using a blue LED covered by a yellow emitter or mixing materials providing red, green, and blue emission components.^19−21^ These methodologies provide technical problems related to such effects as phase separation, high cost, the complicated technique for linking several components, etc. The alternative lies in the single-phase white-light-emitting (SPWLE) materials, which appear to be a more convenient route to the fabrication of high-performance white light-emitting diodes (WLEDs).^22,23^ They can be constructed by the series of luminophores inserted in organic polymers,^24^ hybrid semiconductors showing broadband emission,^25^ and lanthanide-doped inorganic matrices.^26−28^ In this context, both tunable multicolored emission and SPWLE phenomenon can be achieved using coordination polymers (CPs)^29−31^ or polynuclear metal-based molecules.^32,33^ They take advantage of various photoluminescence sources, including ligand-centered (LC), metal-centered (MC), excimer/exciplex-based, or metal-to-ligand/ligand-to-metal charge transfer-based (MLCT/LMCT) emissions.^34−36^ Moreover, coordination assemblies are built of various organic and inorganic components, offering often also the capability to incorporate additional guest molecules,^37^ and thus they provide multiple emitting centers in a single phase, which ensures the route to the emission color tuning and the WLE.^29−36^ Of particular interest are the coordination systems based on trivalent lanthanide ions, which, under the UV light excitation, exhibit characteristic emission peaks related to f-f electronic transitions.^38^ These emissions are particularly strong for Eu^3+^ (red emission) and Tb^3+^ (green emission) ions, observed also in the visible range for other ions, such as Sm^3+^ (red emission) or Dy^3+^ (yellow emission).^39−42^ As the f-f electronic transitions are forbidden, the direct excitation is usually limited; however, it can be overcome by taking advantage of the emission sensitization ensured by the energy transfer from coordinated organic ligands or metalloligands through their LC or MC (e.g., d-d), as well as MLCT/LMCT states.^43−45^ Sometimes, e.g., in the case of Tb^III^ complexes, the higher-lying interconfigurational d-f electronic states are the sources of efficient excitation.^46,47^ This type of electronic states can be also responsible for the emission property, which is observed for Ce^III^ complexes.^48,49^ Moreover, the lanthanide(3+) ions offer advanced multiphonon processes, e.g., up-conversion luminescence, leading to visible emission under near-infrared excitation.^50^ Therefore, lanthanide-based coordination systems are efficiently applied for the generation of color-tunable and white-light emissions.^51−53^ The challenge remains in the search for molecular platforms that can help in the effective exploration of the rich luminescence properties of lanthanide ions, ensuring good emission sensitization pathways, limiting also the interlanthanide energy transfer especially when the goal is to achieve tunable and efficient white-light emission spectrum leading to the construction of high-performance WLEDs.^54,55^ In this context, we and other groups examined various polycyanido complexes of transition metals^56,57^ as metalloligands linking lanthanide ions into diverse heterometallic d-f coordination networks, also often sensitizing the 4f-metal-centered emission.^58−87^ Polycyanidometallates were often found emissive in the visible-to-NIR ranges due to the d-d electronic transitions, e.g., [Cr^III^(CN)_6_]^3–^ and [Co^III^(CN)_6_]^3–^,^60−62^ or charge-transfer-type transitions, e.g., [Ru^II^(CN)_4_(L_NN_)]^2–^ and [Os^II^(CN)_4_(L_NN_)]^2–^ (L_NN_ = aromatic N,N′-bidentate ligands) as well as [Pt^II^(CN)_4_]^2–^ and [Au^I^(CN)_2_]^−^ (when forming the Pt–Pt or Au–Au metallophilic stacks, respectively).^59,63−69^ When their d-d or CT electronic states are lying at sufficiently high energy, the polycyanido metal complexes are used for the sensitization of lanthanide ions.^59−63,65−67,69−72^ Alternatively, some polycyanidometallates, not emissive but optically silent in the vis–NIR range, serve as transparent linkers for the d-f coordination compounds showing the functionalities related to the 4f-metal-centered emission.^55,73−81^ We and others found that lanthanide–polycyanidometallate coordination systems can reveal an extraordinary multifunctional character especially due to their attractive magnetic, electrical, and thermal expansion properties.^58,73−87^ Among them, the multifunctionality was particularly impressive when the additional physical properties, such as magnetic ordering, molecular nanomagnetism, or humidity-driven proton conductivity, were combined with photoluminescent features.^58,73−81^

In these regards, searching for the proper polycyanidometallate to achieve lanthanide-based multicolor and white-light emissions, we decided to focus on the rarely explored [Ru^II^(CN)_6_]^4–^ ions, which do not absorb in the visible range due to the strong ligand field ensured by cyanido ligands but show weak blue emission due to the d-d electronic transitions.^88^ They also form three-dimensional coordination networks with lanthanide (Ln) ions with the support of alkali metal ions.^89−91^ Thus, we focused on the Ru-CN-Ln cyanido-bridged assemblies examining the combinations with various emissive Ln^III^ centers (Ce^III^, Tb^III^, and Sm^III^) that can lead to multicolor-to-white-light emission. We report a series of K{Ln^III^(H_2_O)_*n*_[Ru^II^(CN)_6_]}·*m*H_2_O (Ln = La, Ce, Sm, Tb; Table 1) coordination networks, including the mono-lanthanide compounds (**1**–**4**), as well as the heterobi-lanthanide (Sm/Ce, Tb/Ce; **5**–**14**) and the heterotri-lanthanide (Sm/Tb/Ce; **15**) materials exhibiting the room-temperature excitation-wavelength-dependent multicolor emission and the WLE effect for the heterotri-lanthanide system (**15**), all achieved by the simultaneous exploration of d-f/f-f and d-d electronic transitions of lanthanide and transition metal ions, respectively. We present the syntheses and physicochemical characterization of this family of compounds, including detailed studies of their solid-state photoluminescent properties, completed by the magnetic studies checking the eventual single-molecule magnet behavior.

**Table 1:** List of Obtained Compounds with Corresponding Formulas^a^

compound	formula
**1**	K{La^III^(H_2_O)_3_[Ru^II^(CN)_6_]}·1.2H_2_O
**2**	K{Ce^III^(H_2_O)_3_[Ru^II^(CN)_6_]}·1.3H_2_O
**3**	K{Sm^III^(H_2_O)_2_[Ru^II^(CN)_6_]}·2.4H_2_O
**4**	K{Tb^III^(H_2_O)_2_[Ru^II^(CN)_6_]}·2.4H_2_O
**5**	K{Sm_0.47_Ce_0.53_(H_2_O)_3_[Ru(CN)_6_]}·H_2_O
**6**	K{Sm_0.81_Ce_0.19_(H_2_O)_3_[Ru(CN)_6_]}·H_2_O
**7**	K{Sm_0.88_Ce_0.12_(H_2_O)_3_[Ru(CN)_6_]}·H_2_O
**8**	K{Sm_0.99_Ce_0.01_(H_2_O)_2_[Ru(CN)_6_]}·2.1H_2_O
**9**	K{Sm_0.998_Ce_0.002_(H_2_O)_2_[Ru(CN)_6_]}·2.1H_2_O
**10**	K{Tb_0.56_Ce_0.44_(H_2_O)_3_[Ru(CN)_6_]}·1.1H_2_O
**11**	K{Tb_0.65_Ce_0.35_(H_2_O)_2_[Ru(CN)_6_]}·2.4H_2_O
**12**	K{Tb_0.93_Ce_0.07_(H_2_O)_2_[Ru(CN)_6_]}·2.4H_2_O
**13**	K{Tb_0.99_Ce_0.01_(H_2_O)_2_[Ru(CN)_6_]}·2.5H_2_O
**14**	K{Tb_0.997_Ce_0.003_(H_2_O)_2_[Ru(CN)_6_]}·2.4H_2_O
**15**	K{Sm_0.4_Tb_0.599_Ce_0.001_(H_2_O)_2_[Ru(CN)_6_]}·2.5H_2_O

a The oxidation states of metal centers are depicted for **1**–**4**. They remain identical for the other compounds, **5**–**15**.

## Experimental Section

### Starting Materials

Lanthanum(III) nitrate hexahydrate, La(NO_3_)_3_·6H_2_O (CAS: 10277-43-7), cerium(III) nitrate hexahydrate, Ce(NO_3_)_3_·6H_2_O (CAS: 10294-41-4), samarium(III) nitrate hexahydrate, Sm(NO_3_)_3_·6H_2_O (CAS: 13759-83-6), terbium(III) nitrate pentahydrate, Tb(NO_3_)_3_·5H_2_O (CAS: 57584-27-7), and potassium hexacyanidoruthenate(II), K_4_[Ru(CN)_6_]·*x*H_2_O (CAS: 339268-21-2, considered as a trihydrate) were purchased from Sigma-Aldrich.

### Synthesis and Basic Characterization

A total number of 15 coordination polymers, **1**–**15** were synthesized. The full list of their formulas is gathered in Table 1, while the details of the syntheses are presented below.

### Synthesis of **1** (LaRu d- and f-Block Metal Composition)

The 28.1 mg (0.066 mmol) of La(NO_3_)_3_·6H_2_O was dissolved in 5 mL of distilled water. As a next step, the water solution (5 mL) of K_4_[Ru(CN)_6_]·*x*H_2_O (31.2 mg, 0.066 mmol) was added. The resulting solution was left undisturbed in the dark for 1 day. Then, the crystalline powder of **1** appeared. It was collected by suction filtration, washed with distilled water, and dried in the air. The crystals suitable for the single-crystal X-ray diffraction (SC-XRD) measurement were obtained by mixing the more diluted aqueous solutions of La(NO_3_)_3_·6H_2_O (5.7 mg, 0.013 mmol; 2 mL of distilled water) and K_4_[Ru(CN)_6_]·*x*H_2_O (6.25 mg, 0.013 mmol; 2 mL of distilled water). The resulting solution was left closed in the dark for crystallization. The air-stable colorless plate crystals appeared after a few days. The composition of **1** (Table 1) was determined by an SC-XRD analysis, confronted with the thermogravimetric (Figure S2) and CHN elemental analyses. The phase purity of the bulk sample of **1** was checked by the powder X-ray diffraction (P-XRD) method, which was confronted by the P-XRD pattern simulated from the structural model obtained by the SC-XRD analysis (Figure S5). Yield: 29.3 mg, 86.9% (the powder sample). The IR spectrum of **1** (Figure S1) confirms the presence of CN^–^ ligands; cyanido stretching vibrations observed at 2075 and 2036 cm^–1^ are related to bridging cyanides of [Ru(CN)_6_]^4–^ moieties. Elem anal. calcd for K_1_La_1_Ru_1_C_6_N_6_O_4.2_H_8.4_ (**1**, *M*w = 510.8 g·mol^–1^): C, 14.1%; H, 1.7%; N, 16.5%. Found: C, 13.9%; H, 1.7%; N, 16.2%.

### Syntheses of **2** (CeRu), **3** (SmRu), and **4** (TbRu)

The synthetic procedures are analogous to those described for **1**. To obtain powder samples, the 0.066 mmol portion of the appropriate lanthanide nitrate was dissolved in 5 mL of distilled water. Then, the water solution (5 mL) of K_4_[Ru(CN)_6_]·*x*H_2_O (31.2 mg, 0.066 mmol) was added and the resulting mixture was left undisturbed in the dark for 1 day. The white crystalline powder of the respective compound was collected by suction filtration, washed with distilled water, and dried in the air. The crystals of the quality sufficient for the SC-XRD measurement were prepared by mixing the more diluted aqueous solutions of the proper lanthanide nitrate (0.013 mmol; 5 mL of distilled water for **2** and **4**, 7 mL for **3**) and K_4_[Ru(CN)_6_]·*x*H_2_O (6.25 mg, 0.013 mmol; 5 mL of distilled water for **2** and **4**, 7 mL for **3**). The resulting solutions containing the respective mixture of metal complexes were left closed in the dark for crystallization. The air-stable colorless plate crystals appeared after a few days. The compositions of **2**–**4** (Table 1) were found, combining the results of the SC-XRD analysis, TGA (Figure S2), and CHN elemental analysis. The phase purity of the bulk samples of **2–4** was checked by the P-XRD method, which was confronted by the P-XRD pattern calculated from the respective structural models obtained by the SC-XRD analysis (Figure S5). Yields (powder samples): **2**, 29.6 mg, 87.2%; **3**, 29.2 mg, 84.2%; **4**, 30.8 mg, 87.5%. IR spectra (cm^–1^, cyanido stretching vibrations, Figure S1): **2**, 2074, 2037; **3**, 2079, 2043; **4**, 2081, 2045. Elem anal. calcd for K_1_Ce_1_Ru_1_C_6_N_6_O_4.3_H_8.6_ (**2**, *M*w = 513.7 g·mol^–1^): C, 14.0%; H, 1.6%; N, 16.4%. Found: C, 14.3%; H, 1.6%; N, 16.4%. Elem anal. calcd for K_1_Sm_1_Ru_1_C_6_N_6_O_4.4_H_8.8_ (**3**, *M*w = 525.5 g·mol^–1^): C, 13.7%; H, 1.6%; N, 16.0%. Found: C, 13.8%; H, 1.6%; N, 16.0%. Elem anal. calcd for K_1_Tb_1_Ru_1_C_6_N_6_O_4.4_H_8.8_ (**4**, *M*w = 534.1 g·mol^–1^): C, 13.5%; H, 1.6%; N, 15.7%. Found: C, 13.6%; H, 1.6%; N, 15.8%.

### Syntheses of **5**–**9** (Sm_*x*_Ce_1–*x*_Ru Series)

All compounds in this series were prepared similarly using the proper mixture of two different lanthanide nitrates and K_4_[Ru(CN)_6_]·*x*H_2_O (31.2 mg, 0.066 mmol). The respective used amounts of Sm(NO_3_)_3_·6H_2_O and Ce(NO_3_)_3_·6H_2_O were as follows: 0.033 (14.8 mg) and 0.033 mmol (14.4 mg) for **5**, 0.050 (22.2 mg) and 0.016 mmol (7.2 mg) for **6**, 0.0593 (26.4 mg) and 0.0067 mmol (2.9 mg) for **7**, 0.06531 (29.0 mg) and 0.00069 mmol (0.3 mg) for **8**, and 0.065931 (29.3 mg) and 0.000069 mmol (0.03 mg) for **9**. For all compounds, the lanthanide precursors were dissolved in 5 mL of distilled water, and the 5 mL water solution of K_4_[Ru(CN)_6_] was added. The resulting solutions were left undisturbed in the dark for 1 day. This provided the white powder samples of **5**–**9**, which were collected by suction filtration, washed with distilled water, and dried in the air. The single crystals were not prepared for this series; all physical studies were performed on the air-stable powder samples primarily investigated by the powder X-ray diffraction (P-XRD) method (Figure S11). The compositions of **5**–**9** (Table 1) were determined by combining the results of P-XRD, TGA (Figure S9), CHN elemental analysis, and the SEM EDXMA microanalysis of the Ce/Sm ratio (Table S6). Yields: **5**, 25.2 mg, 74.4%; **6**, 26.5 mg, 77.7%; **7**, 24.8 mg, 72.6%; **8**, 30.2 mg, 87.3%; **9**, 29.7 mg, 86.5%. IR spectra (cm^–1^, cyanido stretching vibrations, Figure S8): **5**, 2076, 2041; **6**, 2078, 2043; **7**, 2078, 2043; **8**, 2078, 2043; **9**, 2078, 2043. Elem anal. calcd for K_1_Sm_0.47_Ce_0.53_C_6_N_6_O_4_H_8_ (**5**, *M*w = 513.3 g·mol^–1^): C, 14.0%; H, 1.6%; N, 16.4%. Found: C, 13.7%; H, 1.7%; N, 16.7%. Elem anal. calcd for K_1_Sm_0.81_Ce_0.19_C_6_N_6_O_4_H_8_ (**6**, *M*w = 516.7 g·mol^–1^): C, 13.9%; H, 1.6%; N, 16.3%. Found: C, 13.9%; H, 1.6%; N, 16.7%. Elem anal. calcd for K_1_Sm_0.88_Ce_0.12_C_6_N_6_O_4_H_8_ (**7**, *M*w = 517.5 g·mol^–1^): C, 13.9%; H, 1.6%; N, 16.2%. Found: C, 13.7%; H, 1.6%; N, 16.5%. Elem anal. calcd for K_1_Sm_0.99_Ce_0.01_C_6_N_6_O_4.1_H_8.2_ (**8**, *M*w = 520.4 g·mol^–1^): C, 13.8%; H, 1.6%; N, 16.2%. Found: C, 13.7%; H, 1.6%; N, 16.7%. Elem anal. calcd for K_1_Sm_0.998_Ce_0.002_C_6_N_6_O_4.1_H_8.2_ (**9**, *M*w = 520.5 g·mol^–1^): C, 13.8%; H, 1.6%; N, 16.2%. Found: C, 13.7%; H, 1.6%; N, 16.7%.

### Syntheses of **10**–**14** (Tb_*x*_Ce_1–*x*_Ru Series)

The compounds of this series were prepared similarly using the proper mixture of two different lanthanide nitrates and K_4_[Ru(CN)_6_]·*x*H_2_O (31.2 mg, 0.066 mmol). The respective used amounts of Tb(NO_3_)_3_·5H_2_O and Ce(NO_3_)_3_·6H_2_O were as follows: 0.033 (14.4 mg) and 0.033 mmol (14.4 mg) for **10**, 0.050 (21.6 mg) and 0.016 mmol (7.2 mg) for **11**, 0.0593 (25.8 mg) and 0.0067 mmol (2.9 mg) for **12**, 0.06531 (28.4 mg) and 0.00069 mmol (0.3 mg) for **13**, and 0.065931 (28.7 mg) and 0.000069 mmol (0.03 mg) for **14**. For all compounds, the lanthanide precursors were dissolved in 5 mL of distilled water, and the 5 mL water solution of K_4_[Ru(CN)_6_] was added. The resulting solutions were left undisturbed in the dark for 1 day. This gave the white powder samples of **10**–**14**, which were collected by suction filtration, washed with distilled water, and dried in the air. The single crystals were not prepared for this series; all physical studies were performed on the air-stable powder samples primarily studied by the P-XRD method (Figure S17). The compositions of **10**–**14** (Table 1) were determined by combining the results of P-XRD, TGA (Figure S15), CHN elemental analysis, and the SEM EDXMA microanalysis of the Ce/Tb ratio (Table S8). Yields: **10**, 24.8 mg, 71.3%; **11**, 27.7 mg, 79.5%; **12**, 22.4 mg, 63.7%; **13**, 27.0 mg, 76.6%; **14**, 27.0 mg, 76.6%. IR spectra (cm^–1^, cyanido stretching vibrations, Figure S14): **10**, 2075, 2041; **11**, 2078, 2043; **12**, 2079, 2046; **13**, 2081, 2046; **14**, 2081, 2046. Elem anal. calcd for K_1_Tb_0.56_Ce_0.44_C_6_N_6_O_4.1_H_8.2_ (**10**, *M*w = 520.8 g·mol^–1^): C, 13.8%; H, 1.6%; N, 16.1%. Found: C, 13.7%; H, 1.7%; N, 16.6%. Elem anal. calcd for K_1_Tb_0.65_Ce_0.35_C_6_N_6_O_4.4_H_8.8_ (**11**, *M*w = 527.9 g·mol^–1^): C, 13.6%; H, 1.7%; N, 15.9%. Found: C, 13.3%; H, 1.6%; N, 16.3%. Elem anal. calcd for K_1_Tb_0.93_Ce_0.07_C_6_N_6_O_4.4_H_8.8_ (**12**, *M*w = 533.2 g·mol^–1^): C, 13.5%; H, 1.7%; N, 15.8%. Found: C, 13.1%; H, 1.7%; N, 16.0%. Elem anal. calcd for K_1_Tb_0.99_Ce_0.01_C_6_N_6_O_4.5_H_9_ (**13**, *M*w = 536.1 g·mol^–1^): C, 13.4%; H, 1.7%; N, 15.7%. Found: C, 13.1%; H, 1.7%; N, 16.0%. Elem anal. calcd for K_1_Tb_0.997_Ce_0.003_C_6_N_6_O_4.4_H_8.8_ (**14**, *M*w = 534.4 g·mol^–1^): C, 13.5%; H, 1.7%; N, 15.7%. found: C, 13.4%; H, 1.6%; N, 16.2%.

### Synthesis of **15** (Sm_0.4_Tb_0.599_Ce_0.001_)

The synthetic procedure is analogous to those described for **1–14**. First, the appropriate water solution (5 mL) of three different lanthanide nitrates, including Tb(NO_3_)_3_·5H_2_O (69.2 mg, 0.16 mmol), Sm(NO_3_)_3_·6H_2_O (47.4 mg, 0.1066 mmol), and Ce(NO_3_)_3_·6H_2_O (0.12 mg, 0.0003 mmol), was prepared. Then, the water solution (5 mL) of K_4_[Ru(CN)_6_]·*x*H_2_O (124.8 mg, 0.2669 mmol) was added. The resulting mixture was left undisturbed in the dark for 1 day, providing the white polycrystalline sample of **15**, which was collected by suction filtration, washed with distilled water, and dried in the air. The single crystals were not prepared for this material, and thus all physical studies were performed on the air-stable powder sample primarily characterized by the P-XRD method (Figure S23). The composition of **15** (Table 1) was determined by combining the results of P-XRD, TGA (Figure S21), CHN elemental analysis, and the SEM EDXMA microanalysis of the Ce/Tb and Ce/Sm ratios (Table S10). Yield: 120.3 mg, 84.6%. IR spectrum (cm^–1^, cyanido stretching vibrations, Figure S20): 2082, 2074, 2045. Elem anal. calcd for K_1_Sm_0.4_Tb_0.599_Ce_0.001_C_6_N_6_O_4.5_H_9_ (**15**, *M*w = 532.8 g·mol^–1^): C, 13.5%; H, 1.7%; N, 15.8%. found: C, 13.4%; H, 1.7%; N, 15.6%.

### X-ray Diffraction Methods

For a single-crystal X-ray diffraction analysis, the crystals of **1–4** were taken directly from mother solutions, dispersed in Apiezon N grease, mounted on a Micro Mounts holder, and measured at *T* = 100 K. These measurements were performed using a Bruker D8 Quest Eco Photon50 CMOS diffractometer equipped with a Mo Kα radiation source and Triumph optics. The crystal structures were solved by an intrinsic phasing method using SHELXT-2014/5, and refined using a weighted full-matrix least-square method on *F*^2^ with SHELX-2018/3.^92^ Refinement procedures were conducted using a WinGX (ver. 2014.1) integrated software. All nonhydrogen atoms were refined anisotropically. Hydrogen atoms of water molecules were found from an electron density map; however, hydrogen atoms of partially occupied water molecules could not be found due to insufficient quality of collected data. It was also necessary to apply a series of ISOR restraints on the part of nonhydrogen atoms to ensure the convergence of the refinement procedure, maintaining the proper molecular geometries. Full details of crystal data and structure refinement are gathered in Table S1 whereas detailed structure parameters can be found in Table S2. CCDC reference numbers are 2201167, 2201168, 2201169, and 2201170 for **1–4**, respectively. Structural figures were prepared with Mercury 2021.2.0 software. Powder X-ray diffraction patterns were measured on a Bruker D8 Advance Eco powder X-ray diffractometer equipped with a Cu Kα radiation source and a capillary spinning add-on.

### Physical Techniques and Calculations

Infrared absorption spectra were measured on the selected single crystals of **1**–**15** using an FTIR microscope, Nicolet iN10 MX. The UV–vis–NIR absorption spectra were gathered using a Shimadzu UV-3600i Plus UV–vis–NIR spectrophotometer. The CHN elemental analyses were performed on an Elementar Vario Micro Cube analyzer. The 4f-metal compositions for the heterobi- and heterotri-lanthanide materials were determined using a Hitachi S-4700 scanning electron microscope (SEM) equipped with an energy-dispersive X-ray NORAN Vintage microanalysis system (EDXMA). The SEM EDXMA was used to obtain information concerning not only the metallic ratio for the obtained polycrystalline samples of heterometallic compounds but also the dispersion of the lanthanide ions within the crystals and the fragments of the selected crystals. Photoluminescence characteristics, including room-temperature solid-state emission and excitation spectra, were investigated using an FS5 spectrofluorometer (Edinburgh Instruments) equipped with a Xe (150 W) arc lamp as an excitation source and a Hamamatsu photomultiplier of the R928P type as a detector. Emission lifetime measurements were conducted on the same FS5 spectrofluorometer by an FS5 multichannel scaling method using a Xe microsecond flash lamp for **1**, **3**, and **4** (the μs-to-s lifetime range) or by an FS5 time-correlated single photon counting technique using a picosecond pulse laser diode (320 nm) for **2** (the ps-to-μs lifetime range). Absolute photoluminescence quantum yields (APLQYs) were measured by a direct excitation method using an integrating sphere module of the FS5 apparatus.^93^ Background corrections were performed within the Fluoracle software (Edinburgh Instruments). Continuous Shape Measure analysis for the eight- and nine-coordinated Ln^III^ complexes as well as six-coordinated Ru^II^ complexes in **1**–**4** was executed using the SHAPE software ver. 2.1.^94^ Calculations of photoluminescence emission lifetimes and APLQYs were performed using the Fluoracle software (Edinburgh Instruments). Measurements of magnetic properties were performed using a Quantum Design MPMS-3 Evercool magnetometer. For these studies, the powder samples of **2**–**4** were covered with paraffin oil and placed in the polycarbonate capsule with cotton wool. Diamagnetic corrections from the sample and the sample holder were taken into account.

## Results and Discussion

### Synthesis and Structural Studies of Mono-Lanthanide Compounds, **1**–**4**

**Figure 1:**
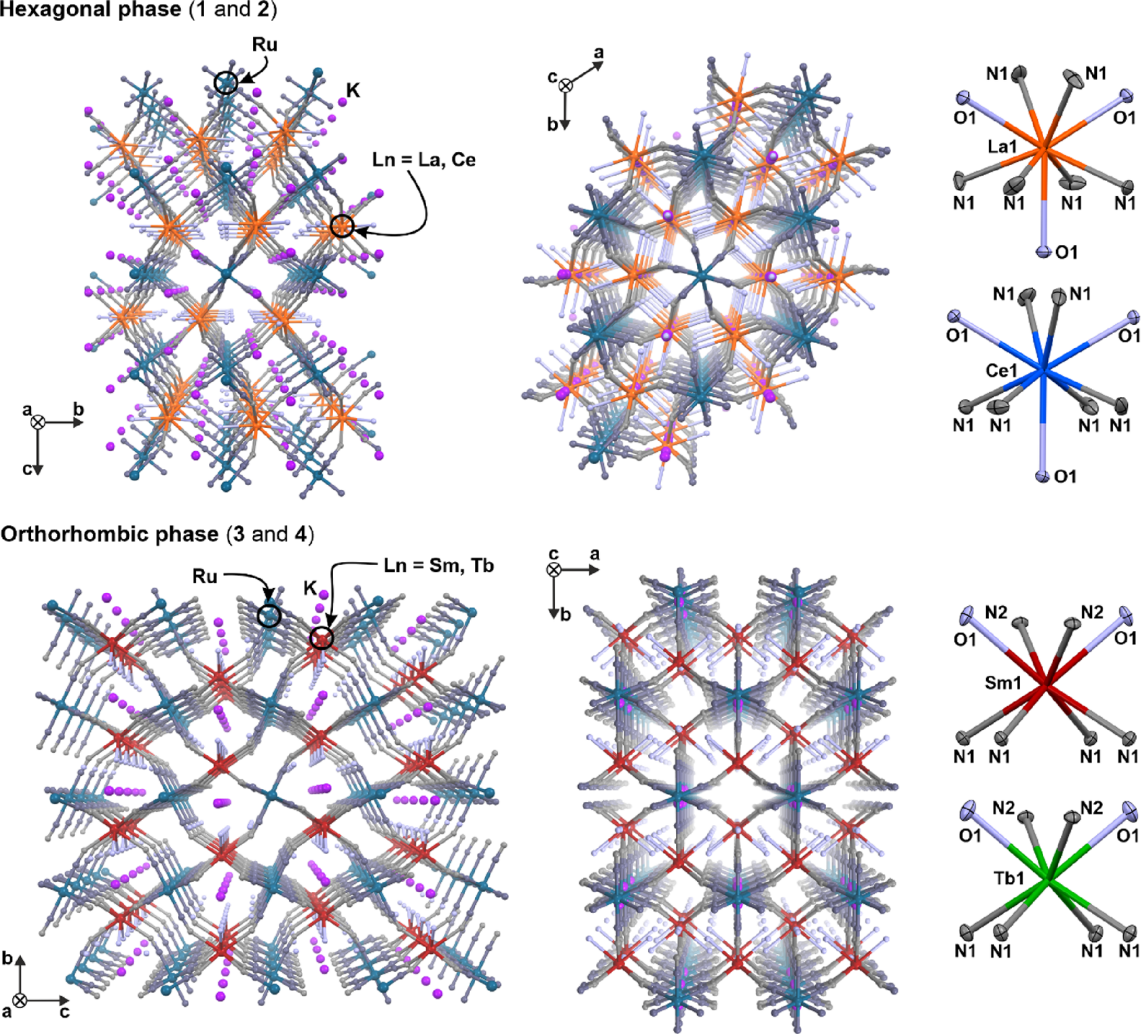
Crystal structures of **1** and **2** (hexagonal phase, top) as well as **3** and **4** (orthorhombic phase, bottom), illustrated by the views along *a* (left) and *c* (center) crystallographic axes, and the detailed views of the first coordination spheres of lanthanide ions (right). The hydrogen atoms as well as noncoordinated water molecules are omitted for clarity.

Colorless crystals of **1–4** were obtained by mixing the equimolar water solutions of the potassium hexacyanidoruthenate(II) and the nitrate salt of the appropriate lanthanide(3+) ion, namely, La^3+^, Ce^3+^, Sm^3+^, or Tb^3+^, respectively. The obtained materials were characterized by infrared spectroscopy, which revealed the presence of bridging cyanido ligands of [Ru(CN)_6_]^4–^ anions (Figure S1). Further, the single-crystal X-ray diffraction (SC-XRD) experiments indicated that compounds **1** and **2**, containing larger-size lanthanide ions, crystalize in the *P*6_3_/m space group of a hexagonal crystal system while compounds **3** and **4**, containing smaller-size lanthanide ions, crystalize in the *C*mcm space group of an orthorhombic crystal system (Figure 1 and Figure S3, Tables S1–S4). The SC-XRD analyses, supported by the CHN elemental analyses and thermogravimetry (Figure S2), provide information on the exact compositions of **1**–**4**, which include the {KLn[Ru(CN)_6_]} part (Ln = La, Ce, Sm, Tb), identical in the whole family, and the variable number of water molecules (Table 1, see Experimental Section for details).

Compounds **1** and **2** are three-dimensional (3D) coordination polymers composed of negatively charged heterometallic cyanido-bridged skeleton incorporating {Ru–CN–Ln} linkages, crystallizing together with K^+^ counter-ions and interstitial water solvent molecules (Figure 1, top). In this framework, octahedral [Ru(CN)_6_]^4–^ complexes link six different lanthanide(III) sites, using all of the accessible CN^–^ ligands as molecular bridges (Tables S2 and S3) while nine-coordinated [Ln^III^(NC)_6_(H_2_O)_3_]^3–^ entities consist of six CN^–^ ligands, originating from neighboring Ru^II^ complexes, and three coordinated water molecules located within a single plane of the resulting polyhedron (Figure 1). The geometry of Ln^III^ complexes can be described as a spherical tricapped trigonal prism (Table S4). The negative charge of the coordination polymer is compensated by the K^+^ ions located in the free space of the 3D cyanido-bridged framework. The K^+^ cations are located with 0.5 occupancy, which fulfills the requirements of the neutral charge of the overall crystal structure. The remaining free space of the structure is occupied by water molecules of crystallization. Compounds **3** and **4** are similar 3D coordination polymers based on the negatively charged cyanido-bridged skeleton involving Ru^II^ and Ln^III^ centers, counter-balanced by the K^+^ ions (Figure 1, bottom); however, it crystallizes in the lower-symmetry *C*mcm space group. Identically as in **1** and **2**, the embedded [Ru(CN)_6_]^4–^ complexes in **3** and **4** are octahedral and all of their CN^–^ ligands are bridging to the adjacent Ln^III^ centers (Tables S2 and S3). The most important structural difference between the two types of structures, for **1**–**2** and **3**–**4**, is the coordination mode for lanthanide ions. In **3** and **4**, the [Ln^III^(NC)_6_(H_2_O)_2_]^3–^-type complexes of Sm^III^ and Tb^III^ are eight-coordinated as only two water molecules lie in the first coordination sphere. Their geometry is close to a square antiprism (Table S4). The negative charge of the coordination framework is counter-balanced by the K^+^ ions; though in **3** and **4**, they exhibit the full occupation at a single crystallographic position. These cations, together with the water molecules of crystallization, are located in the structural channels lying along the *a* crystallographic axis. The validity of the obtained structural models for the bulk samples of **1**–**4**, used in further optical studies, was confirmed by the powder X-ray diffraction (P-XRD) experiments (Figure S5). These studies also support the phase purity of the prepared materials.

Compounds **1**–**4** are stable in the air but gradually lose incorporated water molecules upon the exposition to the flow of nitrogen. This process is facilitated by heating even slightly above room temperature as suggested by the TG curves (Figure S2). Upon heating the samples of **1**–**4** in the nitrogen gas environment, first, the water molecules of crystallization are removed, which occurs in the 20–80 °C ranges. Further heating results in the removal of water molecules of crystallization, which lead to the fully dehydrated phases after reaching the ca. 130–150 °C region.

### Photoluminescence of Mono-Lanthanide Compounds, **1**–**4**

Compounds **1–4** were characterized by the solid-state UV–vis–NIR absorption spectroscopy in the 220–1000 nm range (Figure S4). For all compounds, the significant light absorption appears only in the UV range below ca. 320 nm, leaving efficient optical transparency for the vis-to-NIR regions. The UV-positioned absorption bands can be mainly assigned to the d-d (especially in the 250–320 nm range) and charge transfer (mainly below 250 nm) electronic transitions of [Ru^II^(CN)_6_]^4–^.^90,95^ In **1**, no other absorption peaks appear due to the close-shell character of La^III^ centers. In **2**, the additional absorption shoulder in the 300–320 nm region is observed; it can be assigned to the f-d electronic transitions.^48,49,96^ In **3**, there are no extra absorption peaks above 300 nm; however, the deeper UV absorption bands are relatively better structured. In **4**, the absorption spectrum is rather broad and the conceivable additional high-energy peak related to the f-d electronic transition cannot be distinguished from those assigned to the Ru^II^ complexes.^46,47^ The sharp absorption peaks of the f-f electronic transitions, expected for the Sm^III^ and Tb^III^ centers, are not observed, probably due to their very weak relative intensity.^39,40^

**Figure 2:**
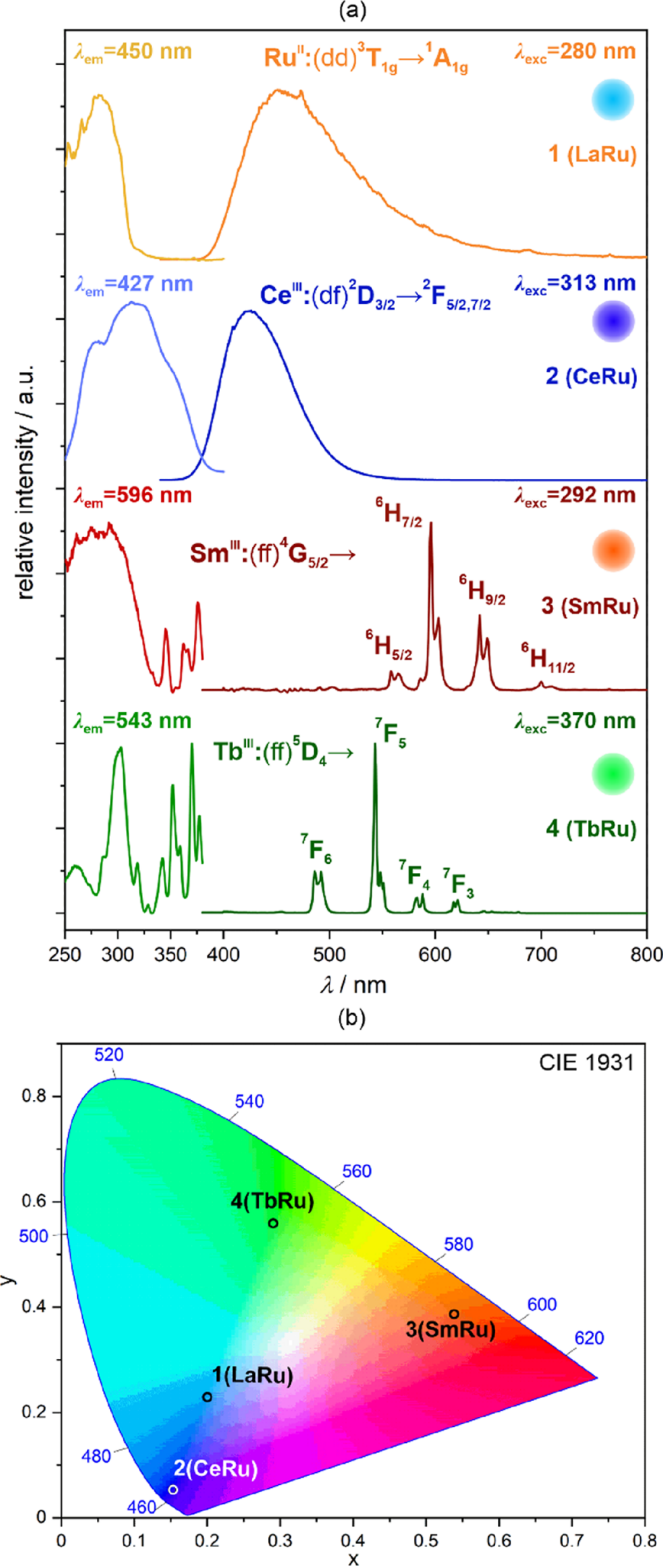
Solid-state photoluminescent characteristics of **1** (**LaRu** d- and f-block metals composition, see Table 1), **2** (**CeRu**), **3** (**SmRu**), and **4** (**TbRu**), including excitation and emission spectra at the indicated wavelength conditions (a) and the resulting emission colors presented on the CIE 1931 chromaticity diagram (b, see Table S11 for the detailed parameters). The emission colors are also illustrated in the right side of each emission pattern in (a). The electronic transitions responsible for the emission peaks are indicated. The spectra were gathered at room temperature for **2**–**4**, while the spectra for **1** were collected at 77 K.

As compounds **1**–**4** exhibit strong UV but very weak visible light absorption, we examined their photoluminescent properties (Figure 2, Figures S6 and S7). Under the UV light excitation, **1** exhibits blue emission represented by the broad peak with a maximum positioned at 450 nm. It can be ascribed to the d-d electronic transition, namely, ^3^T_1g_ → ^1^A_1g_, within Ru^II^ centers. Such emission is characteristic of the transition metal ions of the d^6^ valence electron configuration embedded in the strong ligand field.^60,61,88^ This phosphorescence is weak and not reliably detectable at room temperature, and thus it was gathered at the decreased temperature of 77 K (Figure 2a). The Ru^II^-based emission in **1** is of a sky-blue color as illustrated by the CIE 1931 chromaticity parameters of *x* = 0.20 and *y* = 0.23 (Figure 2b, Table S11). The excitation spectrum gathered in the 250–400 nm range consists of a broad complex band with the maximum at 280 nm that can be mainly assigned to the spin-allowed ^1^A_1g_ → ^1^T_1g_ d-d electronic transition, presumably overlapping with the weaker, spin-forbidden transition to the ^3^T_2g_ state. The possible charge transfer bands are expected to lie below 250 nm.^88,95^ On the other hand, the spin-forbidden ^1^A_1g_ → ^3^T_1g_ transition, which is responsible for the observed emission, is represented in the excitation spectrum by the very weak tail ranging from 300 to 340 nm. The emission lifetime in **1** reaches almost 1 ms (995.8(3) μs, Figure S6a) confirming the phosphorescent character of the observed luminescence. This value is ca. 30 times smaller than the long emission lifetime (31 ms) reported for the analogous electronic transition in the K_4_[Ru^II^(CN)_6_] salt.^88^ Such the shortening of emission lifetime is related to the heavy atom effect occurring upon the change from the salt containing lighter K^+^ ions to the coordination framework containing heavier La^3+^ ions with the enhanced spin–orbit coupling. Similar behavior was reported for the [Co^III^(CN)_6_]^3–^ ions revealing analogous phosphorescence in the K-based salt and the lanthanide-based coordination network.^60^

The emission of a similar blue color, but of a deeper hue (*x* = 0.15 and *y* = 0.05 of the CIE 1931 chromaticity parameters, Figure 2b) and a much better intensity, was detected for **2**. At room temperature (Figure 2a), the emission band of **2**, centered at 427 nm, is also broad as found in **1**; however, the respective excitation spectrum in **2** is very different to those observed for **1**, as the main maximum is positioned at 313 nm and accompanied by two shoulders toward both higher energy up to ca. 260 nm as well as lower energy up to ca. 380 nm. These observations indicate that the blue emission in **2** can be assigned to the d-f electronic transitions, namely, ^2^D_3/2_ → ^2^F_5/2,7/2_, within Ce^III^ centers rather than to the d-d transitions of Ru^II^ complexes.^48,49,96−98^ Such an interpretation is supported by the very short emission lifetime, reaching only the value of 19.8(1) ns at room temperature (Figure S6b), which is characteristic of the fluorescence-like d-f emission of Ce^3+^ ions. In this context, the excitation bands can be mainly assigned to the direct excitation of Ce^III^ centers through their spin-allowed transitions from the ^2^F_5/2_ ground multiplet to the ^2^D_3/2_ electronic states. The contribution of the Ru^II^-to-Ce^III^ energy transfer, represented by the excitation below 300 nm that corresponds to the excitation features in **1**, can be also considered as the Ru^II^-based emission is not observed in **2**. Nevertheless, the resulting emission quantum yield for **2** is high, reaching 59(5)% for the 313 nm excitation at room temperature, which is also typical for the Ce^III^-based luminophores.^48,49^ The Ce^III^-centered emission often consists of two distinguishable bands. This behavior was not found for **2** at room temperature; however, at the decreased temperature (77 K) the emission pattern is composed of two bands corresponding to the ^2^D_3/2_ → ^2^F_5/2_ and ^2^D_3/2_ → ^2^F_7/2_ electronic transitions (Figure S7). The cooling process also induces the overall redshift of the emission, which results in the emission pattern even closer to those observed in **1**; however, the emission lifetime remains very short (Figure S6c), and thus the luminescence in **2** is due to the Ce^III^-centered d-f electronic transitions in the whole *T*-range.

Compound **3** exhibits room-temperature UV-light-induced emission consisting of a series of sharp emission peaks with the main maxima at 558, 596, 642, and 700 nm (Figure 2a). They can be ascribed to the characteristic f-f electronic transitions of Sm^III^ centers, namely, from the ^4^G_5/2_ emissive level to the ^6^H_5/2_, ^6^H_7/2_, ^6^H_9/2_, and ^6^H_11/2_ states.^40,99^ The two strongest peaks appear in the 590–650 nm range, which results in the overall orangish red emission color depicted by the CIE 1931 chromaticity parameters of *x* = 0.54 and *y* = 0.39 (Figure 2b). The excitation spectrum for **3** is dominated by the broad band ranging from deep UV to 320 nm with the maximum at ca. 292 nm, which is accompanied by a series of much weaker sharp peaks in the 340–400 nm region of the spectrum. The latter can be assigned to the direct excitation through the higher-lying f-f electronic states of Sm^III^.^40,99^ On the other hand, the more efficient excitation represented by the broad band below 320 nm can be ascribed to the [Ru^II^(CN)_6_]^4–^ complexes having the d-d electronic transition in this range (as seen for **1**, Figure 2a) as the Sm^III^ complexes do not exhibit such broad absorption bands. This suggests the presence of the Ru^II^-to-Sm^III^ metal-to-metal energy transfer process, which is also supported by the disappearance of the Ru^II^-centered emission; however, the Sm^III^ emission sensitization through Ru^II^ electronic states is rather limited as the determined emission quantum yield reaches only 0.6(1)% at room temperature. The low quantum yield value can be also partially explained by the emission quenching by the vibrational states of water molecules incorporated in the structure of **3**, which often affects the lanthanide luminescence. This contribution to the quenching was confirmed by the preparation of compound **3** in the deuterated water, which results in the almost twofold increase of the related emission quantum yield to 1.1(1)%. The emission decay profile for **3** was analyzed using the double-exponential function, suggesting the existence of at least two different emission deactivation pathways; however, the process represented by the emission lifetime of 11.6(2) μs dominates (Figure S6d). This value is relatively short but typical for Sm^III^ compounds showing usually a rather weak emission with the microsecond-type emission lifetime.^40,99,100^ Similarly to **3**, compound **4** also exhibits the emission pattern composed of sharp peaks, which are characteristic for f-f electronic transitions (Figure 2a). Under the UV light excitation, the emission peaks appear at 486, 543 (the main one), 588, and 621 nm, and they can be assigned to the electronic transitions from the emissive ^5^D_4_ level of Tb^III^ centers to the lower-lying ^7^F_6_, ^7^F_5_, ^7^F_4_, and ^7^F_3_ states, respectively. The dominance of the 543 nm peak results in the overall green emission depicted by the CIE 1931 chromaticity parameters of *x* = 0.29 and *y* = 0.56 (Figure 2b). The excitation spectrum contains a series of sharp peaks in the 320–400 nm range, which are related to the f-f electronic transitions and thus represent the direct excitation of Tb^III^ centers.^101^ At lower wavelengths, two broader bands at 260 and 300 nm are observed. The latter is relatively strong and could be ascribed mainly to the interconfigurational d-f electronic transition, often detectable in this region for the Tb^III^ complexes.^47,102^ The 260 nm band with the shoulder ranging to lower energies is probably related to the energy transfer from the [Ru(CN)_6_]^4–^ units, which do not reveal their broadband emission in **4**. Therefore, it seems that the sensitization of the Tb^III^ emission by cyanido Ru^II^ complexes is moderate, and the d-f excitation is more efficient; however, it is also partially quenched as the direct f-f excitation remains at the same efficiency level. All these together result in the emission quantum yield of 5.4(5)% and the emission lifetime of 362(1) μs (Figure S6e), which lies in the range often found for the coordination systems showing the relatively good emission property of Tb^III^ complexes at room temperature.^40,47,55^

### Synthesis and Structural Studies of Heterobi-Lanthanide Sm/Ce Compounds, **5**–**9**

**Figure 3:**
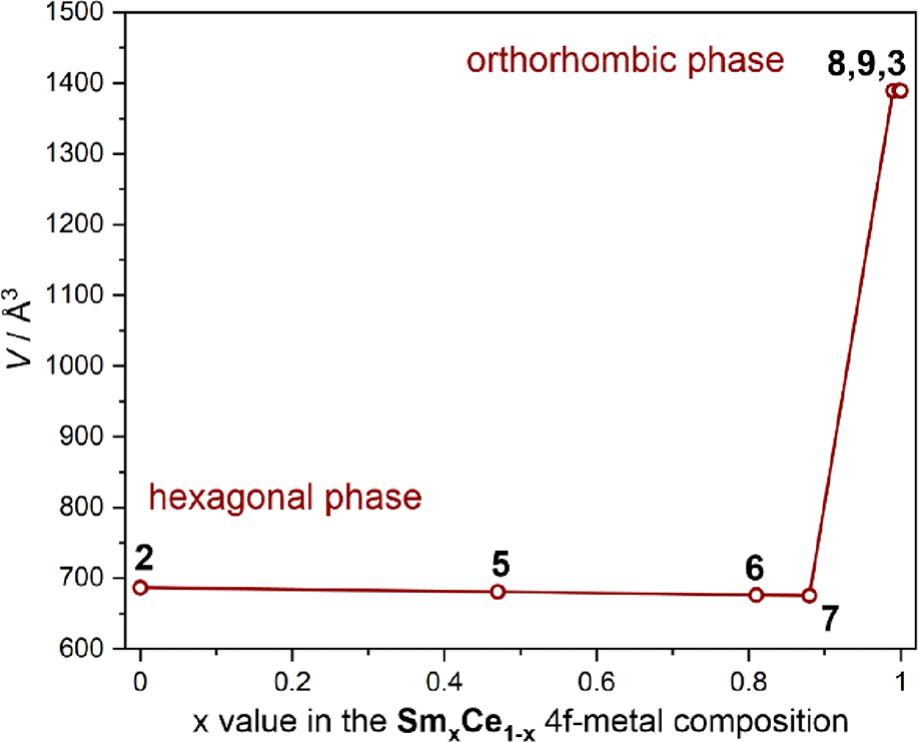
Relation between the unit cell volume and the 4f-metal composition within the series of **2**, **5**–**9**, and **3**. The assignment of the unit cell volume ranges to two possible crystalline phases, hexagonal and orthorhombic (Figure 1), is indicated. The lattice constants determined for all compounds from this series are shown in Table S5. The solid line is only to guide the eye.

Compounds **2**–**4** are the sources of room-temperature blue, red, and green emissions, respectively (Figure 2), and thus the mixed-lanthanide analogs of a modulated 4f-metal composition were expected to become the tool for efficient multicolor and white-light emissions. To identify this potential, in the first step, the series of heterobi-lanthanide Sm/Ce compounds, **5**–**9**, were prepared and characterized (see Experimental Section for details). The molar ratios of Ce^3+^ and Sm^3+^ ions used during the syntheses follow the series of 1:1, 1:3, 1:10, 1:100, and 1:1000 for **5**, **6**, **7**, **8**, and **9**, respectively, and thus the increasing amount of Sm^III^ complexes, which are much weaker luminescent than the Ce^III^ ones, was explored. Compounds **5**–**9** were initially investigated using IR and UV–vis–NIR spectroscopies (Figures S8 and S10), revealing very similar features to those found for **2** and **3**. However, the mono-lanthanide materials of **2** and **3** crystallize in two different crystal systems with the distinguishable coordination environment of 4f metal ions (Figure 1); therefore, the mixed-lanthanide systems could adopt one of these phases depending on the Ce/Sm ratio, which was examined the P-XRD technique (Figure 3 and Figure S11, Table S5). Comparison of the powder patterns and the further unit cell determination reveal that compounds **5**–**7** with the Ce/Sm ratio up to ca. 1:10 crystallize in the hexagonal phase, the same as mono-lanthanide Ce^III^-containing compound **2** (Figure 3). The larger excess of Sm^III^ centers in **8** and **9** results in the orthorhombic phase, analogous to that found for **3**. In the P-XRD patterns of the whole series **5**–**9**, there are no additional peaks except those corresponding to the hexagonal or the orthorhombic phase, and mixtures of the peaks from these two phases were also not observed. This indicates the phase purity of **5**–**9** and their perfect isostructurality with **2** (**5**–**7**) or **3** (**8**–**9**). Thus, similar to **2** and **3**, their structures consist of a three-dimensional coordination polymer based on lanthanide complexes bridged by [Ru^II^(CN)_6_]^4–^ complexes (Figure 1). A single crystallographic lanthanide position is observed, and thus the Ce^III^ and Sm^III^ centers are situated at the same position with partial occupancies. Their random distribution within the crystals is confirmed by the SEM EDXMA measurements performed for a few crystals of **5**–**9** (Figure S12 and Table S6). The related microanalyses together with the CHN elemental analyses and TG studies (Figure S9) provide the exact compositions of compounds **5**–**9**, including the precise information on the Ce/Sm ratio in the respective material (Table 1). As depicted by TG curves (Figure S9), the crystalline samples of **5**–**9** are stable in the air; however, upon heating, they gradually lose water molecules in a similar manner as described for **1**–**4** (see above and compare Figures S2 and S9).

### Photoluminescence of Heterobi-Lanthanide Sm/Ce Compounds, **5**–**9**

**Figure 4:**
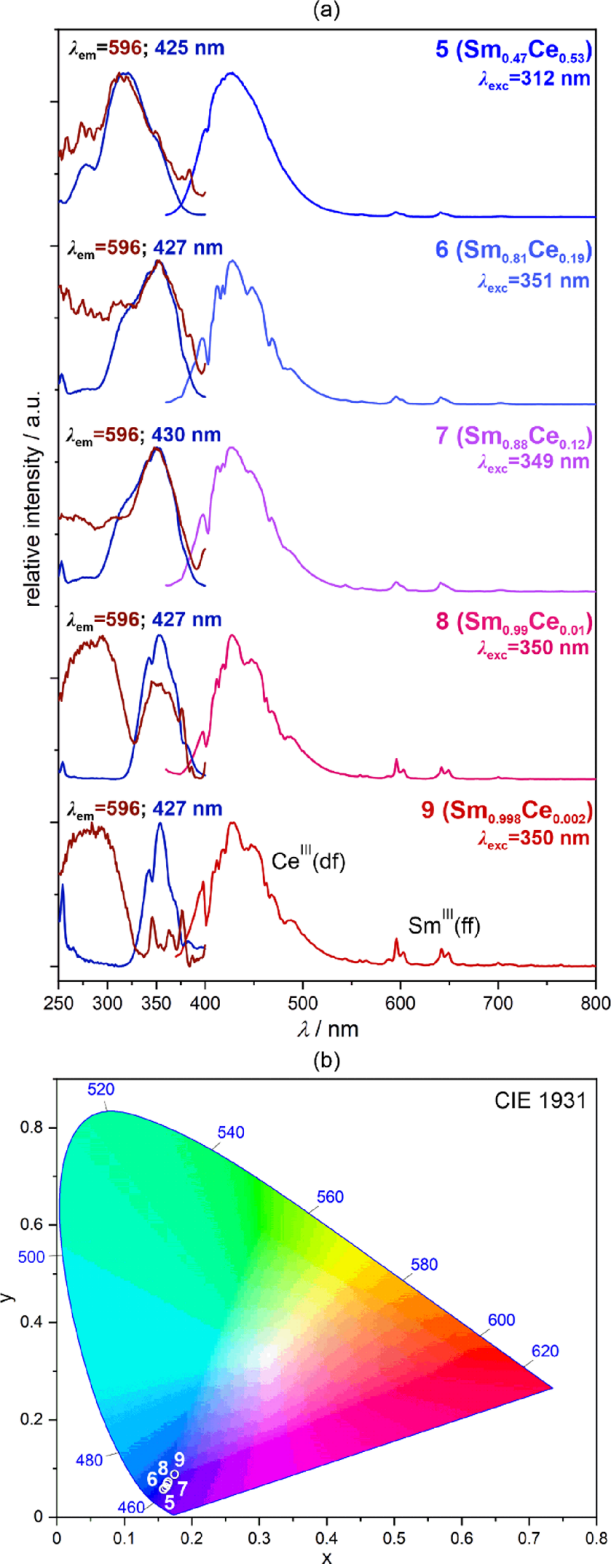
Solid-state room-temperature luminescent characteristics of **5**–**9**, including excitation and emission spectra at the indicated wavelength conditions (a) and the resulting emission colors depicted on the chromaticity diagram (b). The 4f-metal compositions are shown next to the labels of the compounds (see Table 1). The emission spectra were gathered for the excitation giving the strongest emission. The groups of electronic transitions responsible for the emission peaks are indicated, while the detailed assignment is given for each lanthanide ion in Figure 2.

**Figure 5:**
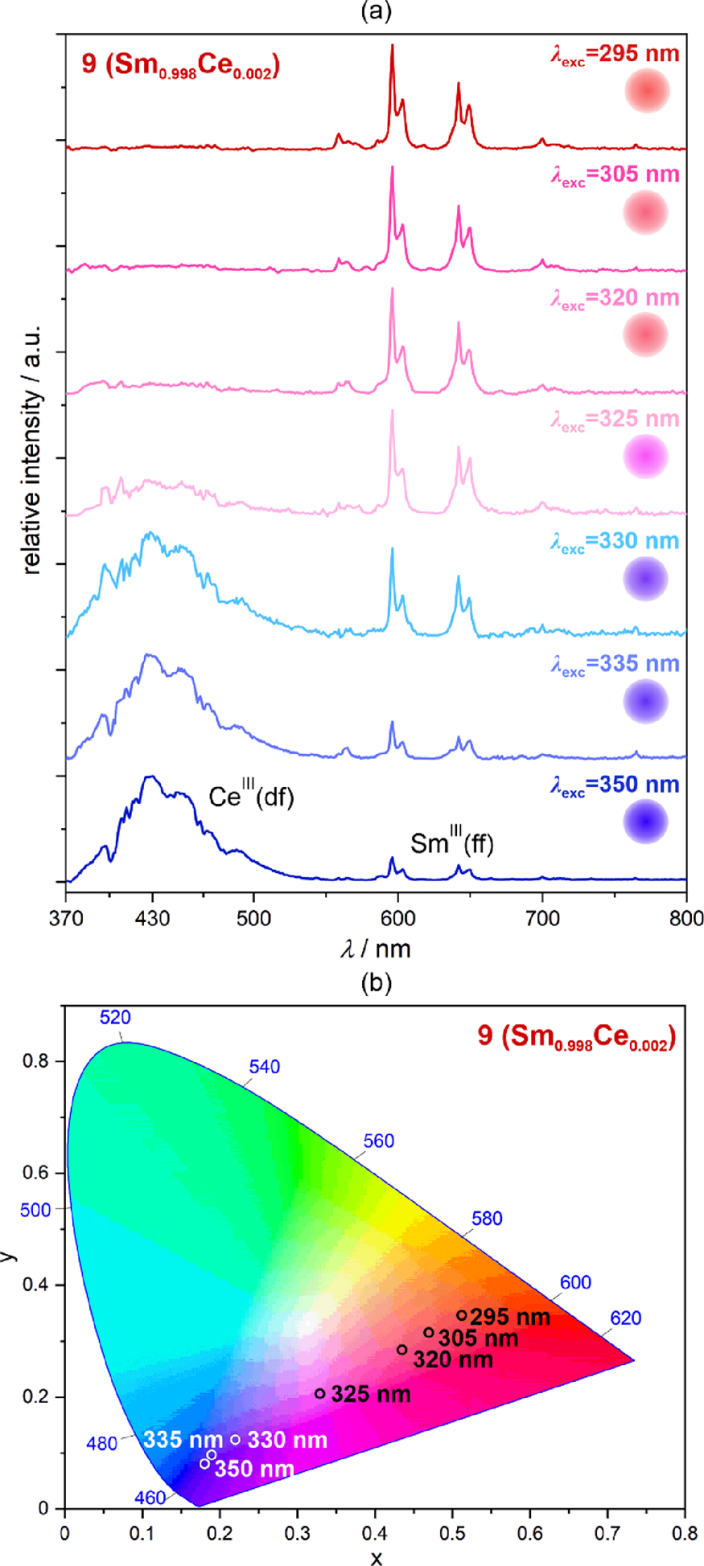
Room-temperature excitation-wavelength-variable emission spectra of **9** (Sm_0.998_Ce_0.002_ 4f-metal composition) (a) and the resulting emission colors shown on the CIE 1931 chromaticity diagram (b). The emission colors are also illustrated on the right side of each emission pattern in (a). The groups of electronic transitions responsible for the emission peaks are indicated in (a), while the detailed assignment is given for each lanthanide ion in Figure 2.

The 4f-metal compositions of **5**–**9** were selected to observe the tuning of overall emission thanks to the modulated ratio between weak luminescence of Sm^III^ complexes and very strong emission of Ce^III^ centers (see Figure 2 and the related subchapter). Therefore, for the whole series of **5**–**9**, both room-temperature emission as well as excitation spectra were gathered (Figures 4 and 5 and Figure S13). Under all possible excitation from the UV range, compounds **5**, **6**, and **7** exhibit the dominant broadband blue emission related to the d-f electronic transitions of Ce^III^ centers (Figure 4), whereas the sharp emission peaks of the red Sm^III^-centered emission are very weak. The respective excitation spectra for the monitored Ce^III^- emission are similar to those for the Sm^III^-based emission, further suggesting that these materials exhibit dominant blue emission of the Ce^III^ origin independently of the excitation wavelength. The excitation bands are broad, and they are strongest in the 280–380 and 300–400 nm range for **5** and **6**–**7**, respectively. Thus, they can be mainly assigned to the d-f Ce^III^-centered electronic transitions with the additional contribution from the d-d Ru^II^-centered transitions below 310 nm. No sharp peaks assignable to the Sm^III^ centers were detected, suggesting that the weak, yet noticeable, emission for this metal ion is realized by the energy transfer processes, from Ru^II^ centers (at deeper UV) and partially also from Ce^III^ centers (above 310 nm). As the result, the Ce^III^-based emission is significantly weakened upon the addition of Sm^3+^ ions to the framework.

Moreover, for **6** and **7** (further also for **8** and **9**) exhibiting the largest amounts of Sm^III^ centers, within the broad Ce^III^-centered emission band, one can notice the sharp negative peaks. They can be ascribed to the reabsorption effect related to the presence of Sm^III^ f-f electronic transitions in this region, e.g., the ^6^H_5/2_ → ^6^F_7/2_ transition at ca. 405 nm or the ^6^H_5/2_ → ^4^G_9/2_ transition at 445 nm.^40,99,100^ Thus, both the partial interlanthanide energy transfer as well as the reabsorption effect appear to govern the emission signals in the Sm/Ce compound; however, the Ce^III^-based emission remains dominant even for the small Ce/Sm ratio in **7**. The latter trend changes upon the further decrease of the Ce/Sm ratio in **8** and **9** as represented by the relative enhancement of the excitation bands for the monitored Sm^III^-based emission in the deeper UV region (typical for the Ru^II^-to-Sm^III^ energy transfer as found in **3**) in comparison to the excitation bands in the 300–400 nm of the Ce^III^ origin. Such modulation can be assigned to the extremely small amounts of Ce^3+^ ions, which less influence the sensitization of the Sm^III^ emission by the Ru^II^ complexes. As the result, even for the 350 nm excitation, which is more suitable for Ce^III^ and produces its dominant blue emission, the sharp emission peaks from Sm^III^ electronic transitions are more noticeable. The large excess of Sm^3+^ ions in **8** and **9** opens the route to the excitation-wavelength-dependent emission property (Figure 5 and Figure S13). As was shown for **9**, by playing with the excitation wavelength, it is possible to generate the broad emission color tuning from the blue emission of the Ce^III^ origin for the 350 nm excitation to the orangish-red emission of the purely Sm^III^ origin for the 295 nm excitation, whereas the intermediate blue-to-pink-to-red emission colors can be achieved by the excitation wavelengths from 350 to 295 nm, modulating the ratio between the Ce^III^ and Sm^III^ components (Figure 5). This tunable multicolor emission is represented by the almost linear variation from two corners of the CIE 1931 chromaticity diagram (Figure 5b, Table S11).

### Synthesis and Structural Studies of Heterobi-Lanthanide Tb/Ce Compounds, **10**–**14**

**Figure 6:**
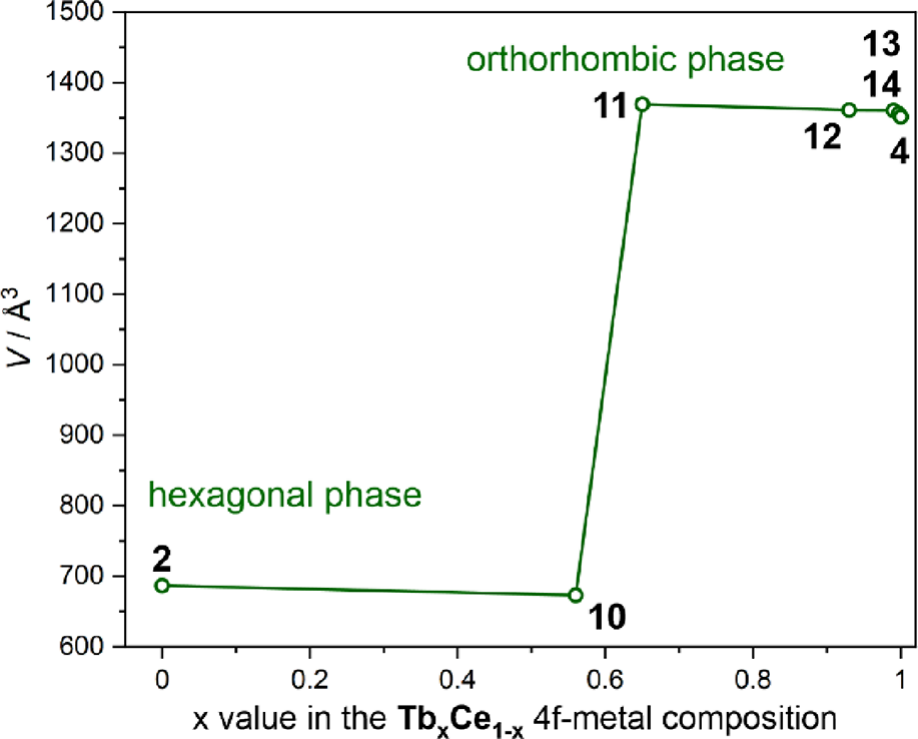
Relation between the unit cell volume and the 4f-metal composition within the series of **2**, **10**–**14**, and **4** compounds. The assignment of the unit cell volume ranges to two possible crystalline phases, hexagonal and orthorhombic (Figure 1), is indicated. The lattice constants determined for all compounds are gathered in Table S7. The solid line is only to guide the eye.

Following the emission color tuning in the Sm/Ce analogs of **5**–**9** (see above), we have prepared and characterized the series of heterobi-lanthanide Tb/Ce materials, **10**–**14** (see Experimental Section for synthetic details). Analogously to **5**–**9**, the molar ratios of Ce^3+^ to Tb^3+^ ions used in the syntheses of **10**–**14** follow the series of 1:1, 1:3, 1:10, 1:100, and 1:1000, respectively. The increasing excess of Tb^3+^ ions was explored as the Tb^III^ complexes are much weaker luminescent in the investigated framework than the Ce^III^ complexes (see the subchapter on mono-lanthanide compounds for details). The obtained heterobi-lanthanide systems of **10**–**14** exhibit similar spectroscopic features in the IR and UV–vis–NIR ranges to those found for their mono-lanthanide analogs of **2** (**CeRu**) and **4** (**TbRu**) (Figures S14 and S16). As **2** and **4** reveal distinguishable crystal structures, including lanthanide coordination environments (Figure 1), the P-XRD method was employed to determine the structures of the heterobi-lanthanide systems (Figure 6 and Figure S17, Table S7). Among them, only compound **10** crystallizes in the hexagonal phase, as the Ce^III^-based **2**, while other materials with larger amounts of Tb^3+^ ions grow in the orthorhombic phase, analogous to the pure **TbRu** framework **4**. The P-XRD patterns of **10**–**14** perfectly match the referential patterns of **2** or **4** without the sign of the phase mixtures or impurities (Figure S17). This indicates that the structures of **10**–**14** consist of the 3D cyanido-bridged networks based on lanthanide ions linked by [Ru^II^(CN)_6_]^4–^ metalloligands (Figure 1). The presence of a single crystallographic position in the structure suggests that the Ce^III^ and Tb^III^ centers occupied the same position with partial occupancies. They are randomly distributed within the crystals, without the aggregate effects, as indicated by the SEM EDXMA measurements performed for the crystals of **10**–**14** (Figure S18, Table S8). The related results of EDXMA, supported by the CHN analyses and the TG studies (Figure S15), were used to reliably determine the exact compositions of **10**–**14**, including the critical Tb/Ce metal ratios (Table 1). As shown by TG curves (Figure S15), the crystalline samples of **10**–**14** are stable in the air; however, upon heating, they gradually lose water molecules in a similar way as observed for **1**–**4** and **5–9** (see above and compare Figures S2, S9, and S15).

### Photoluminescence of Heterobi-Lanthanide Tb/Ce Compounds, 10–14

**Figure 7:**
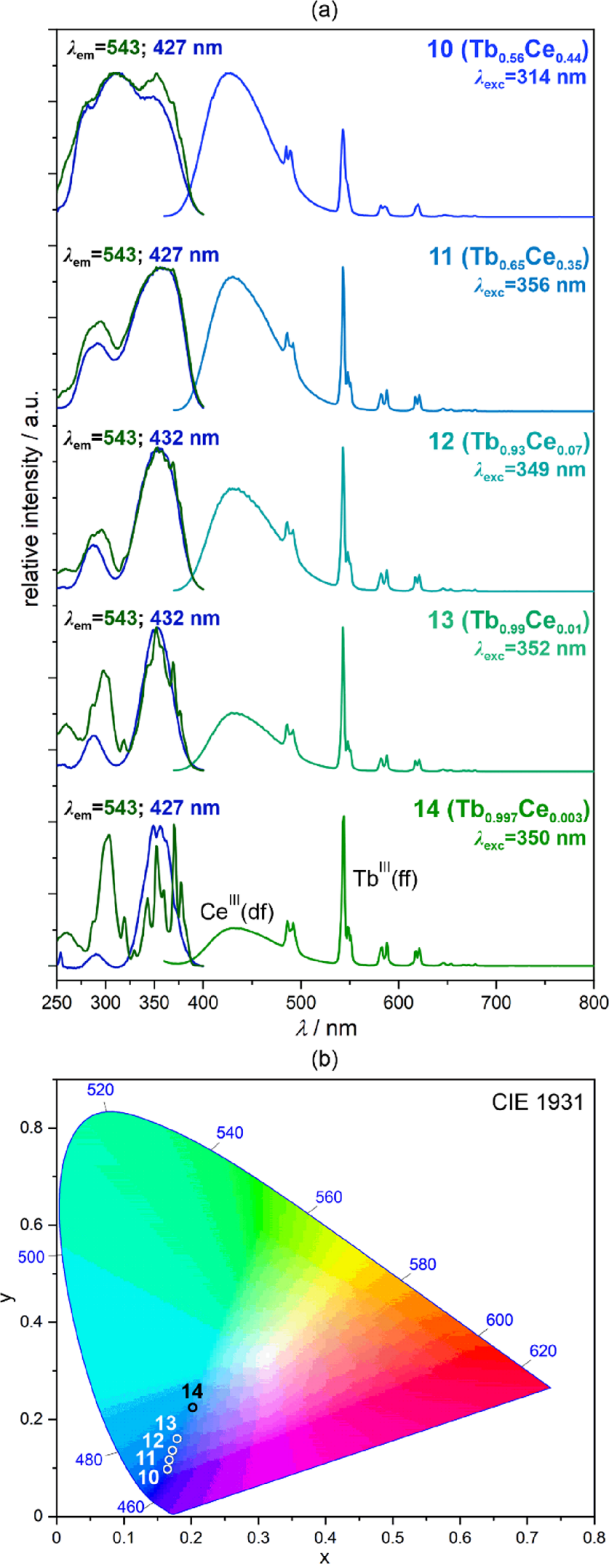
Solid-state room-temperature luminescent characteristics of **10**–**14**, including excitation and emission spectra at the indicated wavelengths (a) and the resulting emission colors depicted on the chromaticity diagram (b). The 4f-metal compositions are shown next to the labels of the compounds (see Table 1). The emission spectra were gathered for the excitation giving the strongest emission. The groups of electronic transitions responsible for the emission peaks are indicated, while the detailed assignment is given for each lanthanide ion in Figure 2.

**Figure 8:**
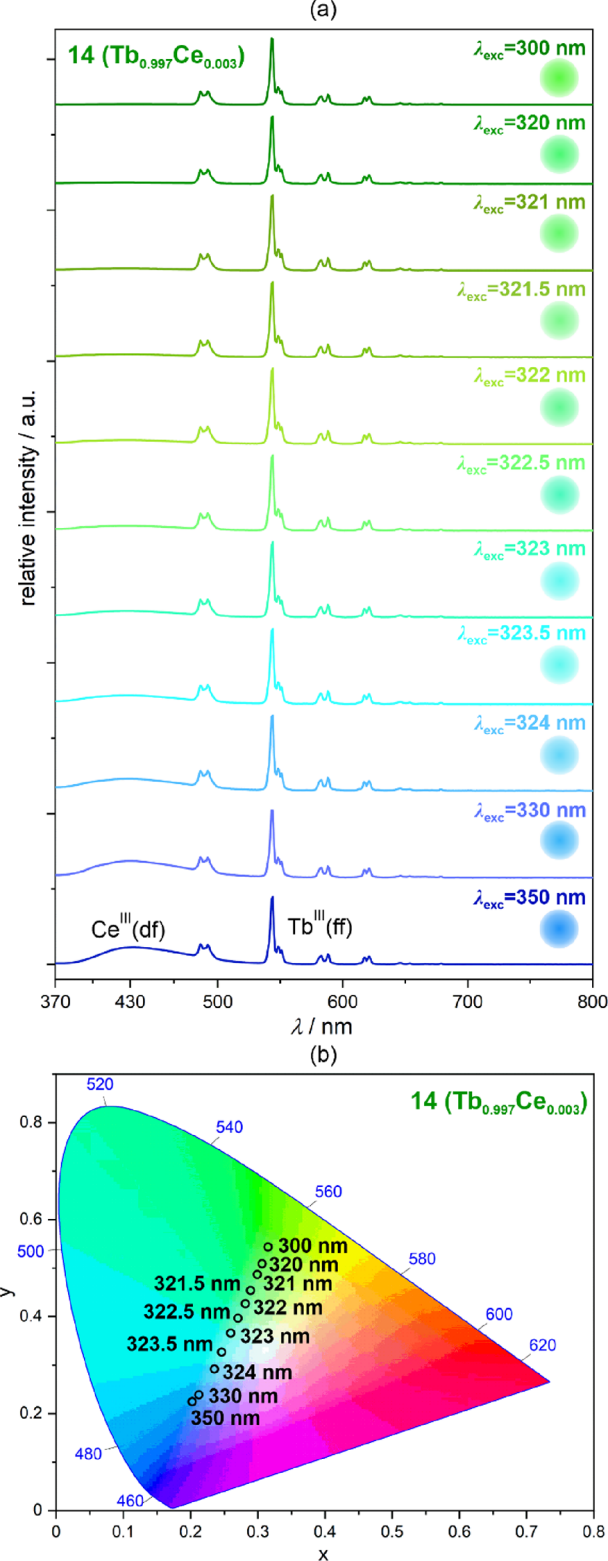
Room-temperature excitation-wavelength-variable emission spectra of **14** (Tb_0.997_Ce_0.003_ 4f-metal composition) (a) and the resulting emission colors shown on the CIE 1931 chromaticity diagram (b). The emission colors are also illustrated on the right side of each emission pattern in (a). The groups of electronic transitions responsible for the emission peaks are indicated in (a), while the detailed assignment is given for each lanthanide ion in Figure 2.

The Ce-to-Tb metal ratios in **10**–**14** were optimized to generate the tuning of overall emission color thanks to the modulated contributions from very strong blue emission of Ce^III^ centers and much weaker green emission of Tb^III^ complexes (see Figure 2 and the related subchapter). For the series of **10**–**14**, the set of room-temperature emission and excitation spectra were gathered (Figures 7 and 8 and Figure S19). For **10**, the emission spectra under all possible excitation from the UV range are very similar consisting of the dominant broadband Ce^III^-centered component with the maximum at 427 nm accompanied by the series of weaker sharp emission peaks of the Tb^III^ origin appearing at higher wavelengths. Thus, the overall emission color is blue, represented by the CIE 1931 chromaticity parameters slightly shifted in comparison to the **CeRu** compound **2** (Table S11). The excitation spectra both for the monitored Ce^III^- as well as Tb^III^-based emissions are almost identical, consisting of three bands assignable mainly to the Ce^III^ d-f electronic transitions and partially the Ru^II^ d-d electronic states (below 300 nm). Therefore, besides the dominant Ce^III^-based emission induced by the direct excitation supported by the Ru^II^-to-Ce^III^ energy transfer, **10** shows also the Tb^III^ emission peaks possibly realized by the partial Ce^III^-to-Tb^III^ and Ru^II^-to-Tb^III^ energy transfer pathways. The luminescence excitation routes significantly change for compounds **11**–**14** revealing the gradually increased excess of Tb^III^ centers. The excitation spectra for **11**–**13** remain similar both for the Ce^III^- and Tb^III^-centered emissions; however, two distinguishable maxima positioned around ca. 280 and 350 nm are observed. The higher-energy one can be mainly ascribed to the Tb^III^ d-f electronic transitions with the partial contribution from the Ru^II^ centers but without the significant Ce^III^ -based contribution as this metal ion is in the small amount for these materials. Such interpretation is indicated by the resulting emission, which is strongly dominated by the Tb^III^ emission peaks for the deep UV excitation (as shown for **13**, Figure S19). On the contrary, the excitation band in the 320–400 nm range remains dominated by the Ce^III^ d-f electronic transitions giving the strong blue emission of this metal ion even for the 1:100 Ce/Tb ratio in **13**. However, due to the increasing amount of Tb^3+^ ions, their emission contributes stronger, gradually shifting the overall emission toward the green color area also for the 350 nm excitation (Figure 7). Taking advantage of both these features, compound **14** of a tiny amount of Ce^3+^ ions (Tb_0.997_Ce_0.003_ 4f-metal composition) was prepared and examined (Figure 8). Under the 350 excitation, it shows the combined emission peaks of the Ce^III^ and Tb^III^ origins, resulting in an overall sky-blue emission; however, upon lowering the excitation wavelengths in the 350–300 nm range, the blue Ce^III^-based emission components gradually weaken leading to the emission color tuning toward the green emission. This multicolor emission tuning in **14** is depicted by the nearly linear variation from blue to green areas of the chromaticity diagram (Figure 8b, Table S11). For the 322.5 nm excitation, the related chromaticity parameters of *x* = 0.25 and *y* = 0.33 are even close to the white light emission, but a small red component lacks to achieve the pure WLE.

### Preparation, Structure, and Tunable Emission of Heterotri-Lanthanide Sm/Tb/Ce System, 15

Taking advantage of excitation-wavelength-dependent color tuning from blue to red and blue to green in the heterobi-lanthanide Sm/Ce and Tb/Ce compounds, respectively, we focused on the heterotri-lanthanide Sm/Tb/Ce systems. Aiming at the tunable white light emission (WLE), it was necessary to consider the proper relative amounts of three different lanthanide ions. The results both for the Sm/Ce series, **5**–**9**, and the Tb/Ce family, **10**–**14**, indicate that the emission color tuning is the most efficient for the extremely small amount of strongly emissive Ce^III^ centers, less than 1% in the 4f-metal content (Figures 5 and 8). Moreover, it was shown that both Sm^III^ as well as Tb^III^ centers, when used in the large excess, can produce the emission shift toward their characteristic red and green emission colors, respectively; however, the Sm^III^ emission is generally weaker than the Tb^III^ complexes as depicted by the investigation of mono-lanthanide compounds **3** and **4**. This may suggest that the Sm^3+^ ions should be employed in a slightly larger amount than Tb^3+^ ions. However, the excitation-variable emission tuning in the Sm/Ce and Tb/Ce materials of the optimized compositions, **9** and **14**, indicates that the emission for the Tb/Ce is much closer to the WLE area (Figures 5 and 8), which suggests that the smaller red component is needed to achieve the tunable white light emission. Following these lines, we synthesized and characterized the heterotri-lanthanide compound **15** using the molar Sm/Tb/Ce ratio of 400:600:1 (see Experimental Section for details) with the very small amount of Ce^3+^ ions, and the slight excess of Tb^3+^ ions over the Sm^3+^ ions. After preliminary characterization by the IR and UV–vis–NIR spectra, which are very similar to the mono-lanthanide **2**–**4** (Figures S20 and S22), compound **15** was characterized by the P-XRD technique, indicating its isostructurality with the orthorhombic phases of **3** (**SmRu**) and **4** (**TbRu**) (Figure S23 and Table S9) and its single-phase character (not the mixture of a few mono- or heterobi-lanthanide materials). As for all obtained materials, the crystal structure of **15** consists of a single crystallographic position for the lanthanide ion, and thus it is here occupied by three different lanthanides with partial occupancies. They are randomly distributed as checked by the SEM EDXMA measurements performed on a few crystals of **15** (Figure S24 and Table S10). The related results of the Ce/Sm and Ce/Tb ratios were also used to determine the exact composition of this material, which was supported by the CHN elemental analysis and thermogravimetry (Table 1 and Figure S21). The latter indicates also that the crystalline sample of **15** is stable in the air; however, upon heating, it gradually loses water molecules in a similar manner as observed for **1**–**4** and **5–15** (see above and compare Figures S2, S9, S15, and S21).

**Figure 9:**
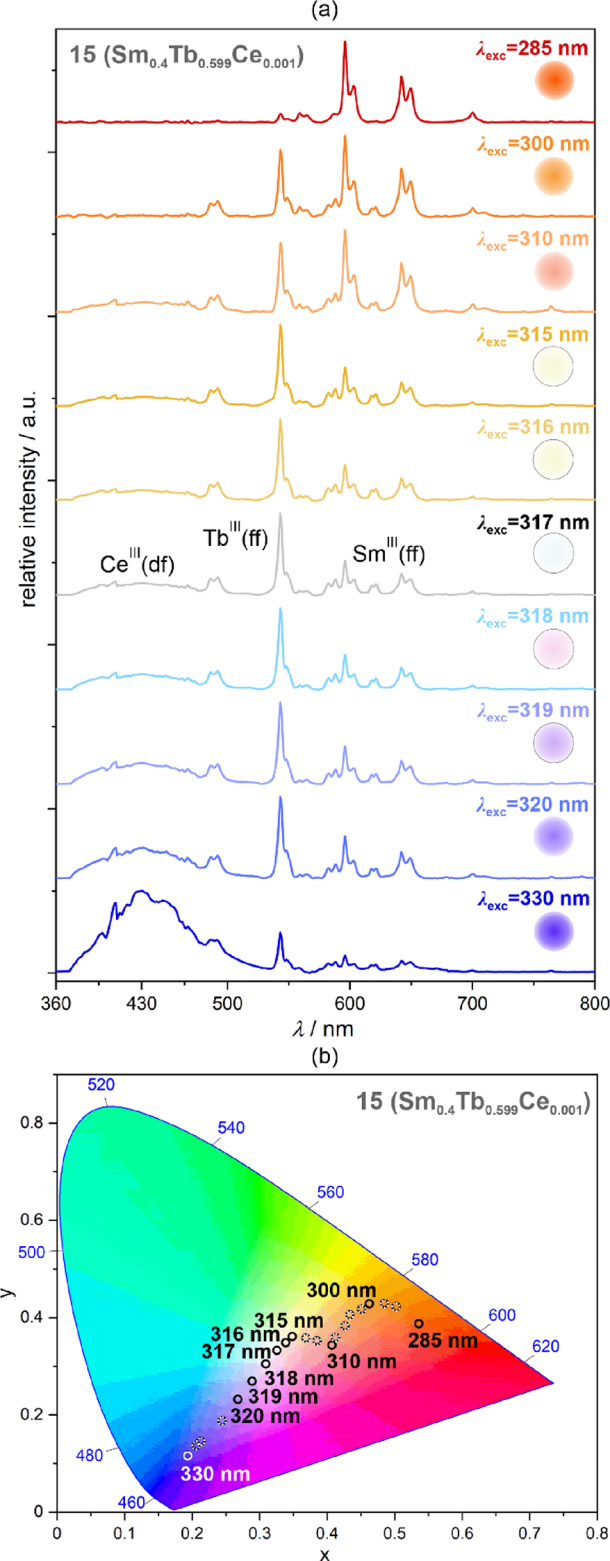
Room-temperature excitation-wavelength-variable emission spectra of **15** (Sm_0.4_Tb_0.599_Ce_0.001_ 4f-metal composition) (a) and the resulting emission colors shown on the CIE 1931 chromaticity diagram (b). The emission colors are also illustrated on the right side of each emission pattern in (a). The groups of electronic transitions responsible for the emission peaks are indicated in (a), while the detailed assignment is given for each lanthanide ion in Figure 2.

The room-temperature excitation-variable emission spectra for **15** are presented in Figure 9 and Figure S25. Due to the expected excitation-dependent combination of the blue emission component from the Ce^III^ d-f electronic transitions, the green emission component from the Tb^III^ f-f electronic transitions, and the red emission component from the Sm^III^ f-f electronic transitions, compound **15** exhibits rich emission spectra giving the overall various emission colors ranging from blue for the lowest energy 330 nm excitation to orange-red for the highest energy 285 nm excitation. Therefore, the wide range of emissions, part of them not accessible for the heterobi-lanthanide systems **5**–**14**, were achieved in **15** as represented by the CIE 1931 chromaticity parameters (Figure 9b and Table S11).

The rich excitation-dependent variation of emission in **15** takes advantage of the distinguishable regions of the efficient excitation for three embedded lanthanide ions, including the deep UV range favoring the Sm^III^ emission, the lowest energy UV region favoring the Ce^III^ emission, and partially the Tb^III^ one, while the intermediate UV range is suitable for all three lanthanide centers (see the excitation spectra, Figure S25b). For the 310–320 nm region of excitation, emission contributions from all three incorporated lanthanide ions become at a similar intensity level, which produces the white light emission characteristics. The purest WLE parameters of *x* = 0.325 and *y* = 0.333 is generated by the 317 nm excitation. Warm white light emission, realized by the small admixture of yellow color, is produced by the 315 and 316 nm excitations, while the 318 nm excitation induces cold white light emission, realized by the slightly increased blue emission component (Figure 9).

**Figure 10:**
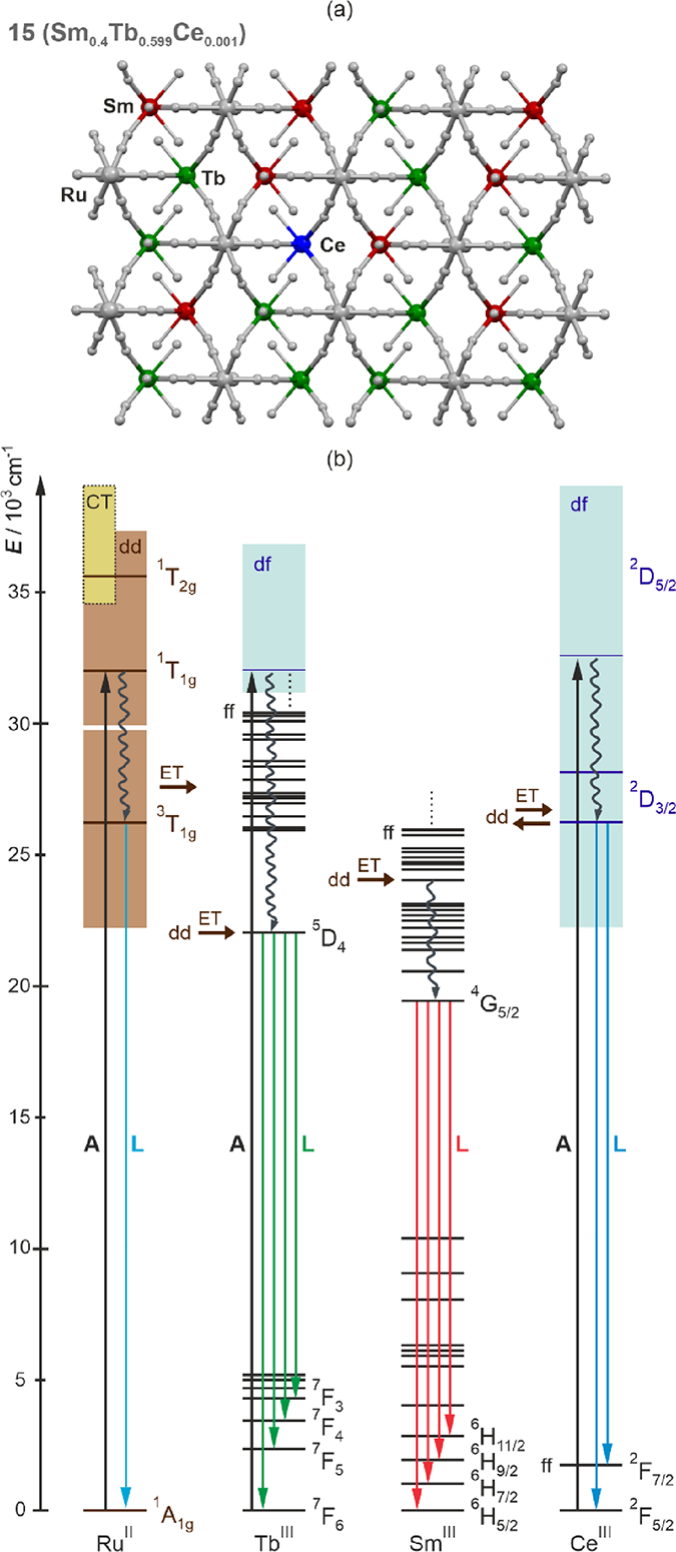
Schematic visualization of 4d- and 4f-metal centers dispersed within the coordination framework of **15** with the atom colors corresponding to the colors of emission contributions giving the white light emission for the 317 nm excitation (a) and the related schematic energy level diagram illustrating the observed luminescent effects for this excitation (b). The straight lines represent absorption (A) and luminescence (L), while the wavy lines show nonradiative relaxation. The energy transfer (ET) processes are shown as brown arrows. The states corresponding to the d-d, d-f, and f-f electronic transitions are indicated (dd, df, and ff labels, respectively). The high-energy charge-transfer (CT) states for [Ru^II^(CN)_6_]^4–^ complexes are also indicated. The Ru^II^-centered emission is presented (by the sky blue arrow) in the respective part of the diagram even though it is not observed for **15**; however, it was detected in **1** (**LaRu** analog, Figure 2). Therefore, the presented energy level diagram is valid for the other reported compounds with limitations related to the embedded set of lanthanide ions.

The emission quantum yield determined for the purest WLE at the 317 nm excitation reaches 0.9(2)%, which is much lower than the values of 59(5) and 5.4(5)% found for the mono-lanthanide **2** (**CeRu**) and **4** (**TbRu**) but slightly higher than the value of 0.6(1)% for **3** (**SmRu**). Thus, it is limited by the Sm^III^ component that has to be used in the large amount to reach the comparable emission intensity level to the much stronger Ce^III^-based emission. Nevertheless, the tunable WLE characteristics were generated in **15** thanks to the incorporation of three different lanthanide ions into the 3D coordination frameworks using the [Ru^II^(CN)_6_]^4–^ complexes as intermetallic linkers as visualized in Figure 10a.

The WLE functionality was achieved by the subtle equilibrium between four metal-based luminophores, three lanthanide complexes, and Ru^II^ cyanidometallate, utilizing various electronic transitions, which are summarized in Figure 10b. It is here worth discussing the roles of the respective metal complexes, in particular, the hexacyanidoruthenate(II) linker. This metal cyanido complex is transparent in the visible range as its absorption is fully shifted to the UV range; thanks to this, it primarily serves as a colorless molecular linker enabling the observation of emission properties of various colors, from blue to red, originating from the attached lanthanide complexes. It also enables the mixing of three different lanthanide ions, Ce^3+^, Sm^3+^, and Tb^3+^, within the single-phase material of the 3D coordination framework. The Ru^II^ cyanido complexes are weakly blue emissive but this luminescence (observable in the **LaRu** compound, **1** only at 77 K, Figure 2) does not contribute to the overall WLE pattern in **15** as the cyanido complexes transfer the absorbed UV light (strong absorption related to the d-d electronic transitions, Figure S4) toward lanthanide centers, especially toward Sm^III^ centers showing the red emission from their characteristic f-f electronic transitions (compare Figures 2 and 9). On the contrary, the Ru^II^ moieties rather weakly interact with the Ce^III^ and Tb^III^ complexes, including only the partial Ru^II^-to-lanthanide(III) energy transfer process, as was depicted by the emission characteristics of mono-lanthanide compounds, **2** (**CeRu**) and **4** (**TbRu**) (Figure 2). Therefore, the Tb^III^ and Ce^III^ complexes mainly employ the excitation routes through their accessible d-f electronic states. For Tb^III^ centers, such absorption is followed by the energy crossing to the emissive ^5^D_4_ level producing the green emission related to the series of f-f electronic transitions. For Ce^III^ centers, the d-f electronic transitions are emissive, and thus the fast and efficient emission of the nanosecond lifetime is observed. Overall, for the 310–320 nm excitation, all three lanthanide centers exhibit sufficiently good emission properties to produce the WLE pattern (Figure 9). The emission color tuning and the optimization of the WLE were achieved by the selection of excitation wavelength. This mainly takes advantage of the modulated selective excitation of the specific lanthanide ions; however, the role of the interlanthanide energy transfer can be non-negligible as was observed in the series of heterobi-lanthanide Sm/Ce compounds, **5–9** and Tb/Ce compounds **10**–**14** (see Figures 4, 5, 7 and 8, and the related sections above). It suggests the additional role of the Ru^II^ cyanido complexes that partially contribute to transmitting the Ce^III^-to-Sm^III^ and Ce^III^-to-Tb^III^ energy transfer pathways.

### Desolvation of Coordination Frameworks 1–15 and Its Influence on Photoluminescence

As the reversible dehydration of lanthanide–hexacyanidometallate networks was shown to be an efficient route for the improvement and switching of optical, magnetic, and mechanical properties,^78,91^ we have tested the emission properties after thermal dehydration for two selected compounds, mono-lanthanide **2** (**CeRu**) and heterotri-lanthanide **15** (Figure S26). The dehydration procedure follows the results of the TGA showing that the fully dehydrated phase can be achieved upon heating above 150 °C (Figures S2, S9, S15, and S21). For **2**, the dehydration leads to the small blueshift of the observed broadband blue Ce^III^-centered emission, weakly affecting the overall emission intensity (Figure S26a). For **15**, the overall emission intensity becomes much weaker after dehydration, which means that the presumable desolvation-induced change of the lanthanide coordination geometry from square antiprismatic to octahedral, as well as the possible increase of the structural disorder after desolvation, are not beneficial for the observed lanthanide emissions. The smallest decrease in the emission intensity was found for the Sm^III^-centered component. This trend can be assigned to the removal of water molecules, which are usually responsible for quenching the lanthanide emissions, especially for those showing lower energy emissive electronic transitions, such as Sm^III^ in comparison to Tb^III^ or Ce^III^. As the result, the emission pattern after dehydration dramatically changes, e.g., at the 317 nm excitation, leaving mainly the weak red Sm^III^-based emission instead of the WLE (Figure S26bc). Therefore, the desolvated phases were not further characterized as the objective property of white light emission is quenched indicating that the as-synthesized hydrated phases reveal the richer emission functionalities.

### Magnetic Properties of Mono-Lanthanide Compounds, **2**–**4**

Lanthanide(III)-containing luminescent coordination compounds, including those based on cyanido transition metal complexes, often reveal the multifunctional character linking optical functionalities with significant magnetic anisotropy leading to the single-molecule magnet (SMM) behavior.^77−81^ As paramagnetic lanthanide(III) complexes are incorporated in the structures of **2**–**4**, their magnetic properties were examined in the context of the potential SMM character (Figures S27–S29 and the related discussion in the Supporting Information). All these compounds exhibit typical direct-current (DC) magnetic characteristics related to the single-ion properties of paramagnetic Ce^III^, Sm^III^, and Tb^III^ complexes separated in the respective coordination frameworks by diamagnetic hexacyanidoruthenate(II) ions (Figure S27). The distinct contribution from the not fully suppressed magnetic interactions is observed only for the Tb^III^-containing compound **4**. The alternating-current (AC) magnetic measurements reveal that the lack of slow magnetic relaxation effects in **3** and **4**, while the Ce^III^-containing compound **2** exhibits only the onset of field-induced slow magnetic relaxation as represented by the non-negligible out-of-phase magnetic susceptibility detected for the highest accessible frequency range at very low temperatures below 3 K. This indicates the very weak magnetic anisotropy in the reported compounds, which can be explained by the high coordination numbers without the presence of strongly coordinating negatively charged ligands, e.g., of the organic-oxide-type, that could provide the distinct SMM effect.^77−81^

## Conclusions

Here, we report the novel family of functional solid luminophores based on heterometallic d-f coordination frameworks incorporating visible light emissive lanthanide ions (Ce^3+^, Sm^3+^, and Tb^3+^) and rarely explored blue phosphorescent hexacyanidoruthenate(II) metalloligands. Aiming at the tunable multicolor to white-light emission characteristics, we selected three above-mentioned, differently luminescent lanthanide ions (blue emissive Ce^3+^, red emissive Sm^3+^, and green emissive Tb^3+^) and incorporated them into the lanthanide–hexacyanidometallate networks obtaining mono-lanthanide **CeRu**, **SmRu**, and **TbRu** materials showing the room-temperature emission properties, specific for each lanthanide. The emission efficiencies and detailed parameters, such as emission lifetime, were found to be strongly dependent on the lanthanide due to the different natures of their electronic transitions and variable interactions with d-d electronic states of Ru^II^-cyanido linkers, including d-f electronic transitions giving short-lived but very strong (quantum yield of 59(5)%) emission for Ce^III^ and f-f electronic transitions giving longer-lived but weaker emission for Tb^III^ (quantum yield of 5.4(5)%, the excitation mainly from their d-f electronic states) and Sm^III^ (quantum yield of 0.6(1)%, the deep UV excitation by the energy transfer from Ru^II^). Taking advantage of these lanthanide emission properties, we prepared a series of heterobi-lanthanide Sm/Ce and Tb/Ce compounds for which the dominance of Ce^III^-based emission could be overcome by the large excess of the second lanthanide ion. Thus, for the compounds with the large Sm/Ce and Tb/Ce molar ratios, the excitation-wavelength tunable multicolor photoluminescence, ranging from blue to red and blue to green, respectively, was achieved. By exploring the whole set of three emission components from Sm^III^, Tb^III^, and Ce^III^ in the heterotri-lanthanide material of the optimized K{Sm_0.4_Tb_0.599_Ce_0.001_(H_2_O)_2_[Ru(CN)_6_]}·2.5H_2_O composition, not only the multicolor blue to orange emission color tuning was achieved but also the adjustable white light emission (WLE) characteristics were generated. The WLE effect was here realized by adjusting the similar emission intensity level for red Sm^III^, green Tb^III^, and blue Ce^III^ emission components, and thus their detailed ratios were easily modulated by the excitation wavelengths. As the result, the obtained heterotri-lanthanide system shows room-temperature WLE of various hues, including pure white light, as well as warm or cold white light emissions. Therefore, in this work, we presented an elegant and simple synthetic pathway to achieve rich tunable multicolor and white-light emission realized by playing with the d-d, d-f, and f-f electronic transitions of metal-based molecular luminophores, hexacyanidoruthenate(II), and lanthanide ions, combined into the three-dimensional coordination polymer. The challenge for future work remains in the increase of the overall emission intensity, especially for the WLE signal, which was limited by the weakest emission component of Sm^III^ centers. The straightforward solution of using the strongly red emissive Eu^III^ centers instead of Sm^III^ ones could not be successful as the low-lying and luminescence quenching charge transfer states appear when Eu^III^ centers are bridged to Ru^II^ centers. Therefore, future work may consist of searching for the proper polycyanidometallate complex that can reveal similar optical properties as hexacyanidoruthenate(II) without the charge transfer (CT) affinity to Eu^III^ centers. Such candidates, including heavy atom Ir^III^ or Pt^IV^ cyanido complexes that usually do not form CT states with Eu^III^, will be the subject of our future work toward more efficient multicolor and white-light emitters based on heterometallic d-f coordination systems. The other aspect of the future work should consist of the application of the obtained materials for LED fabrication, which will demand the processing of the compounds to thin films and testing of their electroluminescence properties. In particular, the construction of the WLED system from the prepared coordination frameworks is worth checking, either using the UV LED chip covered by the material or by the exploration of the possible electroluminescence from the material that will follow the recent trends in the research field.^103^

## Supporting Information Available

Infrared (IR) absorption spectra; UV–vis–NIR absorption spectra; thermogravimetric (TG) curves; crystal and structure refinement data; detailed structural views and structural parameters; results of continuous shape measure analyses for the coordination polyhedrons; powder X-ray diffraction patterns and indexing results; results of SEM EDXMA together with the related SEM pictures; and additional excitation and emission spectra together with the emission decay profiles for selected compounds (PDF)
