# Supplementary material for: Lanthanide Hexacyanidoruthenate Frameworks for Multicolor to White-Light Emission Realized by the Combination of d-d, d-f, and f-f Electronic Transitions
Source: Inorg Chem. 2023 Jan 19;62(4):1611–27. doi: 10.1021/acs.inorgchem.2c03885 (PMC9890488; doi:10.1021/acs.inorgchem.2c03885)
Supplement: Supplementary file 1 — ic2c03885_si_001.pdf [file ic2c03885_si_001.pdf]

SUPPORTING INFORMATION

Lanthanide Hexacyanidoruthenate Frameworks for Multi-Color to White-Light Emission Realized by the Combination of d-d, d-f, and f-f Electronic Transitions

Tomasz Charytanowicz, Barbara Sieklucka, Szymon Chorazy*

Faculty of Chemistry, Jagiellonian University, Gronostajowa 2, 30-387 Krakow, Poland.
*Corresponding author: simon.chorazy@uj.edu.pl

Infrared (IR) absorption spectra of **1**–**4**. (Figure S1)	S2
Thermogravimetric (TG) curves of **1**–**4**. (Figure S2)	S3
Views of the asymmetric units of **1**–**4**. (Figure S3)	S4
Solid-state room-temperature UV-vis-NIR absorption spectra of **1**–**4**. (Figure S4)	S5
Details of crystal data and structure refinement for **1**–**4**. (Table S1)	S6
Detailed structural parameters for **1**–**4**. (Table S2)	S7
Result of Continuous Shape Measure Analysis for $[Ru^{II}(CN)_6]^{4-}$ complexes in **1**–**4**. (Table S3)	S8
Result of Continuous Shape Measure Analysis for Ln(III) complexes in **1**–**4**. (Table S4)	S8
Powder X-ray diffraction (P-XRD) patterns of **1**–**4**. (Figure S5)	S9
Emission decay profiles of **1**–**4**. (Figure S6)	S10
Excitation and emission spectra of **2** at 77 K, and the comparison of the emissions of **1** and **2**. (Figure S7)	S11
Infrared (IR) absorption spectra of **5**–**9**. (Figure S8)	S12
Thermogravimetric (TG) curves of **5**–**9**. (Figure S9)	S13
Solid-state room-temperature UV-vis-NIR absorption spectra of **5**–**9**. (Figure S10)	S14
P-XRD patterns of **5**–**9** compared with the P-XRD patterns of **2** and **3**. (Figure S11)	S15
Comparison of the unit cell parameters obtained from the P-XRD patterns for **2**, **3**, and **5**–**9**. (Table S5)	S16
Representative SEM images of the microcrystals of **5**–**9**. (Figure S12)	S17
Results of the SEM EDXMA microanalysis for **5**–**9**. (Table S6)	S18
Emission spectra for two selected excitation wavelengths for **8** and the related emission colors shown on the CIE 1931 chromaticity diagram. (Figure S13)	S19
Infrared (IR) absorption spectra of **10**–**14**. (Figure S14)	S20
Thermogravimetric (TG) curves of **10**–**14**. (Figure S15)	S21
Solid-state room-temperature UV-vis-NIR absorption spectra of **10**–**14**. (Figure S16)	S22
P-XRD patterns of **10**–**14** compared with the P-XRD patterns of **2** and **4**. (Figure S17)	S23
Comparison of the unit cell parameters obtained from the P-XRD patterns for **2**, **4**, and **10**–**14**. (Table S7)	S24
Representative SEM images of the microcrystals of **10**–**14**. (Figure S18)	S25
Results of the SEM EDXMA microanalysis for **5**–**9**. (Table S8)	S26
Emission spectra for three selected excitation wavelengths for **13** and the related emission colors shown on the CIE 1931 chromaticity diagram. (Figure S19)	S27
Infrared (IR) absorption spectrum of **15**. (Figure S20)	S28
Thermogravimetric (TG) curve of **15**. (Figure S21)	S29
Solid-state room-temperature UV-vis-NIR absorption spectrum of **15**. (Figure S22)	S30
P-XRD pattern of **15** compared with the P-XRD patterns of **2**, **3**, and **4**. (Figure S23)	S31
Comparison of the unit cell parameters obtained from the P-XRD patterns for **2**, **15**, **3**, and **4**. (Table S9)	S32
Representative SEM images of the microcrystals of **15**. (Figure S24)	S33
Results of the SEM EDXMA microanalysis for **15**. (Table S10)	S34
Additional emission and extitation spectra of **15**. (Figure S25)	S35
Optical characteristics after thermal dehydration of the polycrystalline samples of **2** and **15**. (Figure S26)	S36
Summary of the *x* and *y* CIE 1931 chromaticity parameters for the emissions colors of **1**–**15**. (Table S11)	S37
Direct-current (*dc*) magnetic properties of **2**, **3**, and **4**. (Figure S27)	S38
The *dc*-field-dependent *ac* magnetic susceptibility curves for **2** at 1.8 K. (Figure S28)	S39
The temperature-dependent *ac* magnetic susceptibility curves for **2** at the *dc* field of 1 kOe. (Figure S29)	S40
Discussion on magnetic properties of **2**–**4** (Comment to Figures S27–S29).	S41
References to Supporting Information.	S43

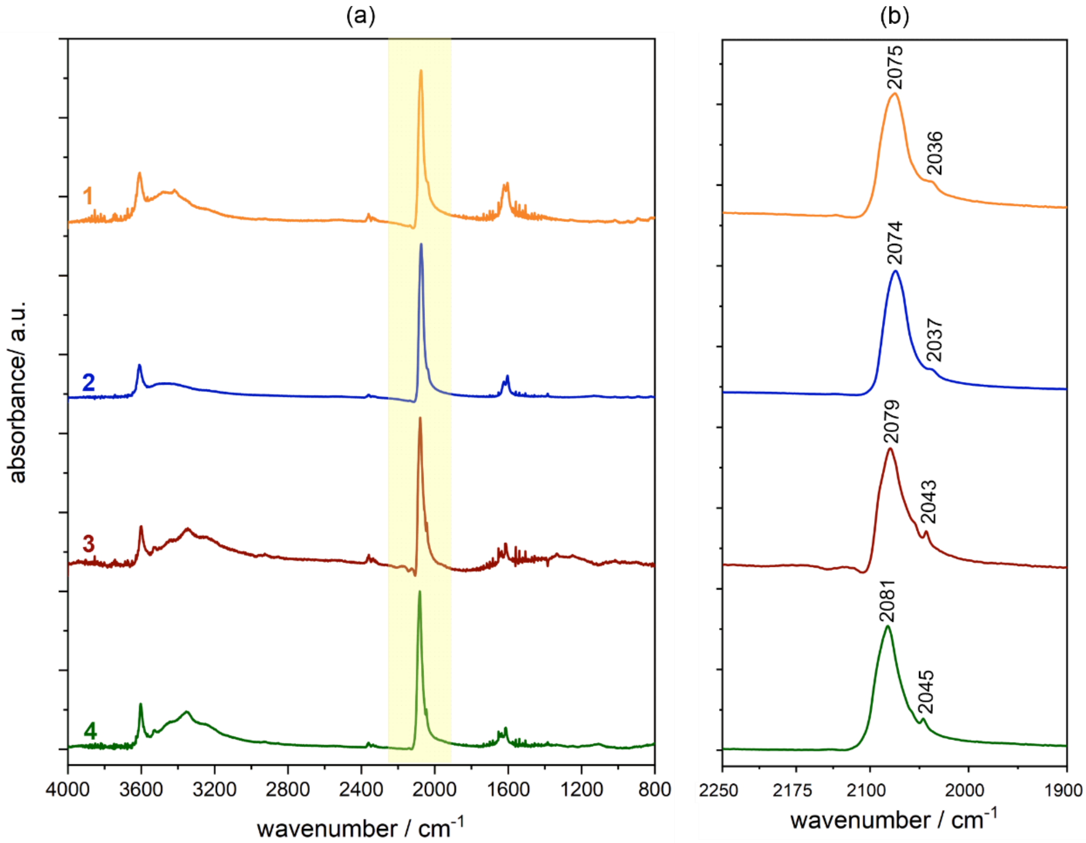

Figure S1. Infrared (IR) absorption spectra of **1**–**4**, measured in the 4000–800 cm^{-1} range (a), and the enlargement of the 2250–1900 cm^{-1} region (b), related to the stretching vibrations of cyanido ligands within hexacyanido-ruthenate(II) complexes.[S1–S3]

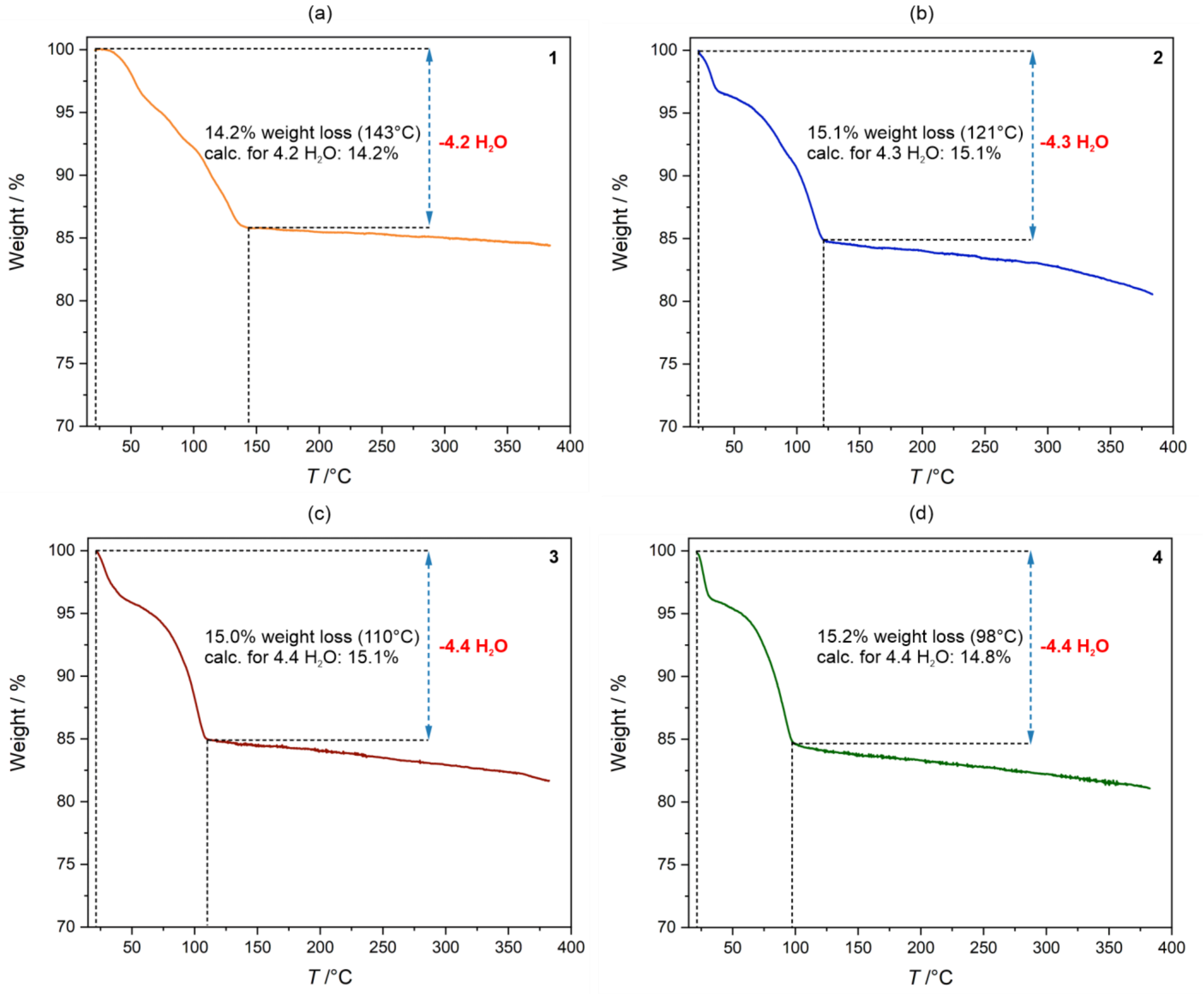

Figure S2. Thermogravimetric (TG) curves of **1** (a), **2** (b), **3** (c), and **4** (d), measured under a nitrogen atmosphere upon the continuous heating with the 1 °C·min^{-1} rate. The step related to the temperature-induced removal of water molecules is indicated on the graph. A few distinguishable steps within the indicated ranges are related to the subsequent removal of non-coordinated (lower temperatures) and coordinated (higher temperatures) water molecules.

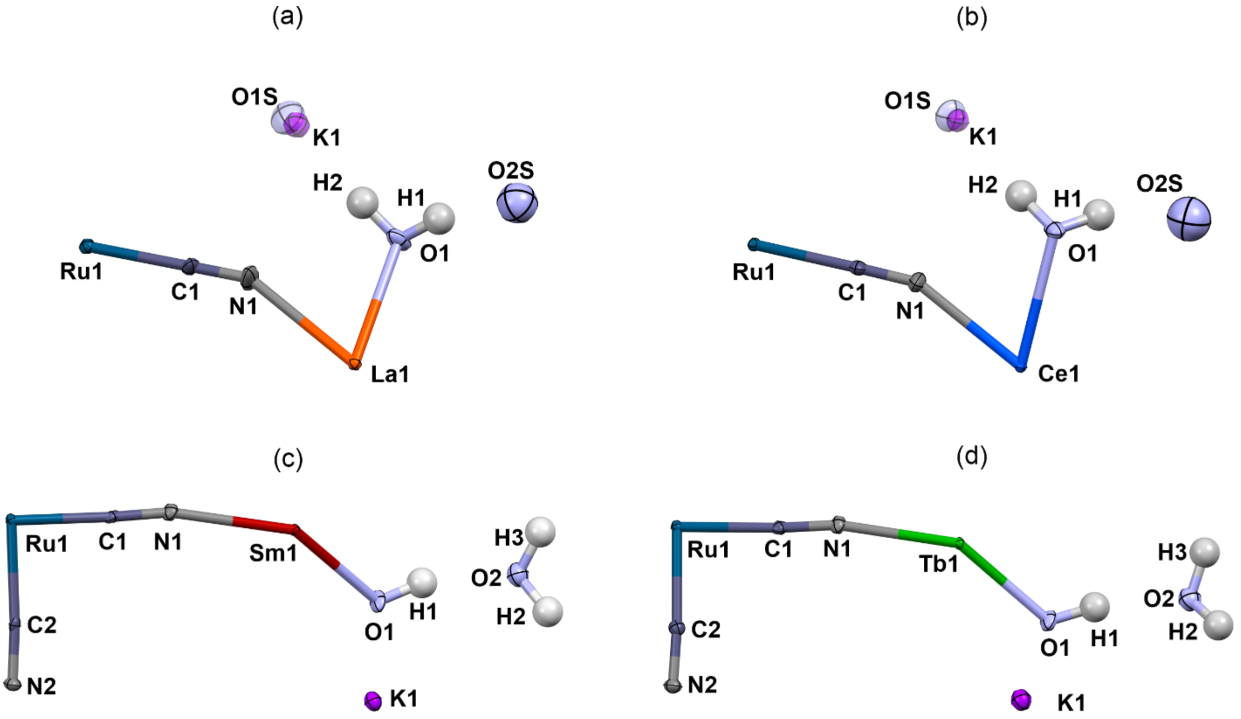

Figure S3. Asymmetric units of **1** (a), **2** (b), **3** (c), and **4** (d). Thermal ellipsoids are presented at the 50% probability level.

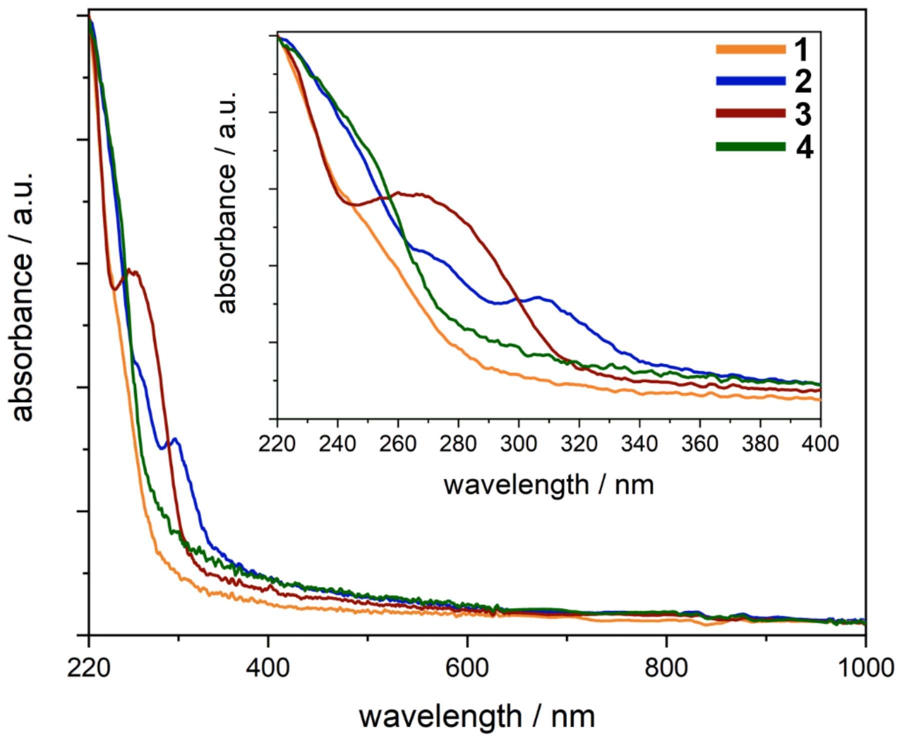

Figure S4. Solid-state room-temperature UV-vis-NIR absorption spectra of **1**–**4** in the 220–1000 nm range, together with the enlargement of the 220–400 nm region presented in the inset. The spectra were normalized to the intensity at 220 nm.

Table S1. Details of crystal data and structure refinement for **1–4**.

	**1**	**2**	**3**	**4**
**CCDC no.**	2201167	2201168	2201169	2201170
**Temperature / K**	100(2)	100(2)	100(2)	100(2)
**Formula**	$K_1La_1Ru_1C_6H_6N_6O_{4.20}$	$K_1Ce_1Ru_1C_6H_6N_6O_{4.30}$	$K_1Sm_1Ru_1C_6H_8N_6O_4$	$K_1Tb_1Ru_1C_6H_8N_6O_4$
**Formula weight / g·mol^{-1}**	508.45	511.26	518.70	527.27
**Radiation**	MoKα (λ = 0.71073)	MoKα (λ = 0.71073)	MoKα (λ = 0.71073)	MoKα (λ = 0.71073)
**Crystal system**	hexagonal	hexagonal	orthorhombic	orthorhombic
**Space group**	$P6_3$/m	$P6_3$/m	*C*mcm	*C*mcm
a **/ Å**	7.4724(3)	7.4576(2)	7.5177(3)	7.4500(3)
b **/ Å**	7.4724(3)	7.4576(2)	12.7846(5)	12.7174(5)
c **/ Å**	14.2210(8)	14.1575(7)	14.3529(6)	14.2753(5)
**Volume / Å^3**	687.67(7)	681.89(5)	1379.47(10)	1352.51(9)
Z	2	2	4	4
**ρ_{calc} / g·cm^{-3}**	2.456	2.490	2.498	2.589
**Absorption coefficient / cm^{-1}**	4.484	4.728	5.629	6.628
***F*(000)**	475	479	972	984
**Crystal shape**	plate	block	plate	plate
**Crystal color**	colorless	colorless	colorless	colorless
**Crystal size / mm x mm x mm**	0.17 x 0.08 x 0.04	0.17 x 0.13 x 0.12	0.18 x 0.10 x 0.02	0.12 x 0.10 x 0.07
**θ range / °**	2.865–27.447	2.878–27.480	2.838–27.482	2.854–27.439
**Limiting indices**	$-9 < h < 9$ $-6 < k < 9$ $-18 < l < 18$	$-8 < h < 9$ $-9 < k < 9$ $-18 < l < 18$	$-9 < h < 8$ $-13 < k < 16$ $-18 < l < 18$	$-9 < h < 9$ $-16 < k < 16$ $-18 < l < 18$
**Reflections collected**	3855	11418	5851	9582
**Unique reflections**	554	551	887	873
**R_{int}**	0.0437	0.0309	0.0323	0.0270
**Completeness / %**	100.0	100.0	100.0	100.0
**Data/restraints/ parameters**	554/11/41	551/11/42	887/5/62	873/5/62
**GOF on F^2**	1.288	1.306	1.297	1.122
**Final *R* indices**	R_1 = 0.0364 [$I > 2\sigma(I)$] wR_2 = 0.0642 (all data)	R_1 = 0.0167 [$I > 2\sigma(I)$] wR_2 = 0.0434 (all data)	R_1 = 0.0299 [$I > 2\sigma(I)$] wR_2 = 0.0576 (all data)	R_1 = 0.0225 [$I > 2\sigma(I)$] wR_2 = 0.0546 (all data)
**Largest diff. peak/hole / e·Å^{-3}**	0.720/–2.379	0.367/–1.601	1.121/–2.132	0.895/–1.325

Table S2. Detailed structural parameters for **1–4**.

parameter (bond or angle)	**1**	**2**
Ln1-N1 / Å	2.583(5)	2.551(3)
Ln1-O1 / Å	2.738(5)	2.728(3)
Ru1-C1 / Å	2.017(5)	2.019(3)
K1-O1 / Å	2.879(17)	2.868(10)
K1-N1 / Å	3.141(6)	3.167(3)
K1-C1 / Å	3.381(5)	3.381(3)
O1-O1S / Å	3.090	3.086
O1-O2S / Å	2.901	2.910
Ln1-Ru1 / Å	5.590	5.574
Ln-K1 / Å	4.807	4.817
N1-Ln1-N1 / °	85.4(3)/136.88(9)/79.1(2)	85.55(15)/136.94(6)/78.94(12)
N1-Ln1-O1 / °	69.31(14)/137.28(13)/67.58(14)	69.05(8)/137.22(8)/67.89(8)
O1-Ln1-O1 / °	120.0000(10)	120.0000(10)
C1-Ru1-C1 / °	90.6(2)/89.4(2)/180.0(2)	90.69(12)/89.31(12)/180.00(14)
Ru1-C1-N1 / °	179.4(5)	179.0(3)
Ln1-C1-N1 / °	152.5(4)	154.0(3)
parameter (bond or angle)	**3**	**4**
Ln1-N1 / Å	2.471(4)	2.431(3)
Ln1-N2 / Å	2.536(5)	2.490(5)
Ln1-O1 / Å	2.481(5)	2.432(4)
Ru1-C1 / Å	2.028(4)	2.031(4)
Ru1-C2 / Å	2.033(6)	2.030(6)
K1-O1 / Å	2.900(5)	2.899(5)
K1-N1 / Å	3.045(4)	3.035(4)
K1-N2 / Å	3.161	3.122
K1-C1 / Å	2.923(4)	2.919(4)
K1-C2 / Å	3.021(6)	2.995(6)
O1-O2 / Å	2.827	2.821
Ln1-K1 / Å	3.781	3.752
N1-Ln1-N1 / °	76.01(17)/ 121.08(17)/75.99(17)	76.17(16)/ 121.29(17)/76.02(16)
N2-Ln1-N2 / °	112.0(2)	111.4(2)
N1-Ln1-N2 / °	76.40(13)/141.76(9)	76.52(12)/141.80(8)
O1-Ln1-O1 / °	100.2(2)	101.2(2)
O1-Ln1-N1 / °	141.99(8)/80.99(13)	141.91(8)/80.54(12)
O1-Ln1-N2 / °	68.97(9)	69.02(8)
C1-Ru1-C1 / °	90.7(2)/89.3(2)/180.0	90.8(2)/89.2(2)/180.0
C1-Ru1-C2 / °	90.30(16)/89.70(16)	90.07(16)/89.93(16)
C2-Ru1-C2 / °	180.0	180.0
Ln1-C1-N1 / °	166.6(3)	167.0(3)
Ln1-C2-N2 / °	154.8(5)	156.1(5)
Ru1-C1-N1 / °	176.8(4)	176.0(4)
Ru1-C2-N2 / °	175.3(5)	174.6(5)

Table S3. The results of Continuous Shape Measure Analysis for six-coordinated Ru(II) complexes embedded in the crystal structures of **1–4**. The $S_{geometry}$ parameter adopting the value close to zero indicates that the experimental coordination geometry is very similar to the idealized one. The increase of the $S_{geometry}$ parameter represents the deviation from an ideal polyhedron. Considered coordination polyhedrons: **PPY** – a pentagonal pyramid, **OC** – an octahedron, and **TPR** – a trigonal prism. The determined coordination geometry is indicated by the orange background.[S4–S6]

	S_{PPY}	S_{OC}	S_{TPR}
**Ru1 (1)**	30.125	0.005	16.676
**Ru1 (2)**	30.034	0.008	16.659
**Ru1 (3)**	30.184	0.003	16.661
**Ru1 (4)**	30.266	0.003	16.649

Table S4. The results of Continuous Shape Measure Analysis for nine-coordinated Ln(III) complexes embedded in the crystal structures of **1–2** and eight-coordinated Ln(III) complexes embedded in the crystal structures of **3–4**. The $S_{geometry}$ parameter adopting the value close to zero indicates that the experimental coordination geometry is very similar to the idealized one. The increase of the $S_{geometry}$ parameter represents the deviation from an ideal polyhedron. Considered coordination polyhedrons: **CSARP** – a spherical capped square antiprism, **JTCTPR** – a tricapped trigonal prism J51, **TCTPR** – a spherical tricapped trigonal prism, **SAPR** – a square antiprism, **TDD** – a triangular dodecahedron, and **BTPR** – a bicapped trigonal prism. The determined coordination geometry is indicated by the orange background.[S4–S6]

	S_{CSAPR}	S_{JTCTPR}	S_{TCTPR}
**La1 (1)**	1.150	1.097	0.682
**Ce1 (2)**	1.165	1.012	0.685
	S_{SAPR}	S_{TDD}	S_{BTPR}
**Sm1 (3)**	0.456	2.184	1.177
**Tb1 (4)**	0.413	2.259	1.234

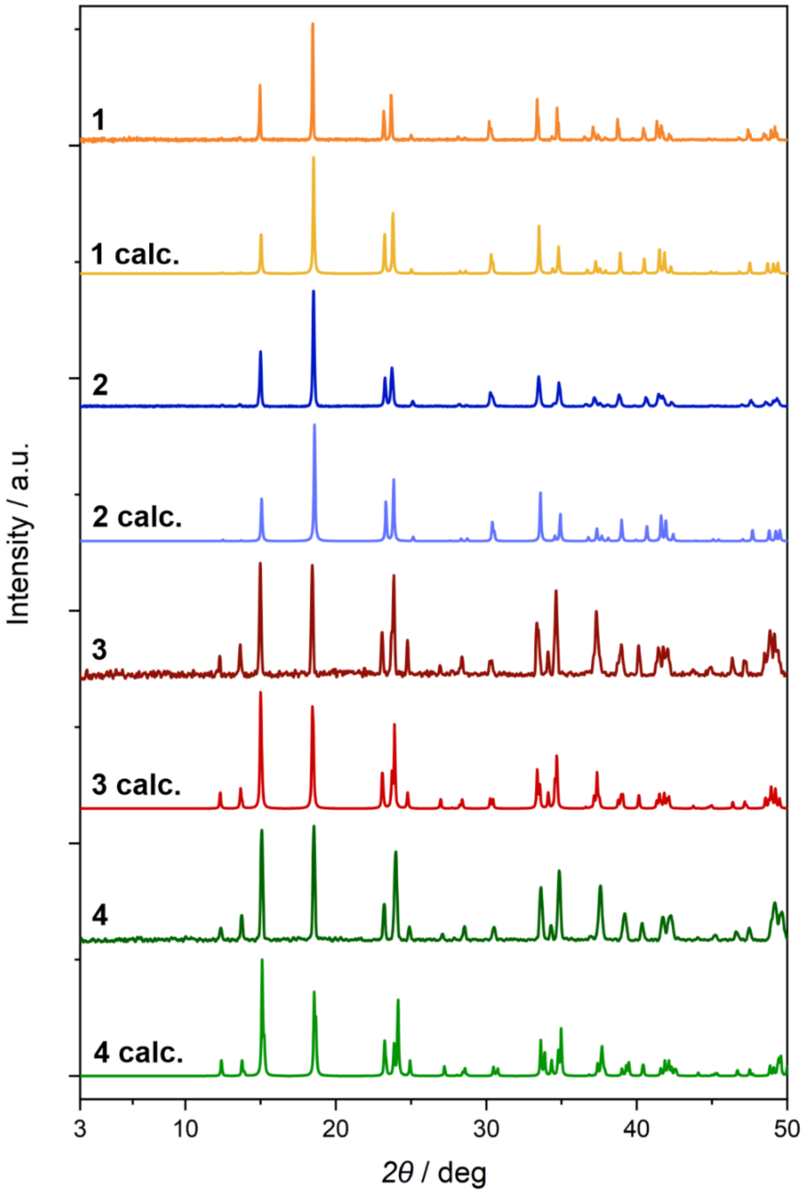

Figure S5. Experimental powder X-ray diffraction (P-XRD) patterns of the polycrystalline samples of **1**–**4**, compared with the P-XRD patterns calculated on the basis of the respective structural models achieved from the single-crystal X-ray (SC-XRD) structural analysis (Table S1).

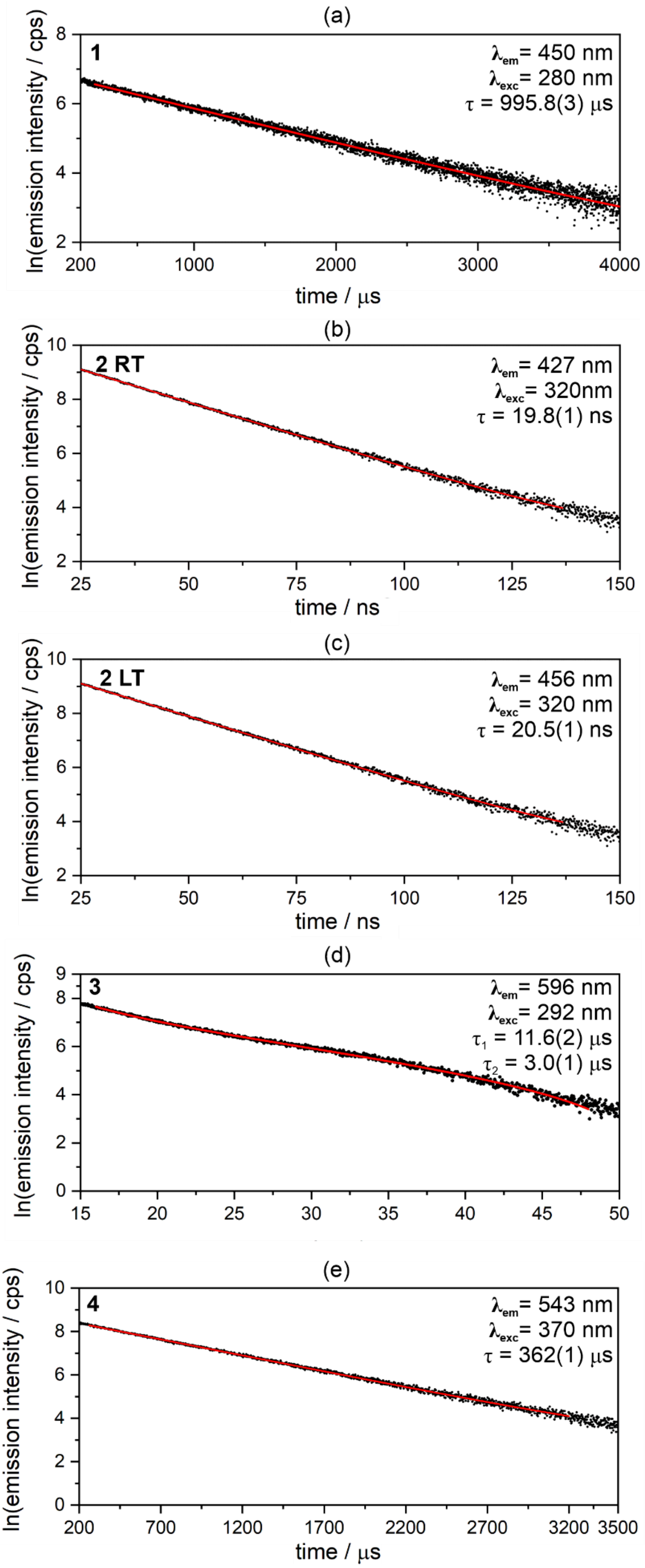

Figure S6. Emission decay profiles of **1** (77 K, a), **2** (room temperature, RT, b), **2** (77 K, low temperature, LT, c), **3** (RT, d), and **4** (RT, e). The black points show the experimental data while the red lines show the best-fit curves using the monoexponential decay function. The resulting emission lifetimes are presented on the graphs.

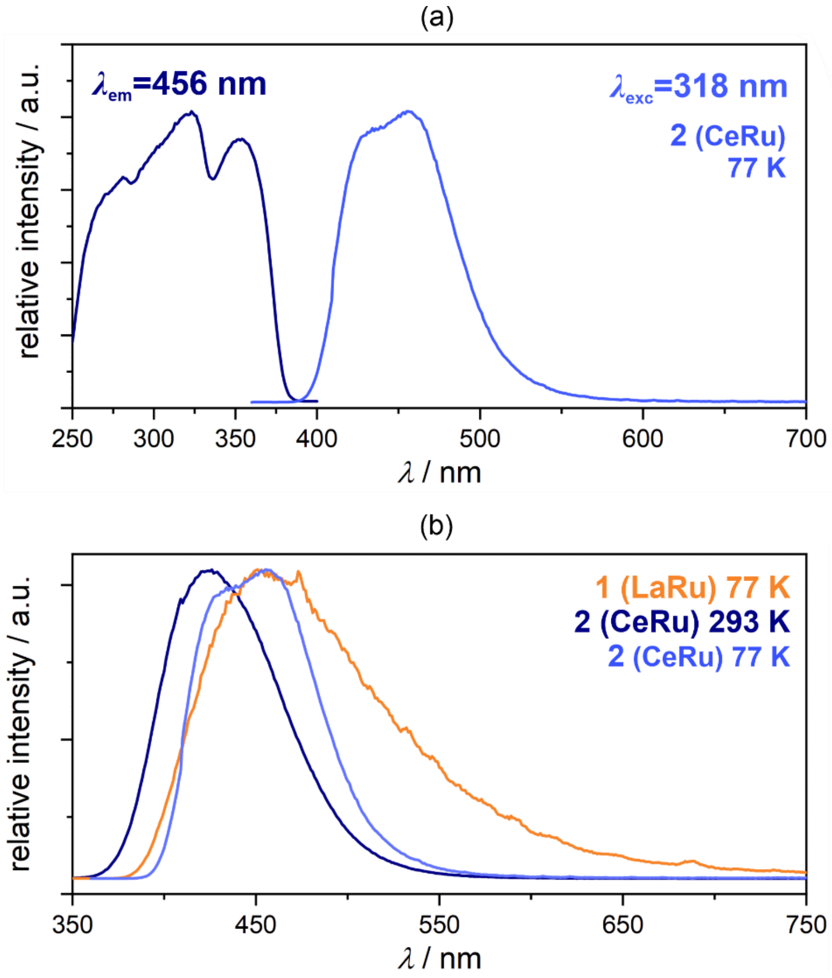

Figure S7. Low-temperature (77 K) excitation and emission spectra of **2** (a), and the comparison between the emission spectra of **1** at 77 K, **2** at 293 K, and **2** at 77 K (for the maxima of the respective excitation spectra, b).

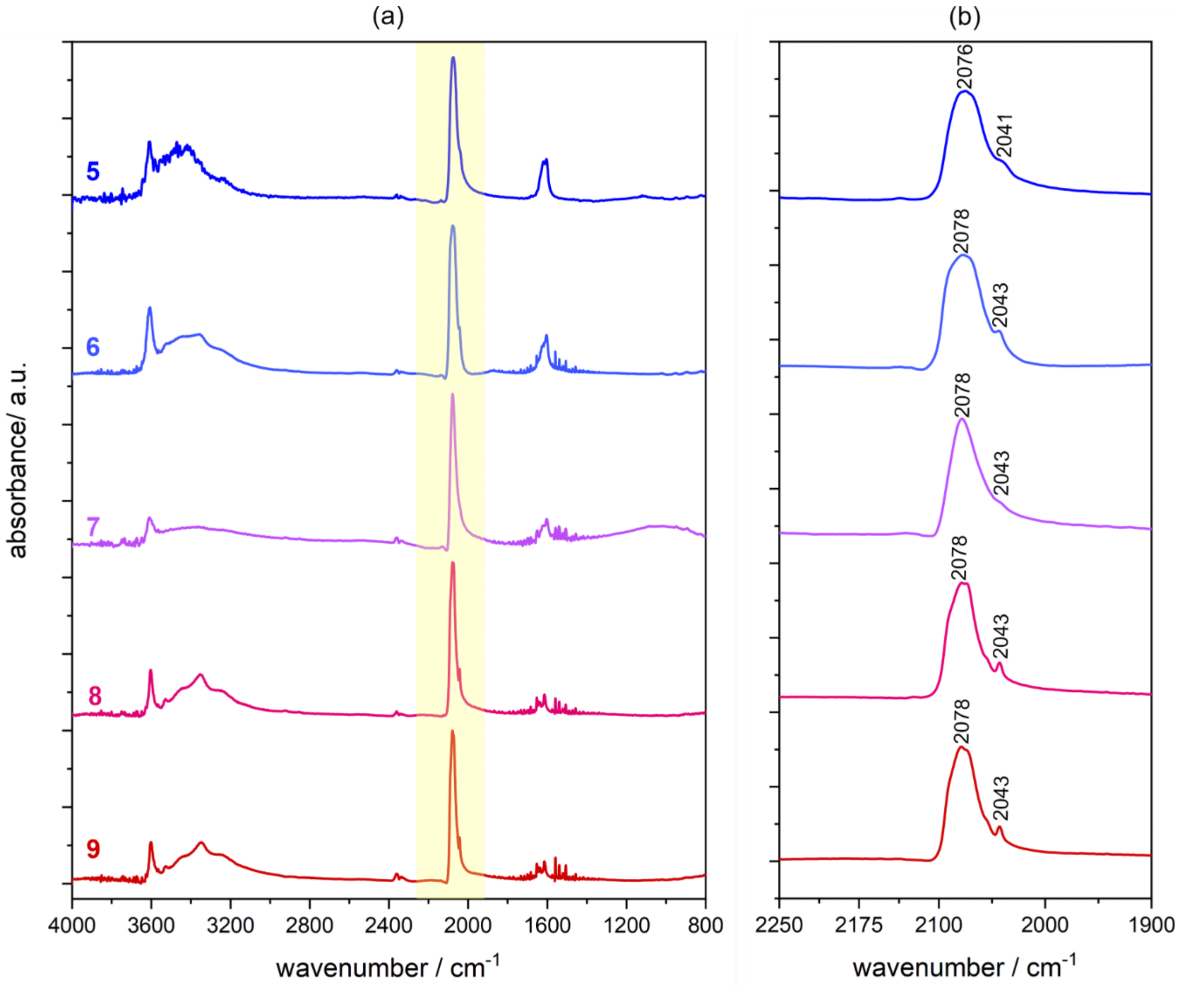

Figure S8. Infrared (IR) absorption spectra of **5**–**9**, measured in the 4000–800 cm^{-1} range (a), and the enlargement of the 2250–1900 cm^{-1} region (b), related to the stretching vibrations of cyanido ligands within hexacyanido-ruthenate(II) complexes.[S1–S3]

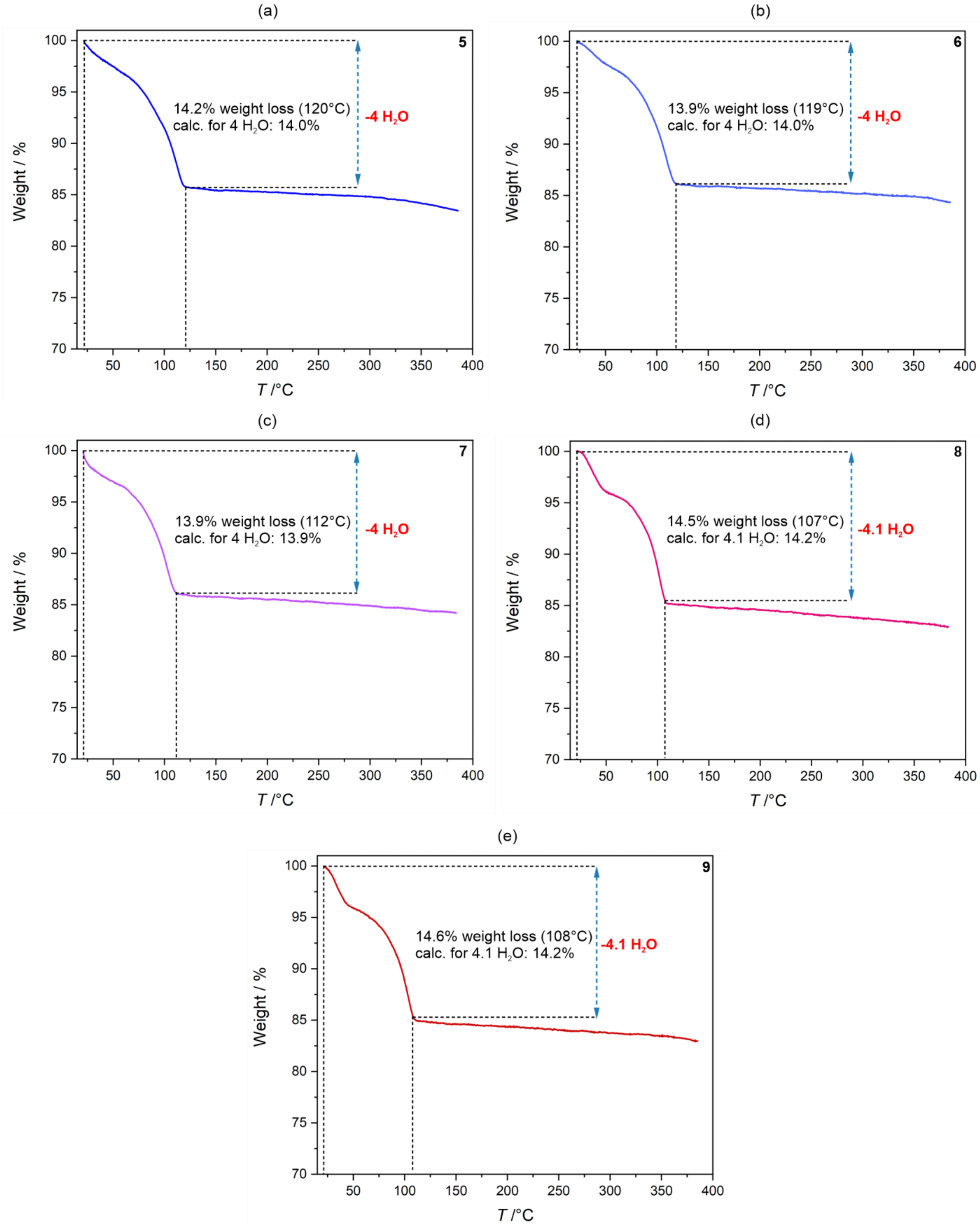

Figure S9. Thermogravimetric (TG) curves of **5** (a), **6** (b), **7** (c), **8** (d), and **9** (e), measured under a nitrogen atmosphere upon the continuous heating with the 1 °C·min^{-1} rate. The step related to the temperature-induced removal of water molecules is indicated on the graph. A few distinguishable steps within the indicated ranges are related to the subsequent removal of non-coordinated (lower temperatures) and coordinated (higher temperatures) water molecules.

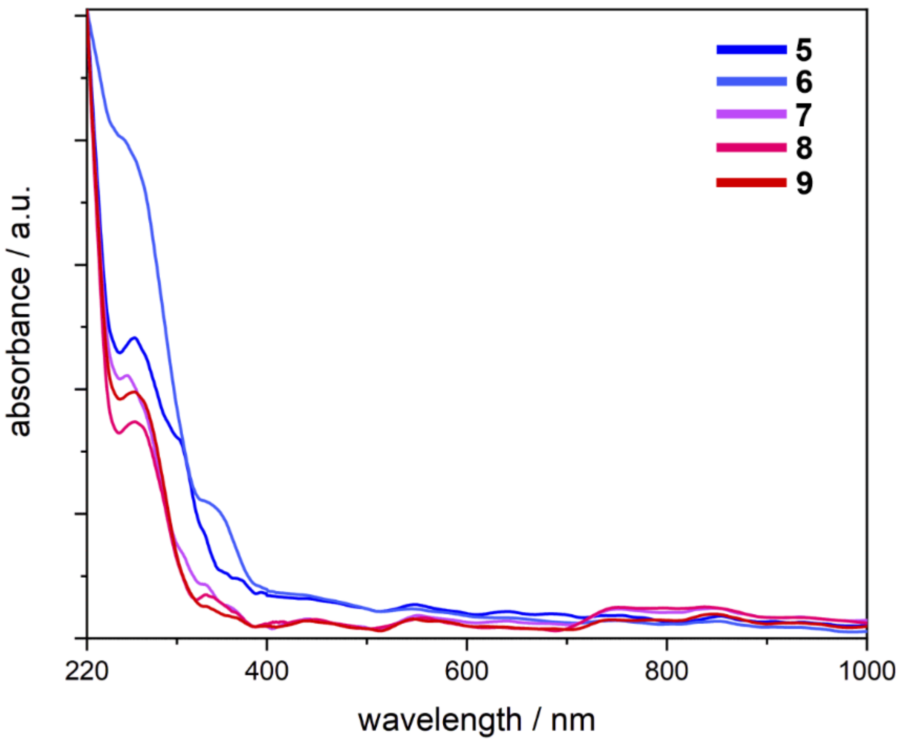

Figure S10. Solid-state room-temperature UV-vis-NIR absorption spectra of **5–9** in the 220–1000 nm range. The spectra were normalized to the intensity at 220 nm.

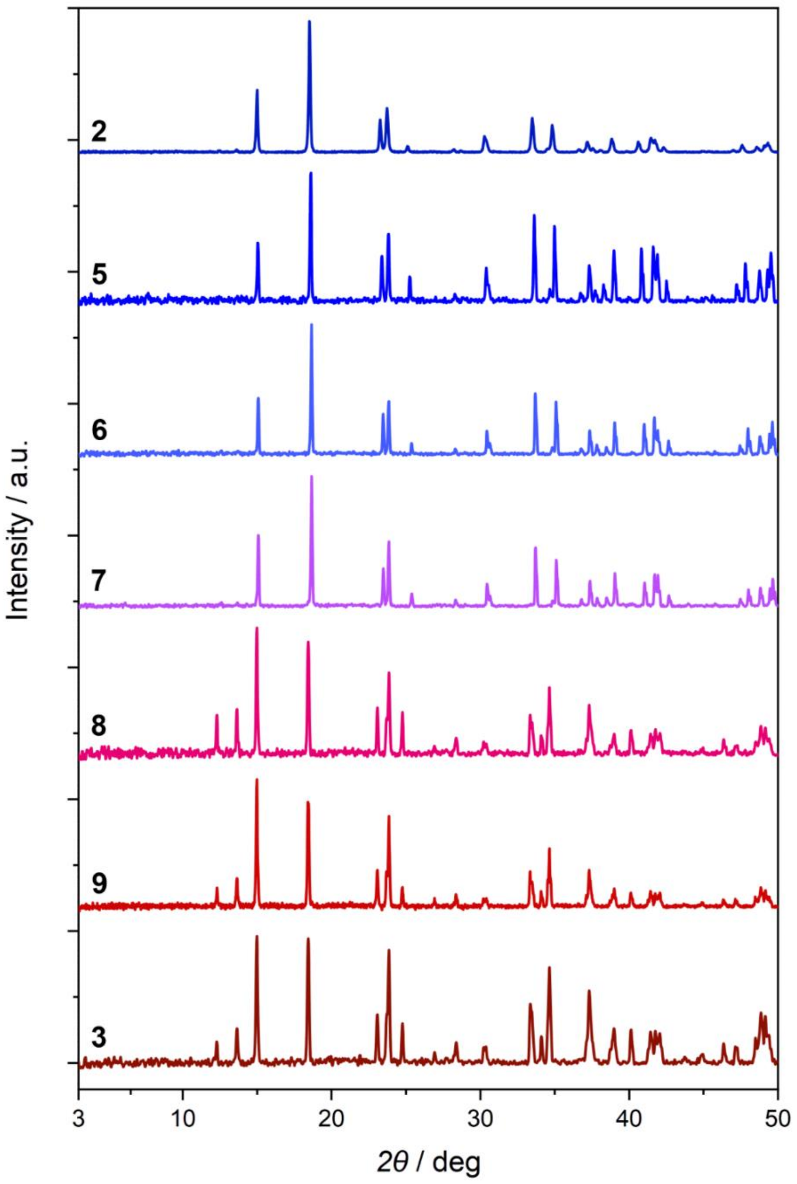

Figure S11. Experimental powder X-ray diffraction (P-XRD) patterns of the polycrystalline samples of **5–9**, compared with the experimental P-XRD patterns of **2** (top, a hexagonal phase, Table S1, Figure 1) and **3** (bottom, an orthorhombic phase, Table S1, Figure 1).

Table S5. Comparison of the unit cell parameters obtained from the room-temperature P-XRD experiments for **2**, **5**, **6**, **7**, **8**, **9**, and **3** (Figure S11), adopting the $P6_3$/m (compounds **2** and **5–7**) and *C*mcm (compounds **8–9** and **3**) space groups and using the LeBail fitting procedure performed with an EXPO 2014 software.[S7]

compound	*a* / Å	*b* / Å	*c* / Å	*V* / Å^3
**2**	7.4840(7)	7.4840(7)	14.157(2)	686.69(14)
**5**	7.4666(6)	7.4666(6)	14.1005(15)	680.79(11)
**6**	7.4602(5)	7.4602(5)	14.0311(15)	676.27(10)
**7**	7.4570(6)	7.4570(6)	14.0250(13)	675.41(10)
**8**	7.501(2)	12.884(2)	14.373(4)	1389.1(7)
**9**	7.5043(11)	12.887(2)	14.374(3)	1390.0(5)
**3**	7.5023(15)	12.885(2)	14.370(3)	1389.1(5)

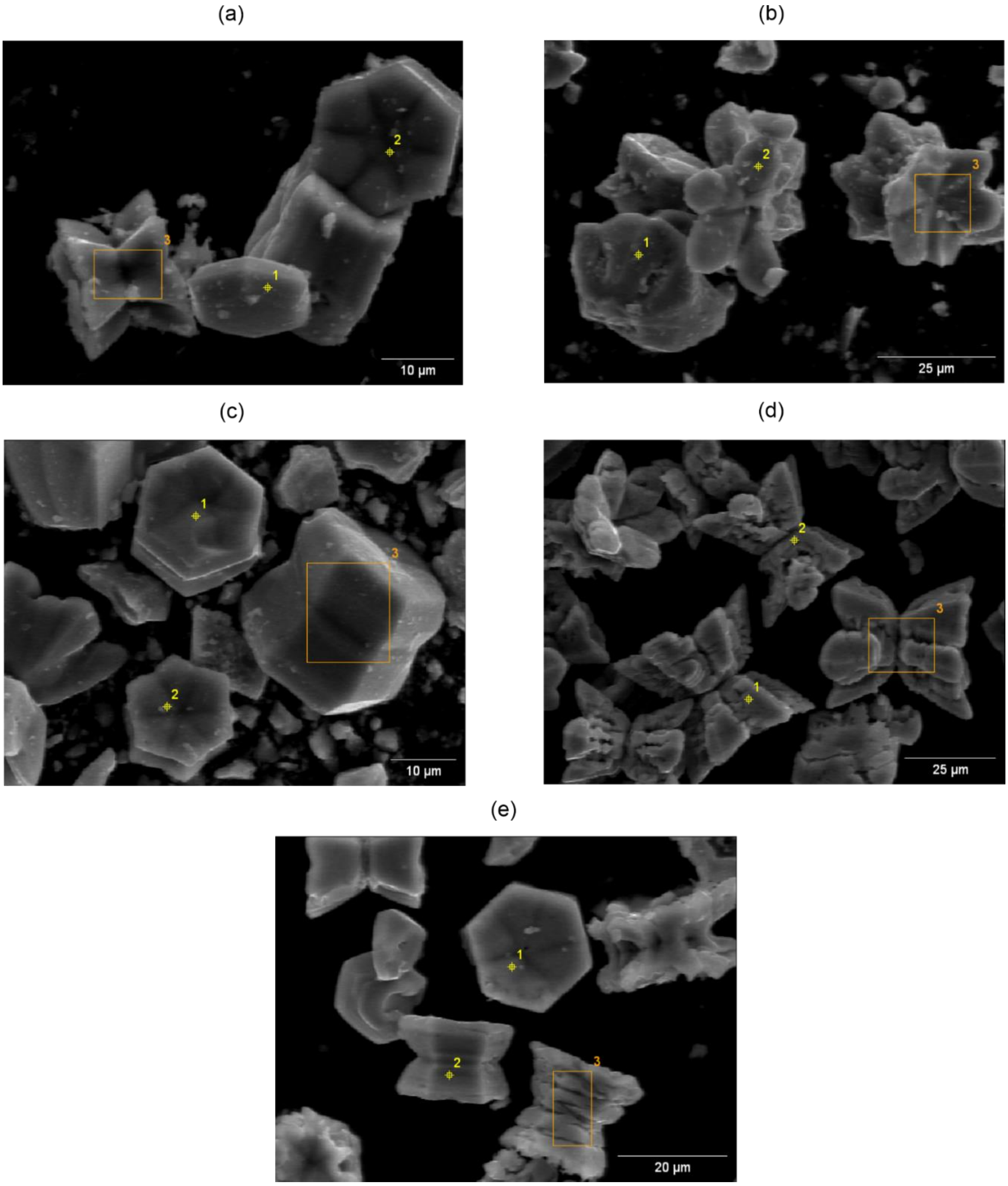

Figure S12. Representative SEM images of the microcrystals of compounds **5** (a), **6** (b), **7** (c), **8** (d), and **9** (e). The labeled points and areas correspond to the measurement points of the SEM EDXMA microanalysis. The results for the indicated points/areas as well as for the other investigated places of a few different crystals are gathered in Table S6.

Table S6. Results of the SEM EDXMA microanalysis of the lanthanide ions' compositions, expressed as the Ce/Sm ratio, in compounds **5–9**. The representative measurements points/areas 1–3 are shown in Figure S12. It is important to note that for compound **9** two measurement points revealed the Ce/Sm ratio below the reliable detection limit of 0.001. The peaks related to the Ce atoms were observed but they were too small be reliably determined; the values can be estimated to be close to 0.001. These very small values of Ce/Sm ratio correspond to two measurements points while all measurement areas (a few selected areas instead of single points) always showed the higher Ce/Sm ratios; thus, these smaller values are related to the non-ideally homogenous dispersion of the Ce atoms within the different fragments of the crystals.

measurement point or area	**5**	**6**	**7**	**8**	**9**
	**Ce/Sm ratio**				
1	1.19(6)	0.26(2)	0.14(2)	0.011(5)	0.001(4)
2	0.97(5)	0.24(2)	0.13(1)	0.006(5)	below the reliable detection limit (ca. 0.001)
3	0.97(5)	0.23(1)	0.11(2)	0.007(6)	0.009(5)
4	1.00(6)	0.24(2)	0.14(2)	0.014(6)	0.005(4)
5	1.19(7)	0.18(2)	0.15(1)	0.012(6)	0.001(5)
6	1.01(6)	0.25(2)	0.11(1)	0.012(6)	below the reliable detection limit (ca. 0.001)
7	1.21(7)	0.26(2)	0.12(2)	0.028(7)	0.003(5)
8	1.09(11)	0.26(2)	0.16(2)	0.009(7)	0.002(5)
9	1.08(11)	0.20(2)	0.09(2)	0.002(7)	0.001(5)
10	1.38(8)	0.20(2)	0.13(2)	0.001(7)	0.002(5)
Average ratio	1.11(7)	0.23(2)	0.13(1)	0.010(6)	0.002(3)

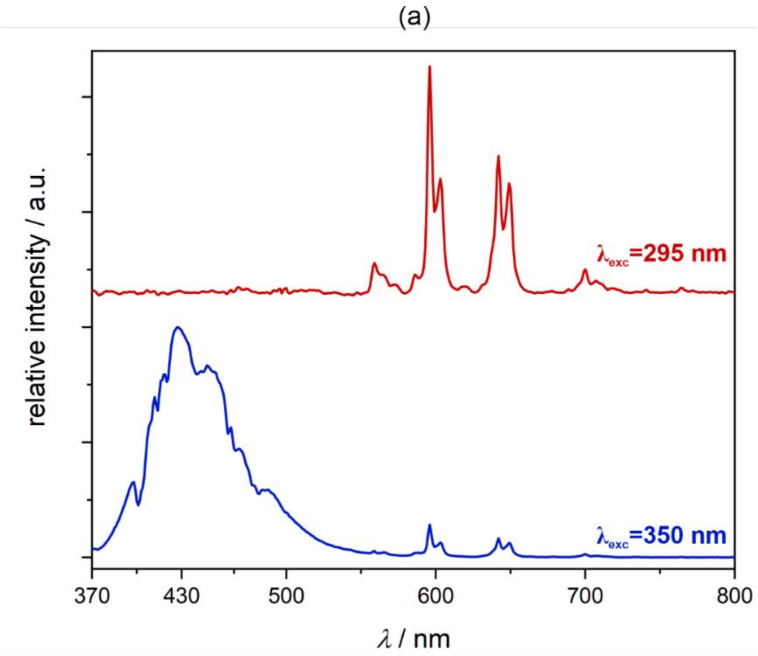

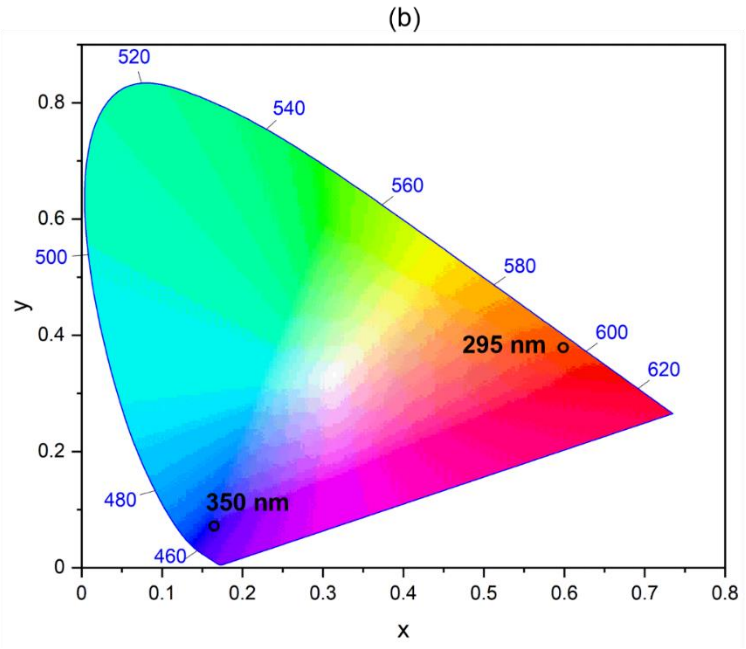

Figure S13. Room-temperature emission spectra of **8** for the indicated excitation wavelengths (a), and the resulting emission colors shown on the CIE 1931 chromaticity diagram (b).

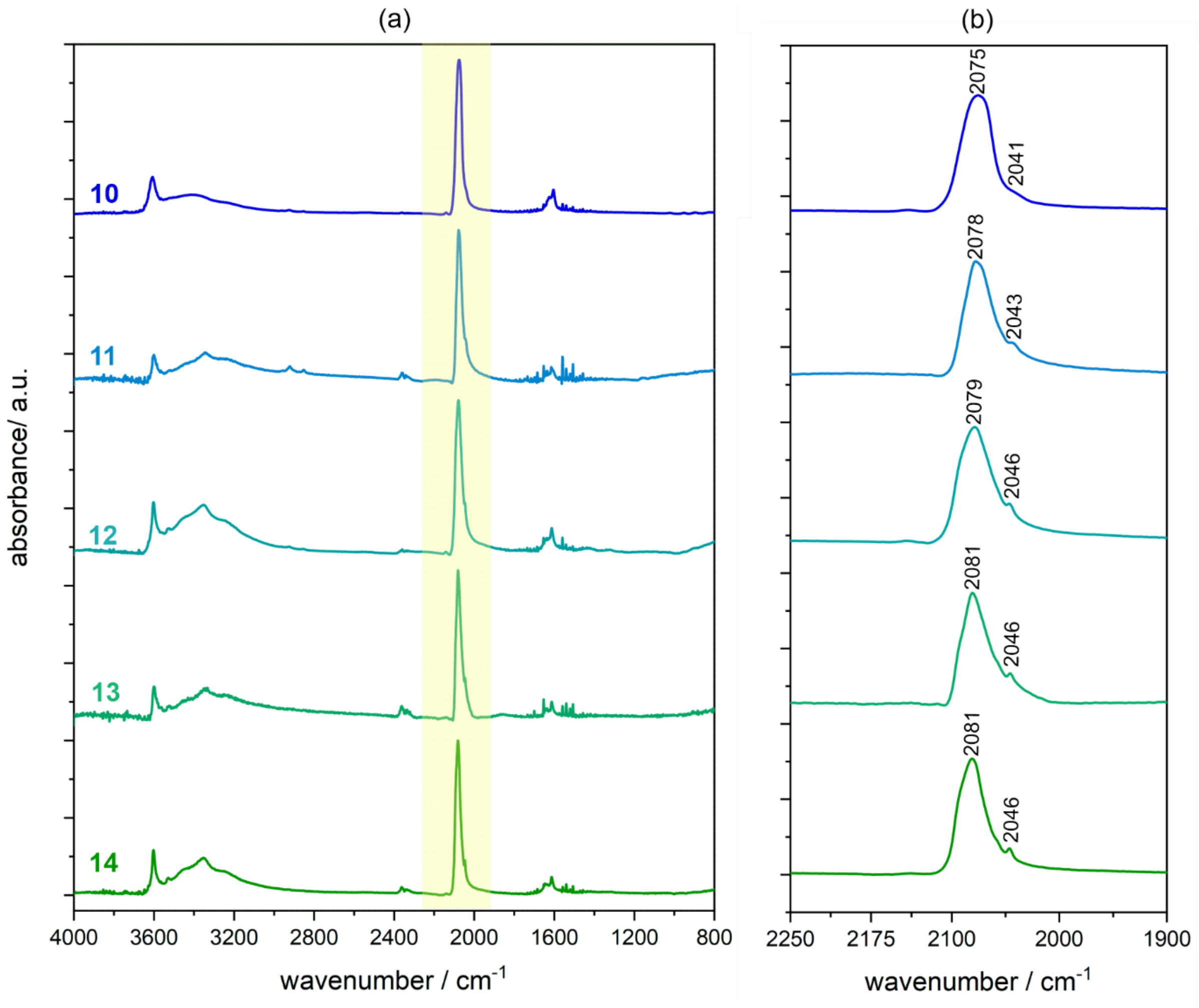

Figure S14. Infrared (IR) absorption spectra of **10–14**, measured in the 4000–800 cm^{-1} range (a), and the enlargement of the 2250–1900 cm^{-1} region (b), related to the stretching vibrations of cyanido ligands within hexacyanido-ruthenate(II) complexes.[S1–S3]

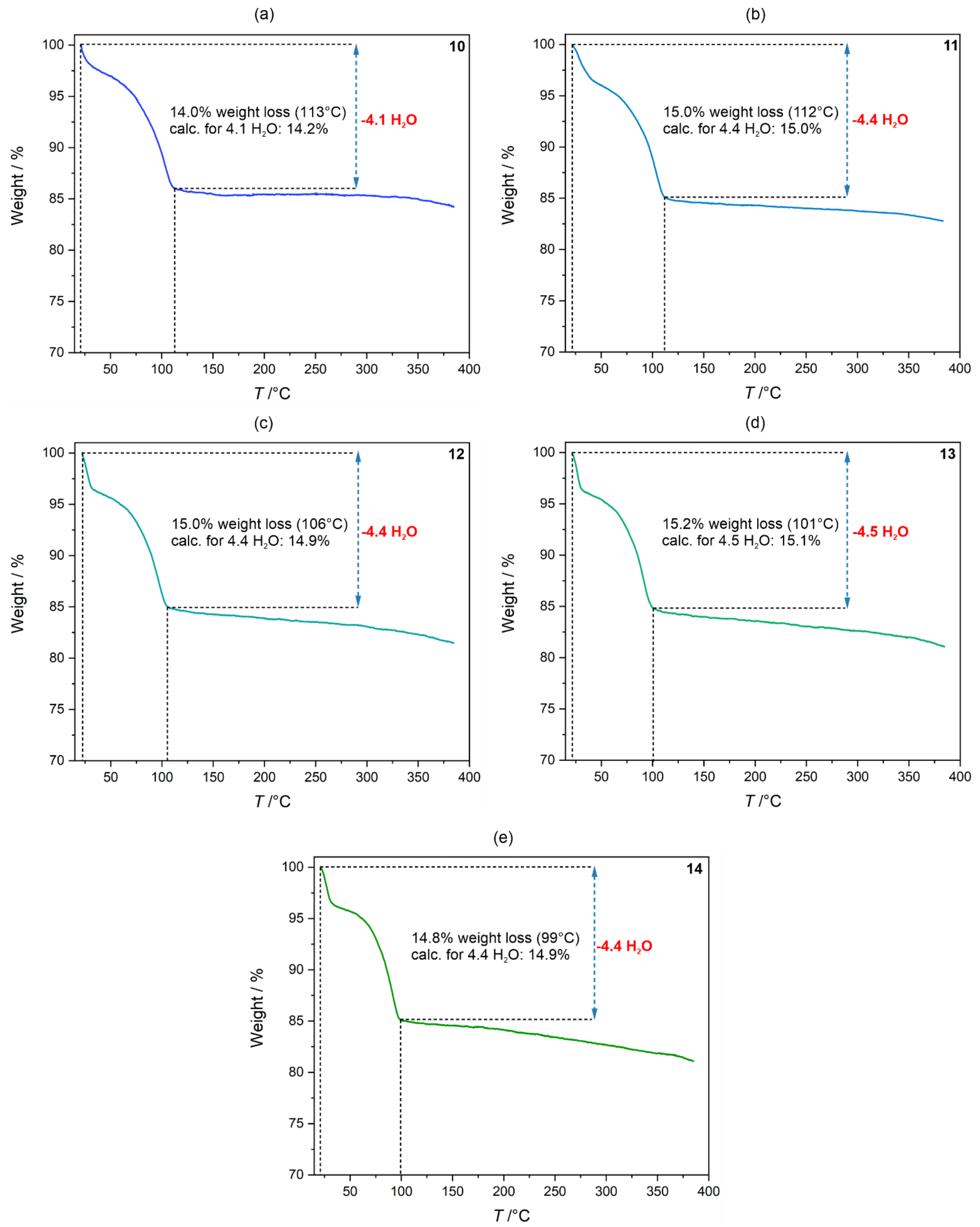

Figure S15. Thermogravimetric (TG) curves of **10** (a), **11** (b), **12** (c), **13** (d), and **14** (e), measured under a nitrogen atmosphere upon the continuous heating with the 1 °C·min^{-1} rate. The step related to the temperature-induced removal of water molecules is indicated on the graph. A few distinguishable steps within the indicated ranges are related to the subsequent removal of non-coordinated (lower temperatures) and coordinated (higher temperatures) water molecules.

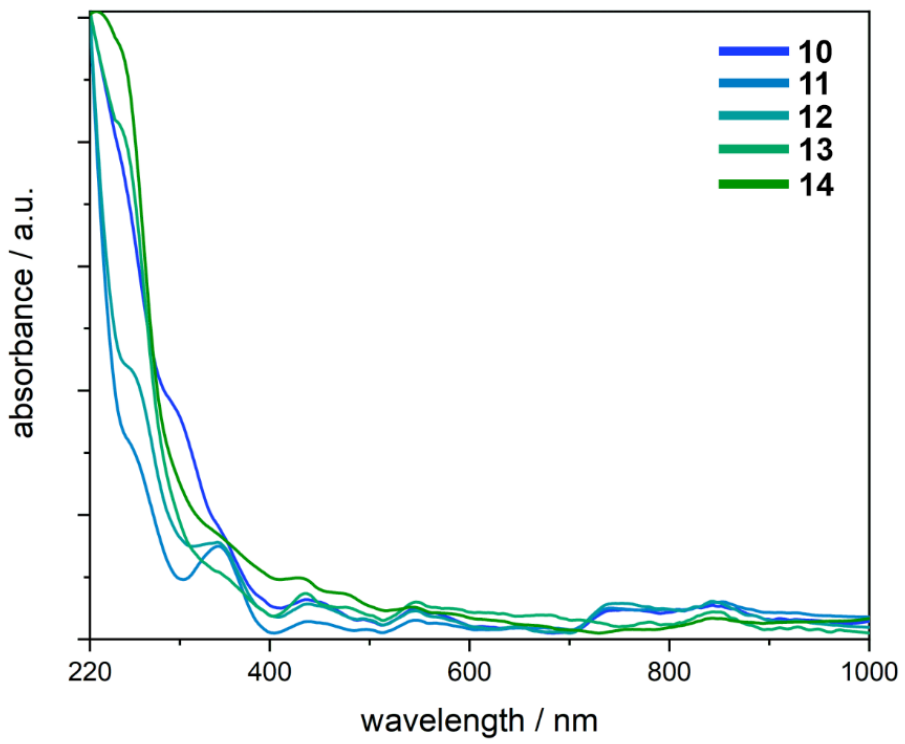

Figure S16. Solid-state room-temperature UV-vis-NIR absorption spectra of **10–14** in the 220–1000 nm range. The spectra were normalized to the intensity at 220 nm.

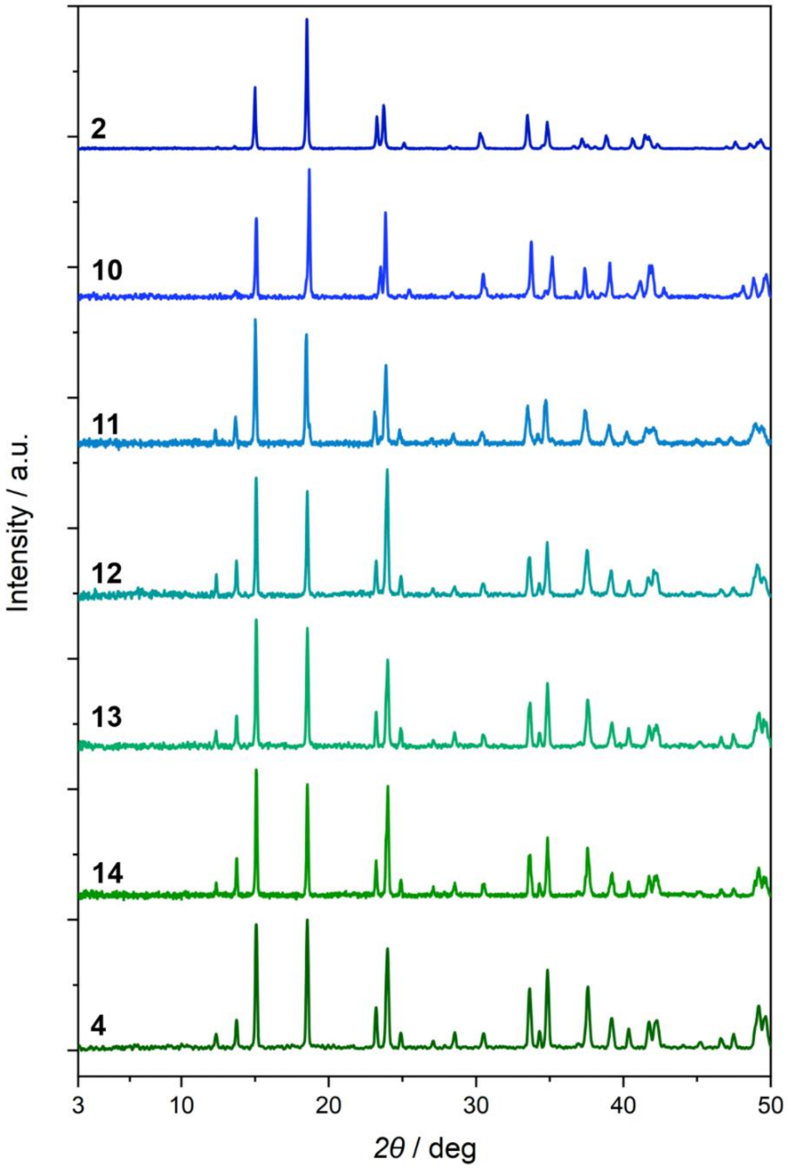

Figure S17. Experimental powder X-ray diffraction (P-XRD) patterns of the polycrystalline samples of **10–14**, compared with the experimental P-XRD patterns of **2** (top, a hexagonal phase, Table S1, Figure 1) and **4** (bottom, an orthorhombic phase, Table S1, Figure 1).

Table S7. Comparison of the unit cell parameters obtained from the room-temperature P-XRD experiments for **2**, **10**, **11**, **12**, **13**, **14**, and **4** (Figure S17), adopting the $P6_3$/m (compounds **2** and **10**) and *C*mcm (compounds **11–14** and **4**) space groups and using the LeBail fitting procedure performed with an EXPO 2014 software.[S7]

compound	*a* / Å	*b* / Å	*c* / Å	*V* / Å^3
**2**	7.4840(7)	7.4840(7)	14.157(2)	686.69(14)
**10**	7.4555(7)	7.4555(7)	13.980(2)	672.98(14)
**11**	7.4462(18)	12.838(6)	14.324(8)	1369.3(11)
**12**	7.437(3)	12.829(3)	14.266(5)	1361.2(8)
**13**	7.4333(19)	12.8091(13)	14.292(2)	1360.7(4)
**14**	7.3933(13)	12.856(2)	14.268(3)	1356.2(4)
**4**	7.403(2)	12.789(5)	14.275(4)	1351.5(7)

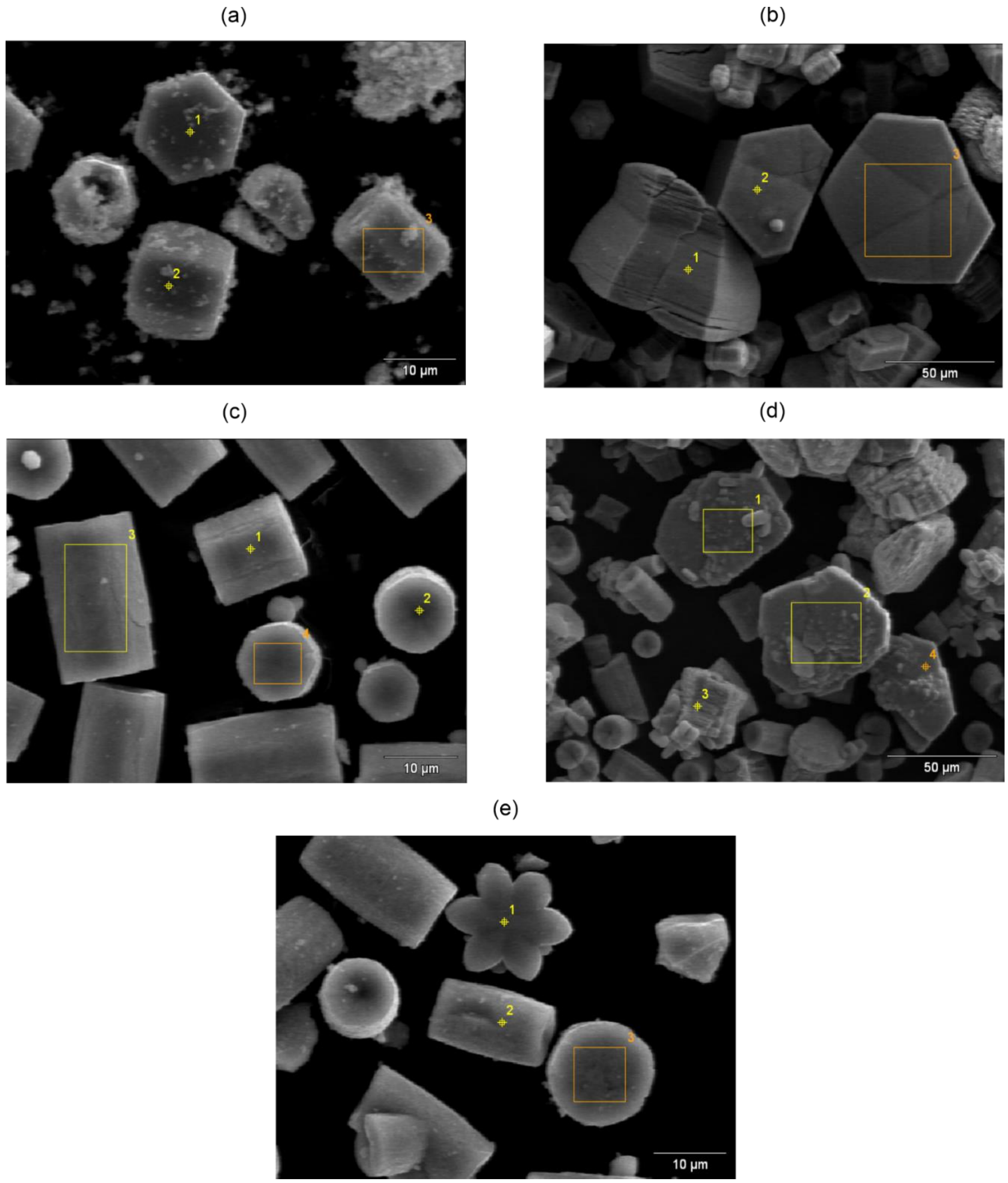

Figure S18. Representative SEM images of the microcrystals of compounds **10** (a), **11** (b), **12** (c), **13** (d), and **14** (e). The labeled points and areas correspond to the measurement points of the SEM EDXMA microanalysis. The results for the indicated points/areas as well as for the other investigated places of a few different crystals are gathered in Table S8.

Table S8. Results of the SEM EDXMA microanalysis of the lanthanide ions' compositions, expressed as the Ce/Tb ratio, in compounds **10–14**. The representative measurements points/areas 1–3 are shown in Figure S18. It is important to note that for compound **14** a few measurement points revealed the Ce/Tb ratio below the reliable detection limit of 0.001. The peaks related to the Ce atoms were observed but they were too small be reliably determined; the values can be estimated to be close to 0.001. These very small values of Ce/Tb ratio correspond to some measurements points while all measurement areas (a few selected areas instead of single points) always showed the higher Ce/Tb ratios; thus, these smaller values are related to the non-ideally homogenous dispersion of the Ce atoms within the different fragments of the crystals.

measurement point or area	**10**	**11**	**12**	**13**	**14**
	**Ce/Tb ratio**				
1	0.93(7)	0.49(3)	0.072(8)	0.012(6)	0.005(6)
2	1.23(6)	0.48(3)	0.105(9)	0.018(6)	below the reliable detection limit (ca. 0.001)
3	0.82(5)	0.61(4)	0.086(14)	0.002(6)	0.010(7)
4	0.79(5)	0.46(3)	0.067(8)	0.011(6)	0.001(6)
5	0.56(3)	0.58(3)	0.093(9)	0.022(7)	0.003(6)
6	0.69(4)	0.40(3)	0.052(11)	0.015(6)	0.006(6)
7	0.98(6)	0.54(3)	0.072(13)	0.016(6)	below the reliable detection limit (ca. 0.001)
8	0.95(6)	0.70(4)	0.065(7)	0.015(6)	below the reliable detection limit (ca. 0.001)
9	0.53(3)	0.79(4)	0.068(12)	0.012(6)	0.004(6)
10	0.47(3)	0.46(3)	0.103(14)	0.018(6)	0.004(6)
Average ratio	0.80 (5)	0.55(3)	0.078(11)	0.014(6)	0.003(4)

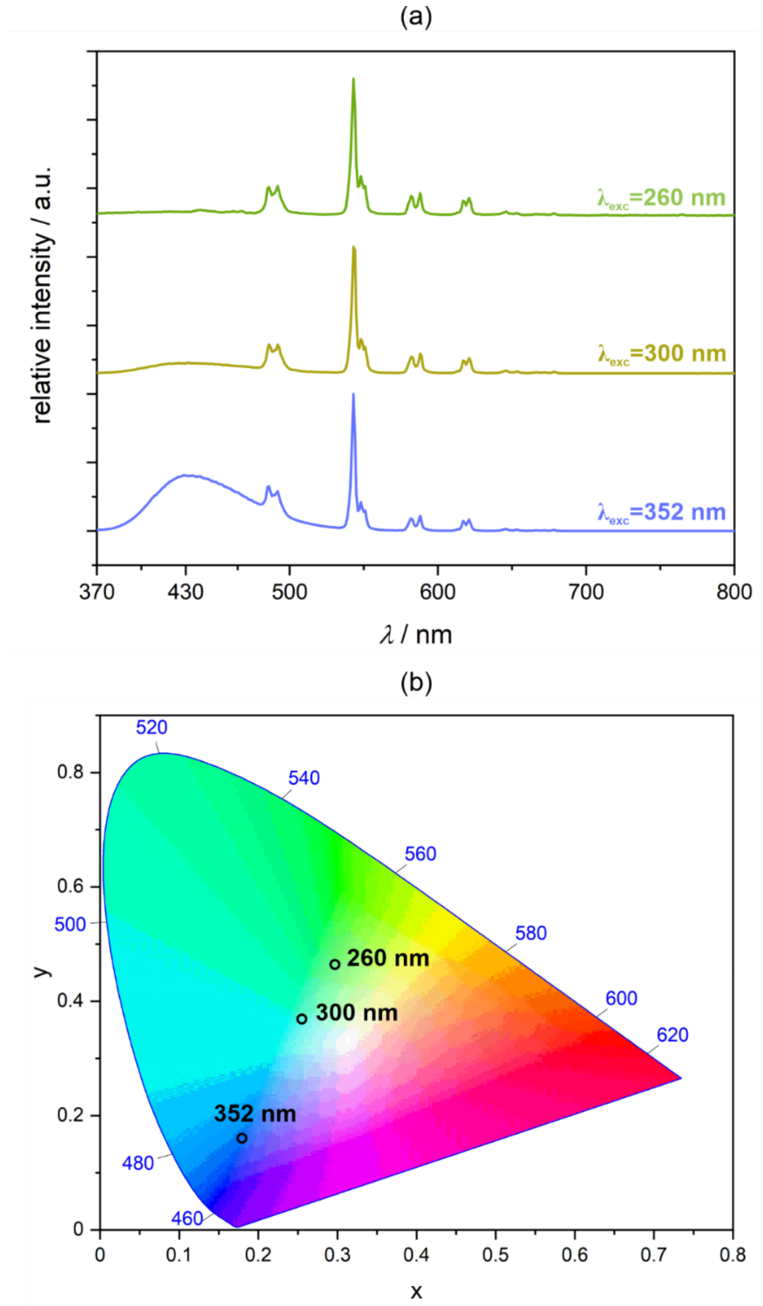

Figure S19. Room-temperature emission spectra of **13** for the indicated excitation wavelengths (a), and the resulting emission colors shown on the CIE 1931 chromaticity diagram (b).

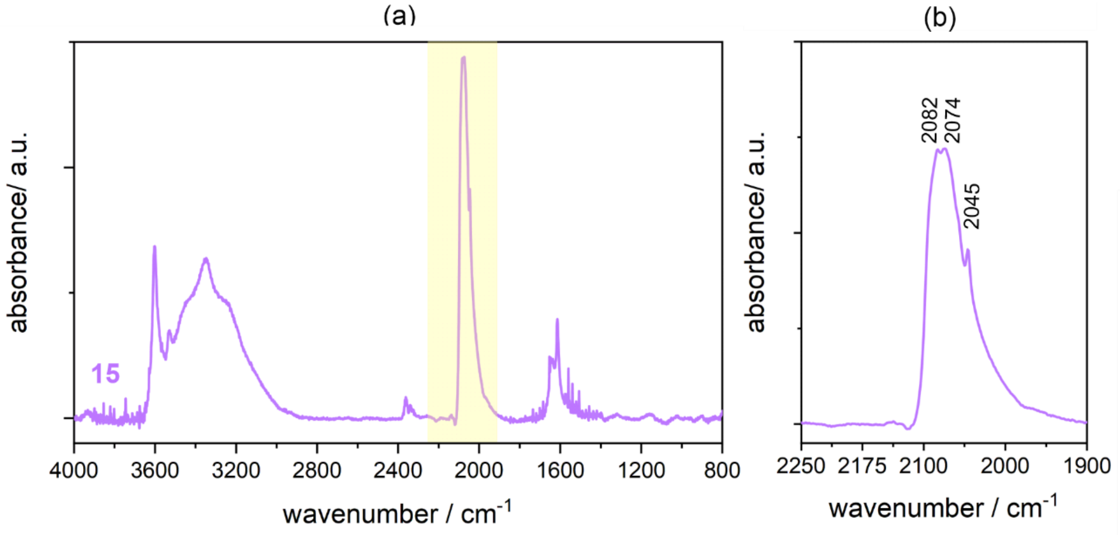

Figure S20. Infrared (IR) absorption spectrum of **15**, measured in the 4000–800 cm^{-1} range (a), and the enlargement of the 2250–1900 cm^{-1} region (b), related to the stretching vibrations of cyanido ligands within hexacyanidoruthenate(II) complexes.[S1–S3]

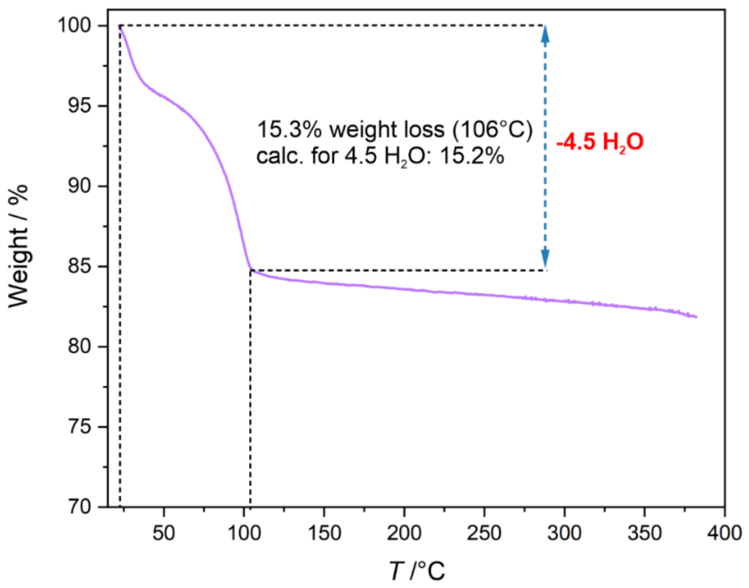

Figure S21. Thermogravimetric (TG) curve of **15**, measured under a nitrogen atmosphere upon the continuous heating with the 1 °C·min^{-1} rate. The step related to the temperature-induced removal of water molecules is indicated on the graph. A few distinguishable steps within the indicated ranges are related to the subsequent removal of non-coordinated (lower temperatures) and coordinated (higher temperatures) water molecules.

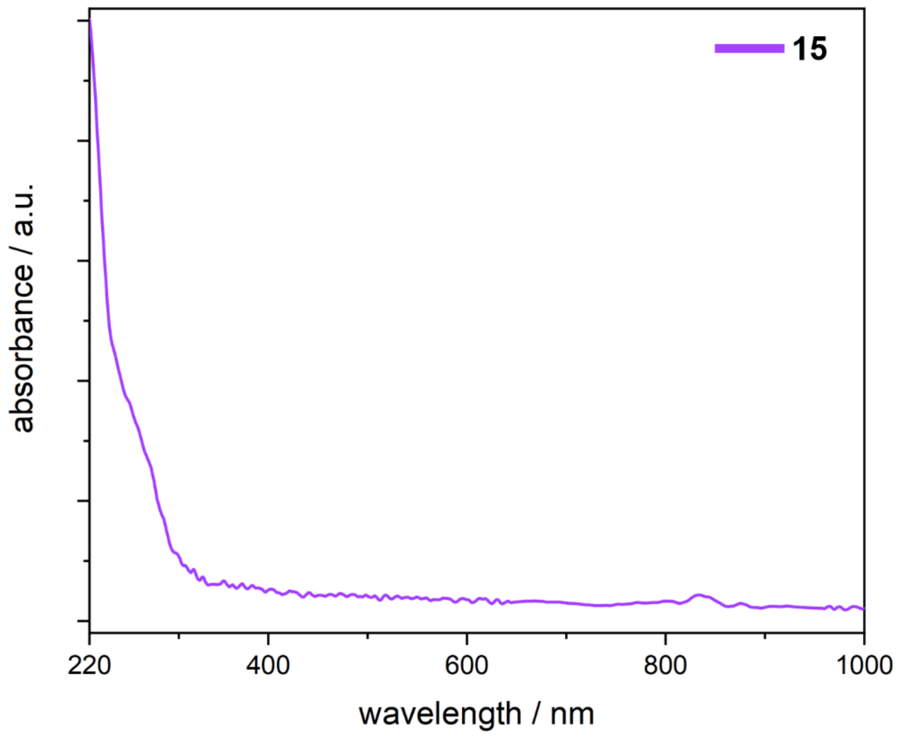

Figure S22. Solid-state room-temperature UV-vis-NIR absorption spectrum of **15** in the 220–1000 nm range. The spectrum was normalized to the intensity at 220 nm.

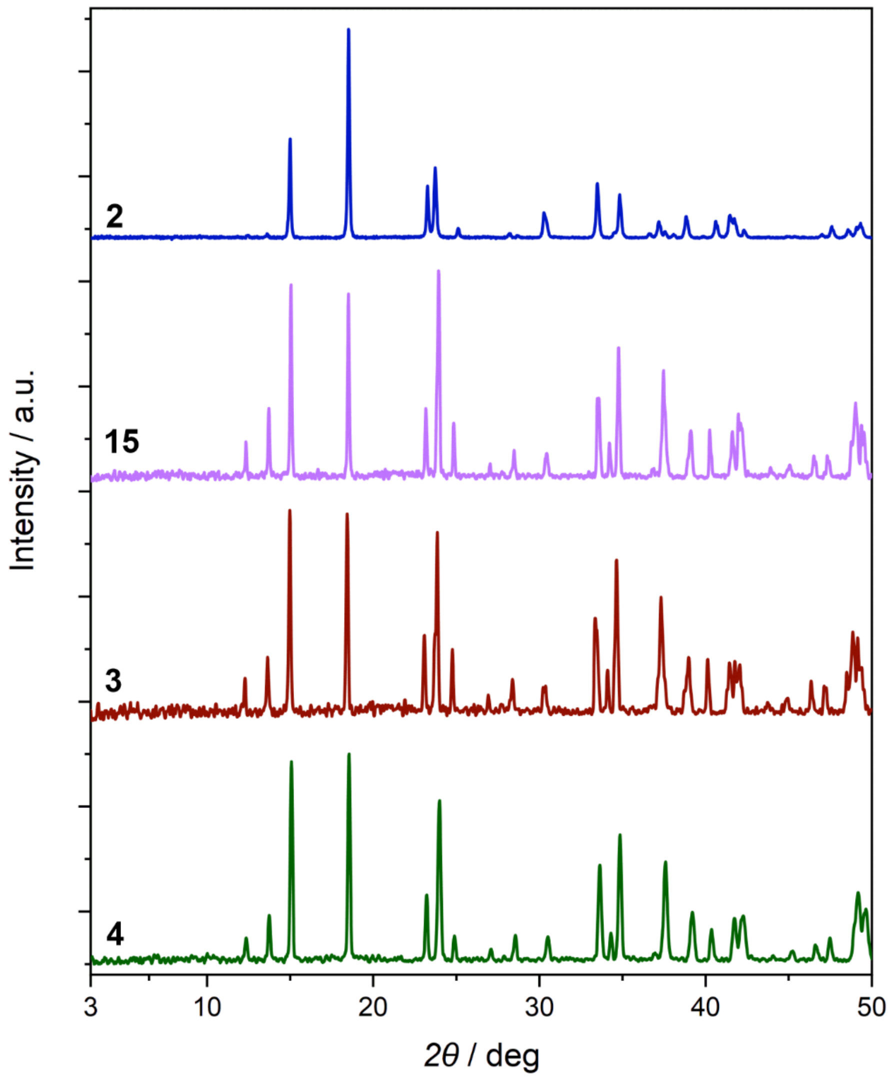

Figure S23. Experimental powder X-ray diffraction (P-XRD) pattern of the polycrystalline sample of **15**, compared with the experimental P-XRD patterns of **2** (top, a hexagonal phase, Table S1, Figure 1) as well as **3** and **4** (bottom, orthorhombic phases, Table S1, Figure 1).

Table S9. Comparison of the unit cell parameters obtained from the room-temperature P-XRD experiments for **2**, **15**, **3**, and **4** (Figure S23), adopting the $P6_3$/m (compound **2**) and *C*mcm (compounds **15**, **3**, and **4**) space groups and using the LeBail fitting procedure performed with an EXPO 2014 software.[S7]

compound	*a* / Å	*b* / Å	*c* / Å	*V* / Å^3
**2**	7.4840(7)	7.4840(7)	14.157(2)	686.69(14)
**15**	7.456(2)	12.839(5)	14.317(6)	1370.5(9)
**3**	7.5023(15)	12.885(2)	14.370(3)	1389.1(5)
**4**	7.403(2)	12.789(5)	14.275(4)	1351.5(7)

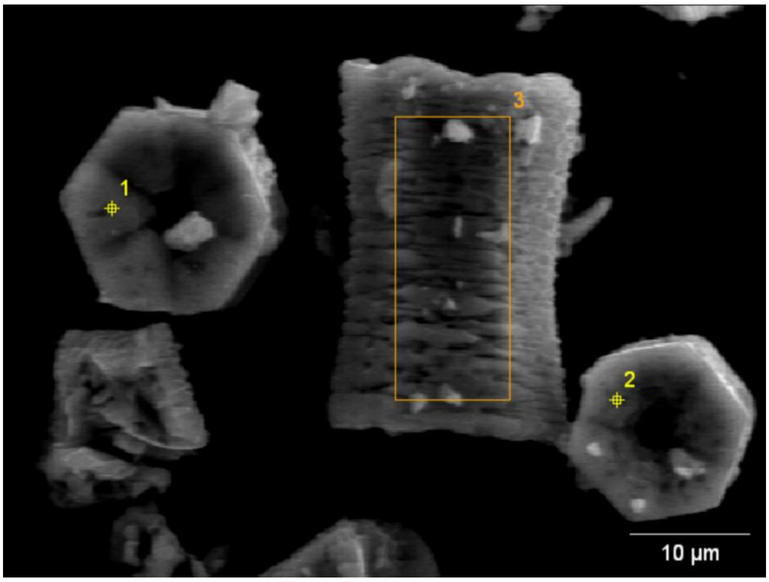

Figure S24. Representative SEM images of the microcrystals of compounds **15**. The labeled points and areas correspond to the measurement points of the SEM EDXMA microanalysis. The results for the indicated points/areas as well as for the other investigated places of a few different crystals are gathered in Table S10.

Table S10. Results of the SEM EDXMA microanalysis of the lanthanide ions' composition, expressed as the Ce/Sm and Ce/Tb ratios, in **15**. Note that only five measurements points are here presented with the reliable values while ten measurements points were shown for compounds **5–14** (Tables S6 and S8, Figure S24). The other five points were measured for **15** but they showed the Ce/Sm and Ce/Tb ratios smaller than 0.001 which was below the reliable detection limit of the used apparatus. However, they represent the dispersion of the Ce atoms within the crystals; thus, these values were used for the calculation of the average ratios (*). These very small values of Ce/Sm and Ce/Tb correspond to some measurements points while all measurement areas (a few selected areas instead of single points) always showed the higher Ce/Sm and Ce/Tb ratios; thus, these smaller values are related to the non-ideally homogenous dispersion of the Ce atoms within the different fragments of the crystals.

measurement point or area	**15**	
	**Ce/Sm ratio**	**Ce/Tb ratio**
1	0.005(11)	0.004(8)
2	0.006(10)	0.005(8)
3	0.001(10)	0.001(8)
4	0.008(11)	0.006(7)
5	0.004(11)	0.003(8)
6–10	non-zero values but below the reliable detection limit of 0.001* (see note in the caption)	
Average ratio*	0.003(5)	0.002(4)

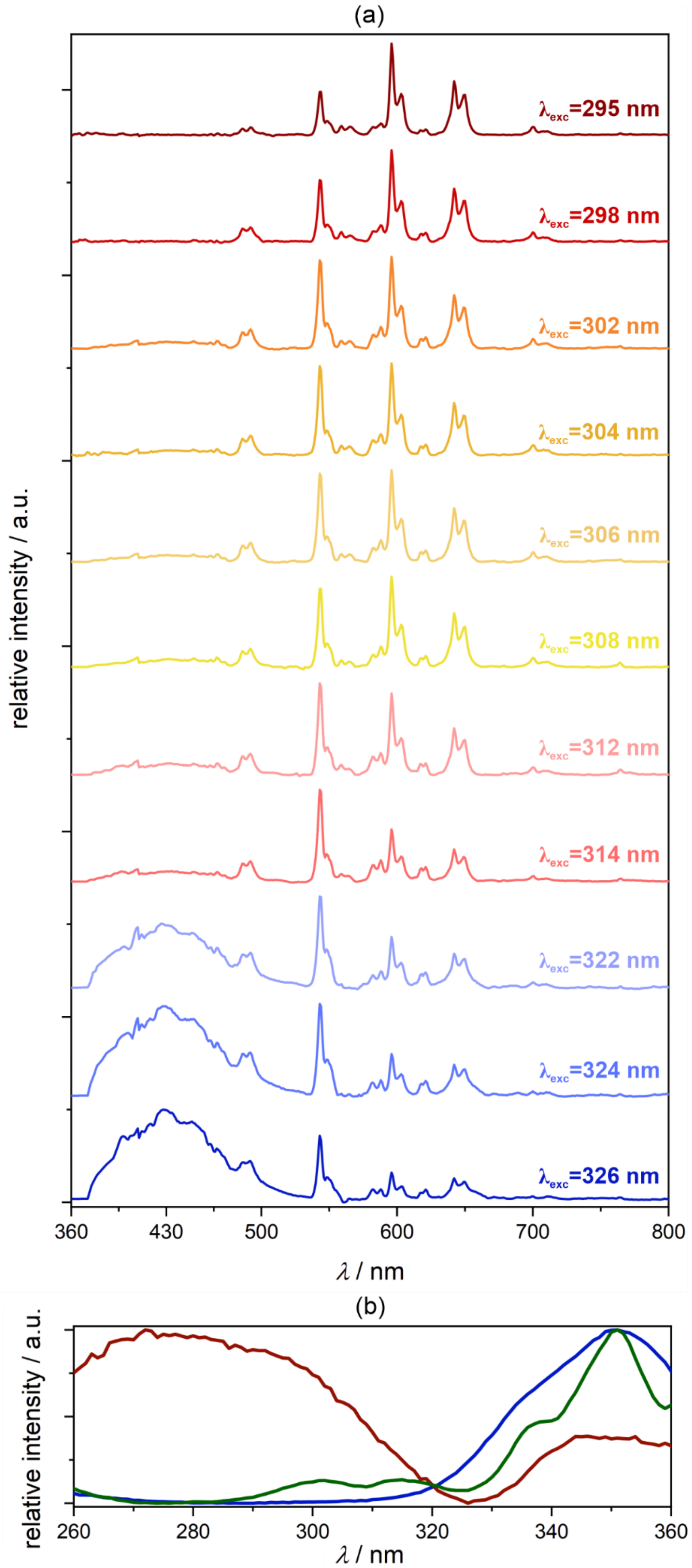

Figure S25. Additional room-temperature emission spectra of **15** for the indicated excitation wavelengths (a) and the excitation spectra (b) for the monitored emission maxima at 596 nm (red line), 543 nm (green line), and 427 nm (blue line).

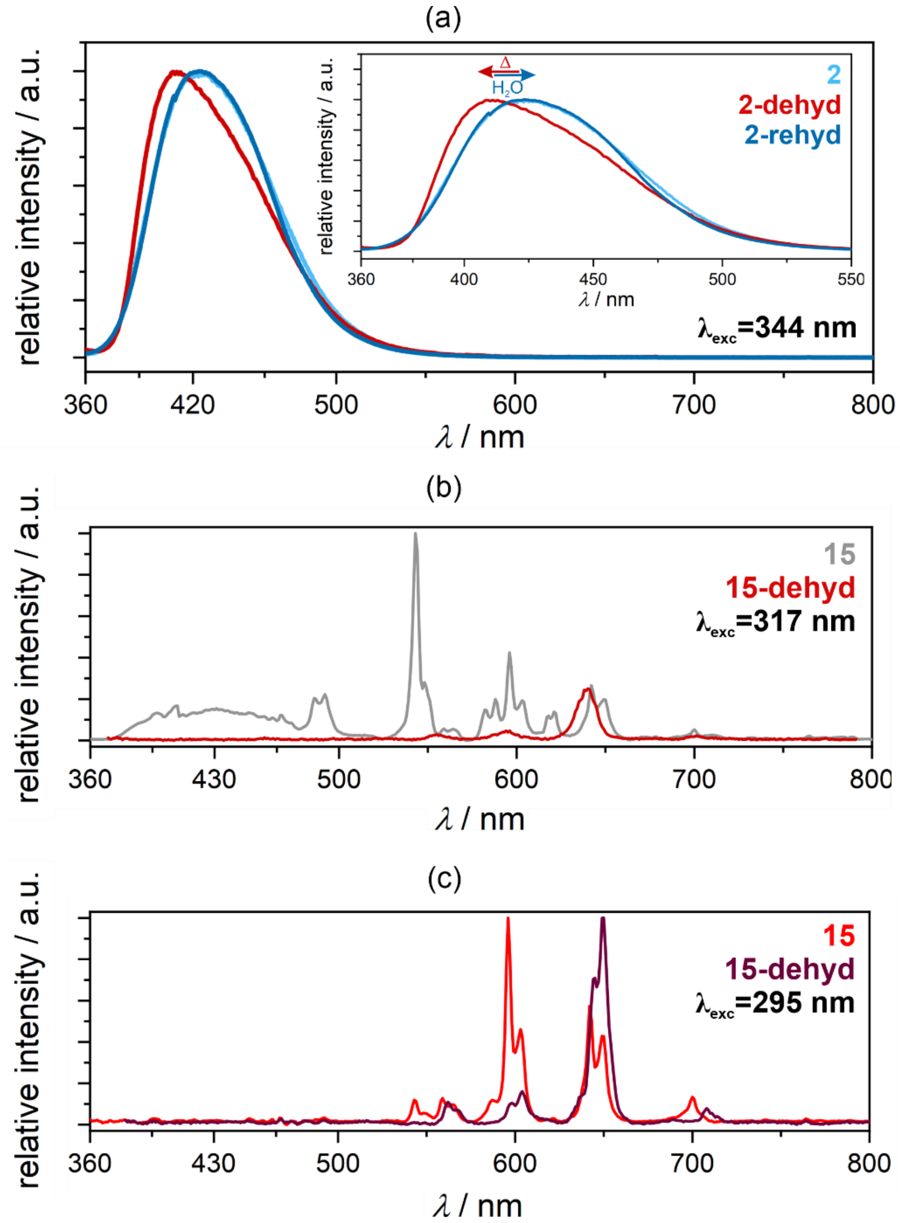

Figure S26. Optical characteristics after thermal dehydration of the polycrystalline samples of **2** and **15**: emission spectrum for **2** under the indicated excitation, compared with the analogous spectra for the dehydrated form od **2** (**2-dehyd**) and its rehydrated form (**2-rehyd**) (a), emission spectra for **15** and its thermally dehydrated form (**15-dehyd**), both for the indicated excitation of 317 nm, (b), and emission spectra for **15** and its thermally dehydrated form (**15-dehyd**), both for the indicated excitation of 295 nm (c). Note that all spectra were normalized to the selected peaks occurring for both hydrated as well as dehydrated phases. The real emission intensity after dehydration was much weaker than for the as-synthesized sample for the case of compound **15** while the dehydration only weakly affects the intensity for the case of compound **2**.

Table S11. Summary of the *x* and *y* CIE 1931 chromaticity parameters for the emissions colors of **1–15** at indicated excitation wavelengths.

**compound** (excitation)	***x***	***y***	**compound** (excitation)	***x***	***y***	**compound** (excitation)	***x***	***y***
**1** (λ_{exc}=280 nm)	0.200	0.229	**12** (λ_{exc}=349 nm)	0.172	0.136	**15** (λ_{exc}=300 nm)	0.462	0.429
**2** (λ_{exc}=313 nm)	0.153	0.053	**13** (λ_{exc}=260 nm)	0.297	0.465	**15** (λ_{exc}=302 nm)	0.451	0.418
**3** (λ_{exc}=292 nm)	0.538	0.387	**13** (λ_{exc}=300 nm)	0.255	0.369	**15** (λ_{exc}=304 nm)	0.433	0.406
**4** (λ_{exc}=370 nm)	0.290	0.559	**13** (λ_{exc}=352 nm)	0.179	0.161	**15** (λ_{exc}=306 nm)	0.426	0.383
**5** (λ_{exc}=312 nm)	0.158	0.058	**14** (λ_{exc}=300 nm)	0.315	0.544	**15** (λ_{exc}=308 nm)	0.412	0.360
**6** (λ_{exc}=351 nm)	0.161	0.062	**14** (λ_{exc}=320 nm)	0.306	0.509	**15** (λ_{exc}=310 nm)	0.407	0.344
**7** (λ_{exc}=349 nm)	0.163	0.068	**14** (λ_{exc}=321 nm)	0.299	0.487	**15** (λ_{exc}=312 nm)	0.385	0.353
**8** (λ_{exc}=295 nm)	0.599	0.379	**14** (λ_{exc}=321.5 nm)	0.289	0.454	**15** (λ_{exc}=314 nm)	0.369	0.358
**8** (λ_{exc}=350 nm)	0.165	0.072	**14** (λ_{exc}=322 nm)	0.281	0.427	**15** (λ_{exc}=315 nm)	0.348	0.362
**9** (λ_{exc}=295 nm)	0.512	0.347	**14** (λ_{exc}=322.5 nm)	0.270	0.397	**15** (λ_{exc}=316 nm)	0.338	0.349
**9** (λ_{exc}=305 nm)	0.469	0.315	**14** (λ_{exc}=323 nm)	0.260	0.366	**15** (λ_{exc}=317 nm)	0.325	0.333
**9** (λ_{exc}=320 nm)	0.435	0.285	**14** (λ_{exc}=323.5 nm)	0.246	0.327	**15** (λ_{exc}=318 nm)	0.309	0.305
**9** (λ_{exc}=325 nm)	0.329	0.206	**14** (λ_{exc}=324 nm)	0.235	0.292	**15** (λ_{exc}=319 nm)	0.289	0.270
**9** (λ_{exc}=330 nm)	0.220	0.125	**14** (λ_{exc}=330 nm)	0.212	0.239	**15** (λ_{exc}=320 nm)	0.268	0.232
**9** (λ_{exc}=335 nm)	0.189	0.097	**14** (λ_{exc}=350 nm)	0.202	0.225	**15** (λ_{exc}=322 nm)	0.244	0.188
**9** (λ_{exc}=350 nm)	0.180	0.081	**15** (λ_{exc}=285 nm)	0.536	0.388	**15** (λ_{exc}=324 nm)	0.213	0.146
**10** (λ_{exc}=314 nm)	0.165	0.097	**15** (λ_{exc}=295 nm)	0.502	0.423	**15** (λ_{exc}=326 nm)	0.206	0.137
**11** (λ_{exc}=356 nm)	0.168	0.117	**15** (λ_{exc}=298 nm)	0.484	0.429	**15** (λ_{exc}=330 nm)	0.193	0.117

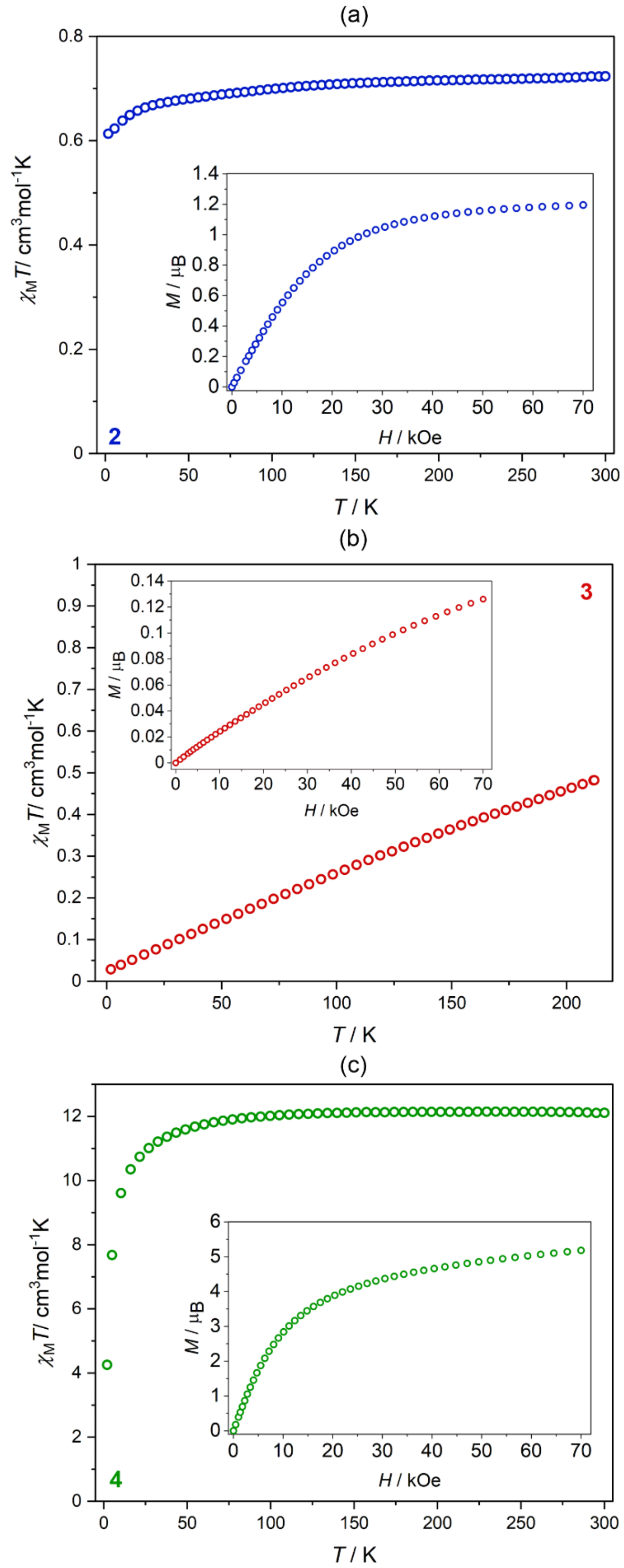

Figure S27. Direct-current (*dc*) magnetic properties of **2** (a), **3** (b), and **4** (d), including the temperature dependences of the magnetic susceptibility–temperature product, $\chi_M T$ at H_{dc} = 1 kOe (the *T*-range of 1.8–300 K for **2** and **4**, while the limited *T*-range of 1.8–200 K for **3** due to the very low signal from the Sm^{III} complexes at higher temperatures), and the field dependences of the molar magnetization at 1.8 K gathered in the 0–70 kOe range.

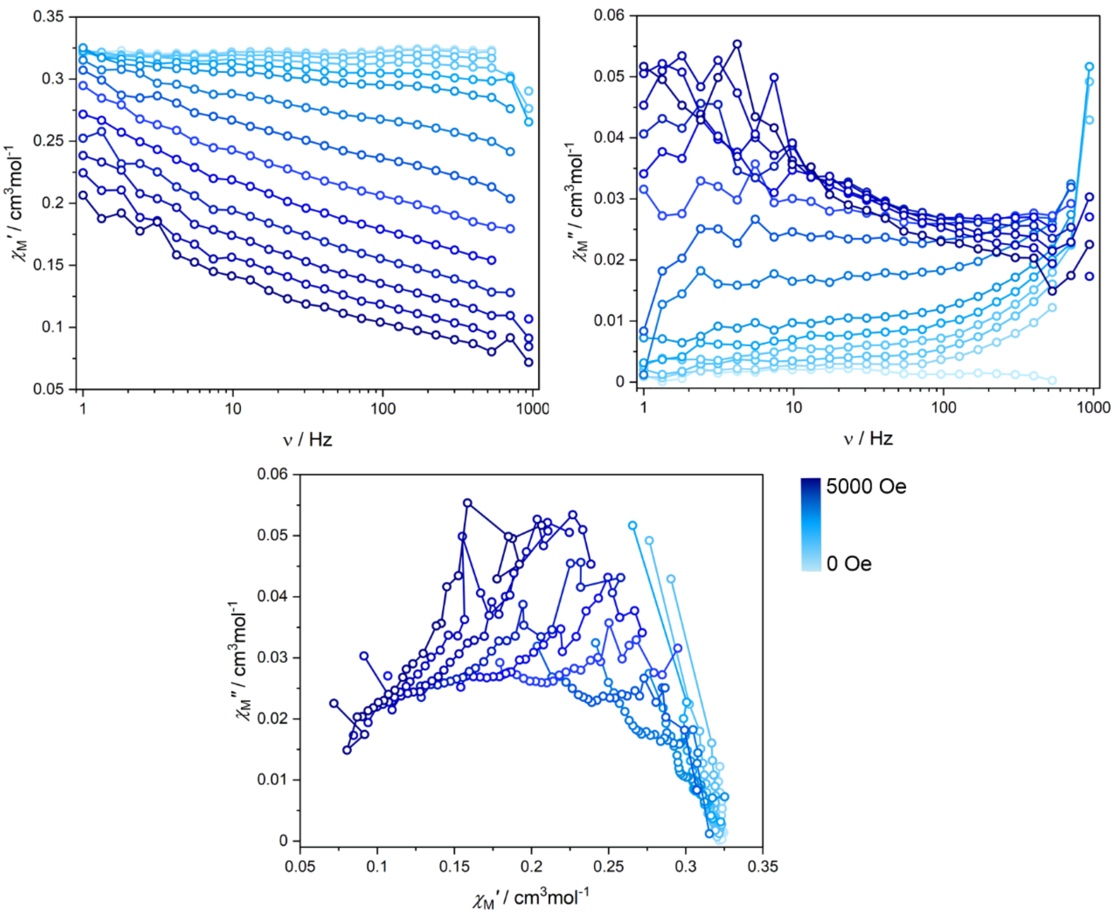

Figure S28. Alternate-current (*ac*) magnetic characteristics of **2**, including the frequency dependences of the in-phase magnetic susceptibility, $\chi_M'(\nu)$, the out-of-phase magnetic susceptibility, $\chi_M''(\nu)$, and the related Argand plot, $\chi_M''(\chi_M')$, under the variable *dc* magnetic field from the 0–5000 Oe range at $T = 1.8$ K. The lines are only to guide the eye.

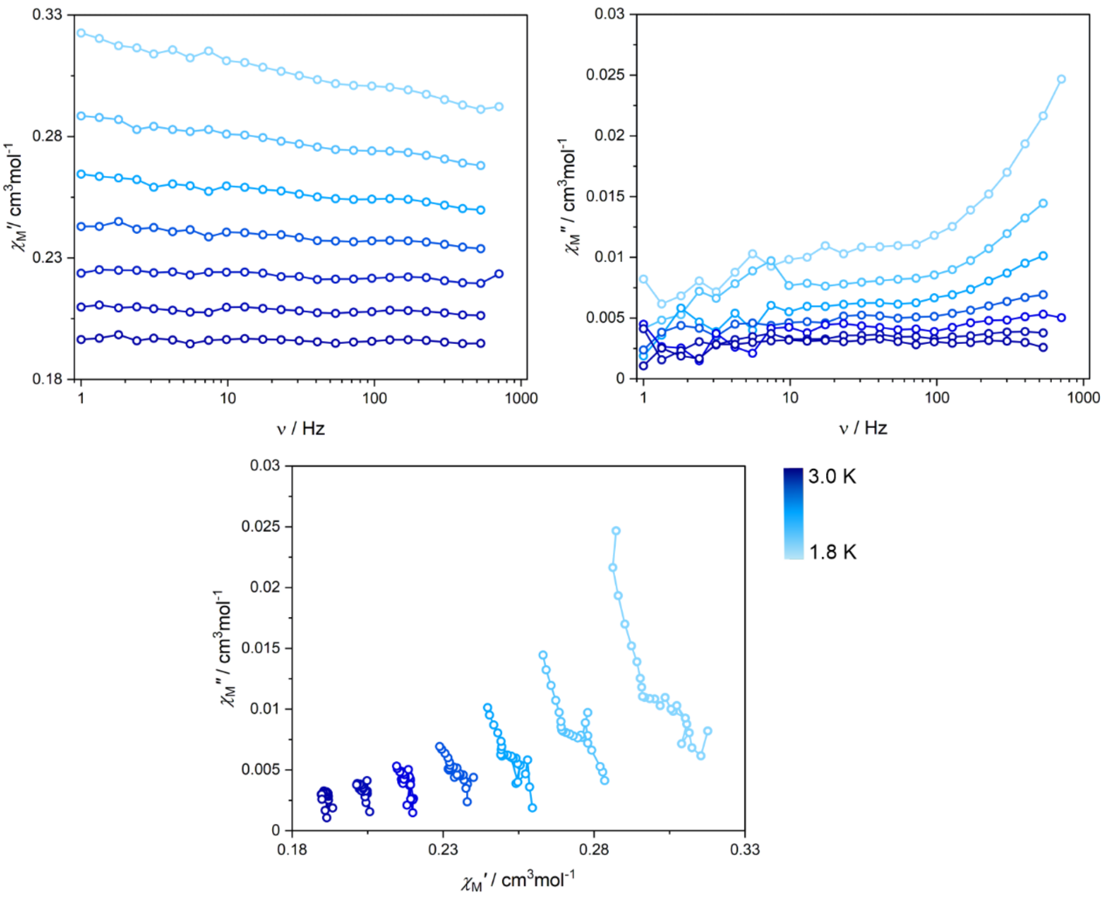

Figure S29. Alternate-current (*ac*) magnetic characteristics of **2**, including the frequency dependences of the in-phase magnetic susceptibility, $\chi_M'(\nu)$, the out-of-phase magnetic susceptibility, $\chi_M''(\nu)$, and the related Argand plot, $\chi_M''(\chi_M')$, under the variable temperature from the 1.8–3.0 K range at H_{dc} = 1 kOe. The lines are only to guide the eye.

Discussion on magnetic properties of 2–4 (Comment to Figures S27–S29)

The direct-current (*dc*) magnetic characteristics of **2**–**4** are presented in Figure S27. The room temperature value of the magnetic susceptibility–temperature product, $\chi_M T$ for **2** is 0.73 $cm^3mol^{-1}K$, which is very close to the value of 0.80 $cm^3mol^{-1}K$ expected for the isolated free Ce^{3+} ion with the $^2F_{5/2}$ ground multiplet.[S8,S9] Upon cooling, the $\chi_M T$ decreases slowly down to 0.66 $cm^3mol^{-1}K$ at 20 K. Below 20 K, there is a more significant decrease of the $\chi_M T$ down to 0.61 $cm^3mol^{-1}K^{-1}$ at 1.8 K. Molar magnetization of **2** at 1.8 K shows the monotonous increase upon the increasing magnetic field reaching 1.2 μ_B at 70 kOe. These magnetic characteristics can be mainly ascribed to the single-ion properties of the Ce^{III} complexes embedded in **2** which is the gradual cooling-induced depopulation of the excited m_J levels within the $^2F_{5/2}$ ground multiplet. Due to the separation of the Ce^{III} centers by diamagnetic hexacyanidoruthenate(II) metalloligands, the inter-lanthanide magnetic interactions are canceled, and their minor role can appear only at the lowest temperatures below 10 K.

For **3**, the $\chi_M T$ reaches 0.48 $cm^3mol^{-1}K$ at 212 K (the highest accessible temperature of the reliable measurement due to the overall very weak magnetic signal) and decreases nearly linearly upon cooling down to the very low value of 0.03 $cm^3mol^{-1}K$ at 1.8 K. The room-temperature value is much higher than the 0.09 $cm^3mol^{-1}K$ expected for the isolated Sm^{3+} ion with the $^6H_{5/2}$ ground multiplet; however, this is a typical case as the Sm^{3+} ions reveal the closely lying excited ^6H_J multiplets that contribute to the overall magnetic signal at high temperatures.[S8,S9] At 1.8 K, the molar magnetization of **3** monotonously increases reaching 0.13 μ_B at 70 kOe without saturation which is typical for isolated lanthanide(III) centers. The observed characteristics can be reasonably explained by the single-ion properties of the Sm^{III} complexes, that is the cooling-induced depopulation of the excited ^6H_J multiplets and further higher-lying m_J levels within the ground multiplet. No sign of inter-lanthanide magnetic interactions is observed, as expected for the Sm^{III} centers with the very low magnetic moment, additionally being separated in the framework by the diamagnetic cyanido complexes.

At room temperature, the $\chi_M T$ for **4** is 12.1 $cm^3mol^{-1}K$, which is very close to the value of 11.8 $cm^3mol^{-1}K$, expected for the isolated free Tb^{3+} ion with the $^7F_6$ ground-state multiplet.[S8,S9] This value only weakly decreases upon cooling down to 50 K, while the abrupt decrease of $\chi_M T$ value is observed, down to 4.26 $cm^3mol^{-1}K$ at 1.8 K. The field-dependent magnetization curve shows the monotonous increase up to the value of 5.2 μ_B at 70 kOe. Similar to **2** and **3**, these dc magnetic characteristics can be mainly assigned to the single-ion properties of the Tb^{III} complexes present in the structure of **4**; however, the relatively large drop of the magnetic signal at the lowest temperatures can be partially assigned to the not fully suppressed magnetic interactions between lanthanide ions through the diamagnetic hexacyanidoruthenate(II) linkers.

As **2**–**4** contain the lanthanide(III) complexes that can reveal the substantial magnetic anisotropy leading to the Single-Molecule Magnet (SMM) behavior,[S9] we tested the related alternate-current (*ac*) magnetic properties. Compounds **3** and **4** do not exhibit the noticeable signal of the out-of-phase magnetic susceptibility, χ''_M in the accessible frequency range of 1–1000 Hz at 1.8 K which indicates the lack of slow magnetic relaxation effects that are characteristic of the SMMs. This indicates the poor magnetic anisotropy of the related Sm^{III} and Tb^{III} complexes, respectively. The onset of slow magnetic relaxation is observed in the Ce^{III}-containing **2** through the non-negligible χ''_M signal in the highest frequency range under the applied *dc* field at 1.8 K (Figure S28). The temperature-variable *ac* magnetic data for the optimal *dc* field of 1000 Oe shows the shift of the χ''_M signal toward higher frequencies upon heating leading to the

complete disappearance of the signal in the accessible frequency range above 2.6 K (Figure S29). This indicates non-negligible but rather weak magnetic anisotropy of the Ce^{III} complexes. No maxima on the $\chi''_M(\nu)$ are detected in the accessible frequency range which precludes the more detailed analysis. There is also a second, slower relaxation process that occurs in **2** for the high *dc* magnetic fields (Figure S28); however, it does not shift on the frequency scale upon the increased *dc* field or temperature suggesting that it is most probably due to the magnetic dipolar interactions, not related to the single-ion properties of the Ce^{III} centers.[S10] These results show that the investigated lanthanide complexes in **2**, **3**, and **4**, exhibit very weak magnetic anisotropy. This can be correlated with the respective coordination environments (Figure 1) involving cyanido and aqua ligands within the complexes of high coordination numbers leading to rather long metal-ligand distances. Therefore, there is no clear source of the modulation of the 4f-metal ion electron density (there is a lack of, e.g., strongly coordinating negatively charged ligands) that could provide the distinct SMM effect.[S9,S11]

References to Supporting Information

[S1] Avila, M.; Vargas, C.; Yee-Madeira, H.; Reguera, E. Titanium^{3+} Hexacyanometallates(II): Preparation and Porous Framework. *Z. Anorg. Allg. Chem.* **2010**, *636*, 1968–1973.

[S2] Reczyński, M.; Nowicka, B.; Näther, C.; Kozieł, M.; Nakabayashi, K.; Ohkoshi, S.; Sieklucka, B. Dehydration-Triggered Charge Transfer and High Proton Conductivity in $(H_3O)[Ni^{II}(cyclam)][M^{II}(CN)_6]$ (M = Ru, Os) Cyanido-Bridged Chains. *Inorg. Chem.* **2018**, *57*, 13415–13422.

[S3] Lopez, N. L.; Rodriquez-Hernandez, J.; Reguera, L.; Regueara, E. New Cubic Phases for $T_2M[CN]_6{\cdot}xH_2O$ with T = Ni, Cu and M = Ru, Os: Improving the Robustness and Modulating the Electron Density at the Cavity Surfaces. *Eur. J. Inorg. Chem.* **2019**, 3023–3032.

[S4] Llunell, M.; Casanova, D.; Cirera, J.; Bofill, J.; Alemany, P.; Alvarez, S.; Pinsky, M.; Avnir, D. *SHAPE v. 2.1. Program for the Calculation of Continuous Shape Measures of Polygonal and Polyhedral Molecular Fragments*, University of Barcelona: Barcelona, Spain, 2013.

[S5] Casanova, D.; Cirera, J.; Llunell, M.; Alemany, P.; Avnir, D.; Alvarez, S. Minimal Distortion Pathways in Polyhedral Rearrangements. *J. Am. Chem. Soc.* **2004**, *126*, 1755–1763.

[S6] Alvarez, S.; Alemany, P.; Casanova, D.; Cirera, J.; Llunell, M.; Avnir, D. Shape maps and polyhedral interconversion paths in transition metal chemistry. *Coord. Chem. Rev.* **2005**, *249*, 1693–1708.

[S7] Altomare, A.; Cuocci, C.; Giacovazzo, C.; Moliterni, A.; Rizzi, R.; Corriero, N.; Falcicchio, A. EXPO2013: a kit of tools for phasing crystal structures from powder data. *J. Appl. Cryst*. **2013**, *46*, 1231–1235.

[S8] Kahn, O. *Molecular magnetism*, VCH Publishers, Inc., New York, U.S., 1993.

[S9] Gatteschi, D.; Sessoli, R.; Villain, J. *Molecular Nanomagnets*, Oxford University Press, Oxford, United Kingdom, 2006.

[S10] Chorazy, S.; Wang, J.; Ohkoshi, S. Yellow to greenish-blue colour-tunable photoluminescence and 4f-centered slow magnetic relaxation in a cyanido-bridged Dy^{III}(4-hydroxypyridine)–Co^{III} layered material. *Chem. Commun.* **2016**, *52*, 10795–10798.

[S11] Rinehart, J. D.; Long, J. R. Exploiting single-ion anisotropy in the design of f-element single-molecule magnets. *Chem. Sci.* **2011**, *2*, 2078–2085.
